# Synthesis of Hydroxyaromatic Carboxylic Acids via Homogeneous Kolbe-Schmitt Carboxylation of Phenoxides

**DOI:** 10.3390/molecules31020239

**Published:** 2026-01-10

**Authors:** Dmitriy A. Merzliakov, Michael S. Alexeev, Maxim A. Topchiy, Dmitry G. Yakhvarov, Nikolai Yu. Kuznetsov, Anton L. Maximov, Irina P. Beletskaya

**Affiliations:** 1 A.V. Topchiev Institute of Petrochemical Synthesis, Russian Academy of Sciences, Leninsky Prospect 29, 119991 Moscow, Russia; mda@ips.ac.ru (D.A.M.); alekseev@ips.ac.ru (M.S.A.); maxtopchiy@ips.ac.ru (M.A.T.); max@ips.ac.ru (A.L.M.); beletska@org.chem.msu.ru (I.P.B.); 2 Faculty of Chemistry, Lomonosov Moscow State University, Leninskie Gory 1, 119991 Moscow, Russia; 3 Federal Research Center Kazan Scientific Center of Russian Academy of Sciences, Lobachevskogo st. 2/31, 420111 Kazan, Russia; yakhvar@iopc.ru

**Keywords:** carboxylation, CO_2_, phenoxides, Kolbe–Schmitt, hydroxyaromatic carboxylic acids (HACA), DMSO, DMF

## Abstract

Homogeneous Kolbe-Schmitt carboxylation of phenoxides offers a mild and effective alternative to the classical high-temperature solid-phase Kolbe-Schmitt reaction. To develop this into a practical synthetic approach, we investigated several fundamental dependencies, particularly the impact of cations (Na, K, Li, Cs, and Rb), phenoxide concentration, and solvents (DMSO or DMF) on the yield and regioisomeric ratio of hydroxyaromatic carboxylic acids (HACAs). We identified optimal conditions for the effective carboxylation of different phenoxides, including a chiral Ellman’s sulfinamide derived from *ortho*-vanillin. Both solvents and cations were found to be crucial in the carboxylation of phenoxides. Due to solvation effects, DMSO directs CO_2_ attack to the para-position of phenoxide, while DMF, although less selective, generally affords higher HACA yields. The addition of equiv. amounts of mesitolate salt to phenoxide in either DMSO or DMF solution often drives the reaction to completion, resulting in yields of up to 98%. Phenoxides containing several EWG groups, such as halogens or alkyl groups, adjacent to the reaction center show considerably lower reactivity in carboxylation; however, by carefully adjusting parameters, acceptable conversions (>70%) can be achieved. Using the gasometry, we assessed the stability of phenoxide and mesitolate carbonate complexes in DMSO. These experiments revealed distinct stages for the onset of decomposition and carboxylation at atmospheric pressure, indicating a lower energy barrier in the homogeneous process. Further insight into carbonate complex behavior was obtained through DOSY and ^13^C NMR experiments, which support increased molecular association in solution and correlate with enhanced reactivity.

## 1. Introduction

**Scheme 1:**
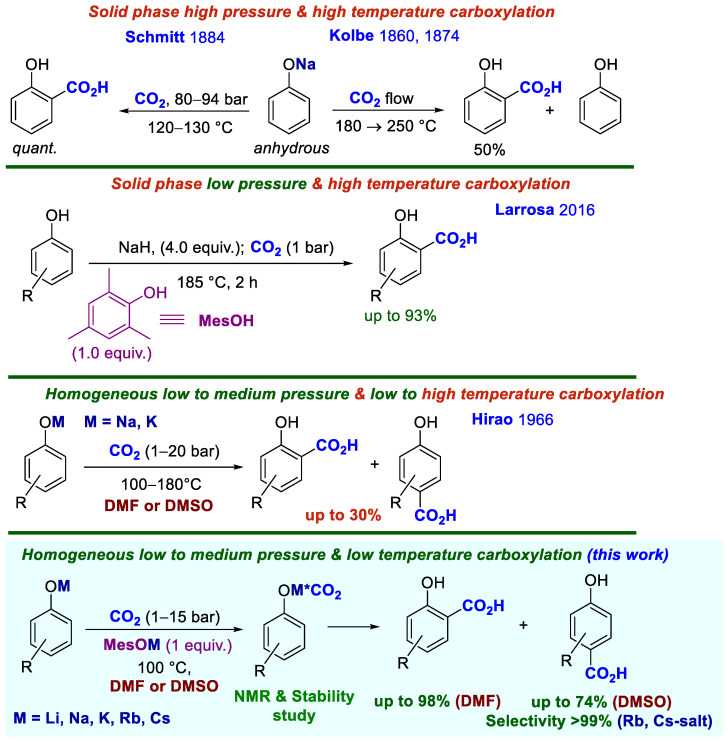
Development of the phenoxides carboxylation reaction. The * indicates uncertainty in the structure of the carbonate complex.

The acute climate problem of atmospheric CO_2_ accumulation has spurred a reevaluation of all industrial processes with high greenhouse gas emissions [1]. In turn, this opened up the opportunity for chemists to consider the CO_2_ molecule as a universal and virtually inexhaustible source of synthetic carbon-C_l_ [2,3,4,5,6,7]. The global scientific and industrial community has made enormous efforts to develop processes for extracting CO_2_ from air and industrial waste streams to convert it into valuable chemicals, such as carboxylic acids, methanol, carbonates, carbon monoxide, methane, and higher hydrocarbons. However, the high thermodynamic stability and low energy of CO_2_ require substantial energy input for its transformation, with costs rising as the degree of carbon reduction increases [3]. Consequently, only a limited number of CO_2_ conversion processes are economically viable up to day. Among these, the synthesis of hydroxyaromatic carboxylic acids (HACAs) via direct carboxylation of solid phenoxides—the Kolbe-Schmitt reaction—remains the oldest and most established method [8,9]. Since Kolbe’s initial discovery in 1860, carboxylation of phenoxide and Schmitt’s later high-pressure modification, numerous variants of this synthesis have been developed (Scheme 1) [10,11,12]. HACAs produced by the Kolbe-Schmitt reaction are essential industrial compounds, widely used as pharmaceuticals, agrochemicals, dyes, food preservatives, and technical products such as lubricants and antifreezes, as well as in polymer manufacturing [13,14].

However, despite its successful industrial history and the wide range of applications of such important products as HACAs, the Kolbe–Schmitt reaction remains a poorly understood phenomenon. This is primarily due to the inherent challenges of studying this solid-state process. The combination of inconvenient properties—heterogeneity, moisture sensitivity, the need for high temperature and pressure, poor reproducibility, and a lack of standardized conditions—has resulted in a contradictory reaction mechanism and unclear intermediate structures. These complexities acquire new data on this reaction, both scientifically relevant and vital.

We proposed that transferring the classical heterogeneous Kolbe-Schmitt reaction into a homogeneous phase, with the potential for greater standardization, could accelerate the understanding of the mechanism and enable the development of milder, more versatile methods for the carboxylation of phenoxides. This advancement is particularly crucial for fine organic synthesis, where substrates often contain chiral centers and sensitive functional groups. Indeed, many HACAs exhibit high biological activity and are valuable building blocks in medicinal chemistry [13,14,15,16,17,18,19].

The rationale for this possibility was provided by the previous works on the use of solvents as active media, solvating phenoxides and changing their reactivity in carboxylation reaction [20,21]. The work of Kosugi et al., [22,23] who demonstrated the fundamental feasibility of low-temperature carboxylation, alongside data on enzymatic carboxylation occurring under nearly physiological conditions [24]. Of particular interest was the report by Larrosa et al. [25], which highlighted the promoting effect of in situ-generated sodium mesitolate (sodium 2,4,6-trimethylphenoxide, MesONa) in the atmospheric-pressure carboxylation of phenoxides—a modern adaptation of the classical high-temperature Kolbe synthesis (Scheme 1).

While Hirao had previously shown that phenoxide carboxylation could be performed in homogeneous solution [21], the low efficiency of this approach prevented the development of a general method for homogeneous carboxylation (Scheme 1). In our recent work [26], which implemented the homogeneous carboxylation concept, we established quantitative parameters for water’s influence on the Kolbe-Schmitt reaction. We revealed nonlinear dependencies of both reaction yield and regioselectivity on phenoxide concentration and clearly demonstrated the promoting effect of basic salts, with sodium mesitolate proving most effective. For instance, the chemical yield of the 4-hydroxybenzoic acid (**4HBA**) and salicylic acid (**SA**) mixture from PhONa carboxylation increased up to twofold with 3 equiv. of sodium isopropylcarbonate and by 1.6-fold with 1 equiv. of MesONa. The advantage of working in solutions also allowed us to obtain spectral data on the intermediate carbonate complex of the phenoxide [sodium(benzo-15-crown-5)]. In the ^13^C NMR spectrum, the carboxylate carbon resonance appeared unusually upfield at 142 ppm, relative to typical carbonates, providing direct evidence for the postulated “elusive” carbonate complex.

The present study continues this investigation, offering new insights into fundamental data on the reactivity of lithium, rubidium, and cesium phenoxides in DMSO and DMF and the influence of a number of factors on this process; into the relationship between the stability of intermediate carbonate complexes, their reactivity, and the donor-acceptor ability of substituents in the aromatic ring. During this work the interplay between phenoxide concentration, carboxylation yield, regioisomeric composition, and molecular association of carbonate complexes in solution was investigated. Finally this study culminates in the development of a mild and general method for synthesizing hydroxyaromatic carboxylic acids (HACAs) via a homogeneous Kolbe-Schmitt reaction. (Scheme 1).

This material is divided into the following sections:

1. Study of the influence of Li, Cs and Rb cations on the yield and regioselectivity of carboxylation of phenoxides in DMSO;
2. Effect of DMF solvent on the yield and regioselectivity of carboxylation of phenoxides;
3. Gasometric determination of the stability of carbonate complex of potassium phenoxide in DMSO solution;
4. NMR study of the influence of concentration of carbonate complex of phenoxide on its molecular association in solution and registration of spectral characteristics of various carbonate complexes in DMSO solution;
5. Synthetic application of the homogeneous carboxylation method in the synthesis of HACAs.

## 2. Results and Discussion

### 2.1. Study of the Influence of Li, Cs, and Rb Cations on the Yield and Regioselectivity of Carboxylation of Phenoxides in DMSO

**Scheme 2:**
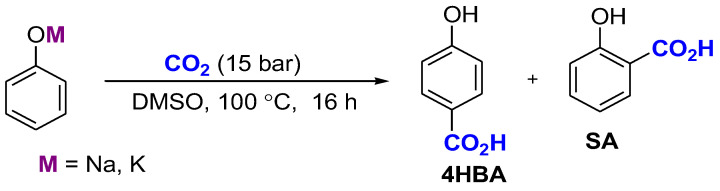
Carboxylation of phenoxides in DMSO.

**Figure 1:**
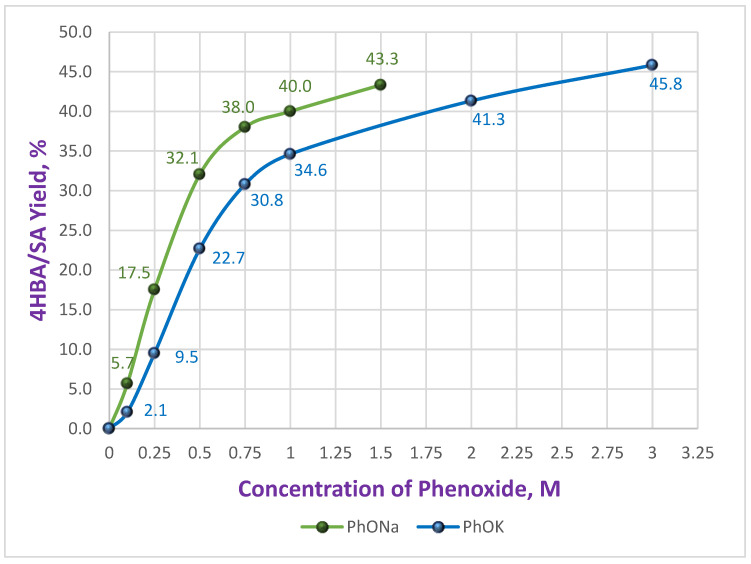
Dependence of the **4HBA**&**SA** yield on the initial concentration of Na and K phenoxides [26].

**Figure 2:**
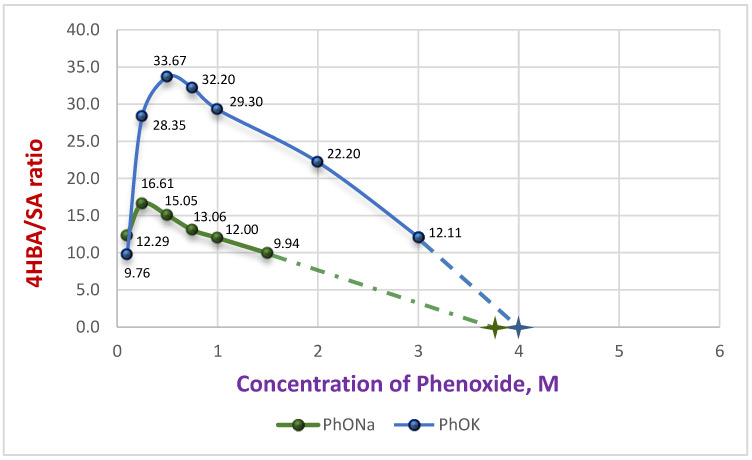
Dependence of **4HBA**/**SA** ratio on initial concentration of PhOM with extrapolation to the *X*-axis [26].

As demonstrated in our earlier work [26], the choice of the metal cation (Na^+^ or K^+^) in the phenoxide salt has a significant impact on both the chemical yield and regioselectivity of carboxylation in DMSO for the production of hydroxybenzoic acids. These dependencies were represented graphically, revealing distinct nonlinear, concentration-dependent patterns. The regioselectivity curves further exhibited extrema in the range corresponding to the maximum carboxylation rate. (see Scheme 2, Figure 1 and Figure 2).

Building on our hypothesis that these trends could be explained by the formation of molecular associates derived from phenylcarbonate, we investigated the behavior of phenoxides containing other alkali metal cations: Cs, Rb, and Li. Phenoxides of Cs and Rb were synthesized by reacting the corresponding commercial hydroxides with phenol, followed by drying (see Supplementary Materials). Li-phenoxide was prepared according to a previously reported method [26] and introduced into solution as its carbonate complex, as pure Li-phenoxide exhibits extremely poor solubility in DMSO (~0.34 mM at 25 °C, determined by HPLC). Employing our previously described method for evaluating phenoxides’ reactivity in small vials under uniform conditions in a 700 mL steel high-pressure reactor ([26], see also Supplementary Materials, Photo S1), we conducted a series of reactions. The resulting mixtures were analyzed quantitatively via reversed-phase HPLC.

**Figure 3:**
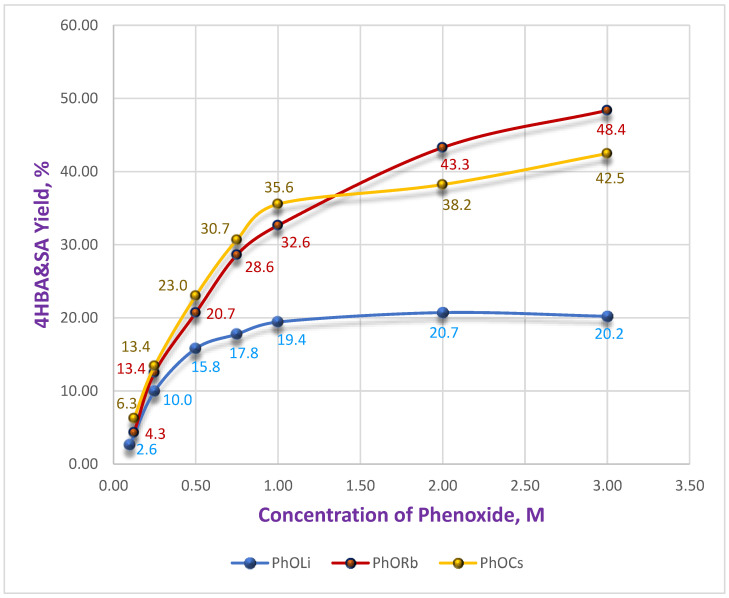
Dependence of the **4HBA**&**SA** yield on the initial concentration of Li, Cs and Rb phenoxides.

As shown, Li-phenoxide has the lowest reactivity across the entire concentration range (0.125–3.0 M) (Figure 3).

**Figure 4:**
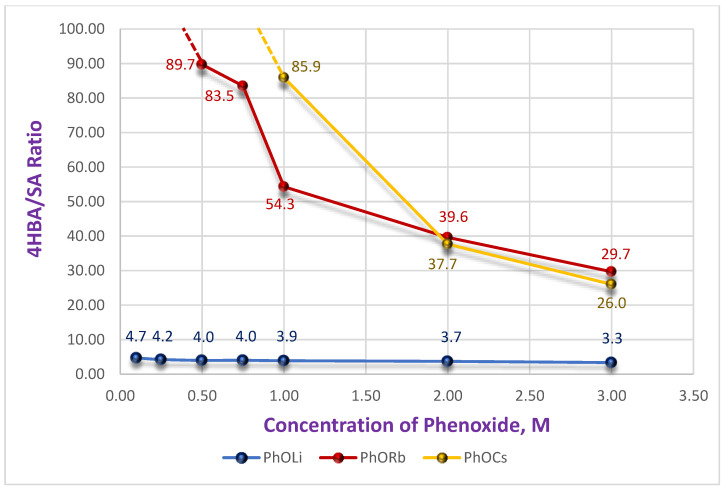
Dependence of **4HBA**/**SA** ratio on initial concentration of PhOM with extrapolation to undetectable level of **SA**.

The yield curve (blue) reaches a plateau at 1.0 M PhOLi and remains constant (~20%) for concentrations up to 3.0 M. A similar constancy is observed for PhOLi in the graph showing the dependence of the ratio of isomeric acids **4HBA/SA** (Figure 4). At low PhOLi concentrations (up to 0.75 M), the 4HBA/SA ratio is only 4.7–4.0. With an increase in concentration to 3.0 M it changes only marginally to 3.3. This behavior is consistent with a constancy of coordination sphere for the Li-ion, which remains essentially unchanged with concentration—a consequence of its strong Lewis acidity. A similar effect of rapid saturation of the coordination sphere in DMSO, as reflected in the activity coefficients, has been reported for lithium nitrate compared with sodium and potassium salts [27].

The behavior of Cs and Rb phenoxides differs sharply from PhOLi. It is nearly identical to that of PhOK (see Figure 1 and Figure 3), with the difference that PhORb is slightly more reactive, and PhOCs is somewhat less reactive, than PhOK. Maximum yields of 42.5% and 48.4% were obtained for Cs and Rb solutions, respectively, at 3.0 M.

However, significant differences from both potassium and sodium salts emerge when examining the **4HBA**/**SA** ratio (Figure 4; compare with Figure 1).

At low concentrations (≤0.25 M), **SA** was undetectable in the reaction mixtures for Cs and Rb phenoxides, even after HPLC analysis of concentrated samples.

**Scheme 3:**
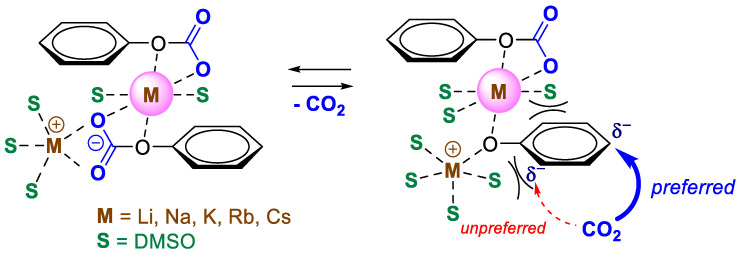
Suggested intermediates in the homogeneous carboxylation of phenoxide.

At a concentration of 0.5 M solution, the **4HBA**/**SA** ratio for Rb was 90:1. In contrast for Cs, **SA** remained undetected until reaching 1.0 M, where **4HBA**/**SA** = 86:1, while for Rb it was 54:1. At higher concentrations, the isomer ratios converged for both salts, reaching 38–40:1 at 2.0 M and 26–30:1 at 3.0 M. Even at these concentrations, the regioselectivity remained several times higher than that achieved with the potassium salt. This effect could be of practical value for achieving high regioselectivity in the carboxylation of substituted phenoxides. The observed trend suggests that the coordination ability of the cation in DMSO solution (Li < Na < K < Rb < Cs) [28] plays a key role in the observed dependencies. Considering our earlier assumption that two molecules of the carbonate complex (and/or phenoxide) participate in the transition state [26], we can draw the following Scheme 3.

It can be suggested that an ionic associate with solvated cations and anions of the carbonate complex exists in solution. When heated, one (or both) of the carbonate complex molecules loses CO_2_, forming an active phenoxide, which undergoes carboxylation. Depending on the solvation shell of the cation, steric hindrances of varying levels arise in the *ortho*-position of the phenol ring. In the case of DMSO, the growth of the solvation shell and, accordingly, steric hindrances in the *ortho*-position increase with the size of the cation, which directs the carboxylation to the *para*-position.

### 2.2. Effect of DMF Solvent on the Yield and Regioselectivity of Carboxylation of Phenoxides

Comparing the regioselectivity curves of Na-Cs phenoxides, one can note that with increasing concentration, the proportion of *ortho*-carboxylation leading to **SA** increases. Because of such behavior, an analogy arises with classical solid phase Kolbe-Schmitt reaction, when by concentrating the reaction mixture, in the limit, we can arrive at the selective formation of only **SA**. Extrapolating the regioselectivity curves of sodium and potassium phenoxides to the *X*-axis, it can be assumed that already in the range of 4–5 M solutions, regioselective formation of **SA** is expected (see Figure 2). However, such concentration values are difficult to obtain experimentally, due to the insufficient solubility of phenoxides in DMSO. Therefore, we also studied the carboxylation of PhONa in dried DMF (water content <15 ppm), where phenoxides are more soluble. Mixtures of PhONa and DMF were obtained in various concentrations from 1 to 5 M and even more. For ease of calculation, molar ratios of PhONa/DMF rather than concentrations were used: 1:11.4 (1 M), 1:4, 1:3, 1:2.5 (5 M), 1:2, 1:1.5, 1:1, 3:1, and 5:1.

The test reactions were carried out under standard conditions at 100 °C and an initial CO_2_ pressure of 15 bar for 3 h. The composition of the reaction mixtures was quantitatively analyzed by HPLC (Table 1).

**Figure 5:**
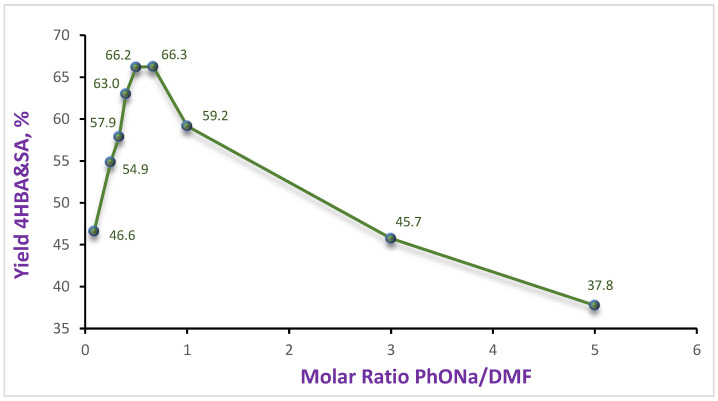
Dependence of the **4HBA**&**SA** yield on the molar ratio of PhONa in DMF.

The initial experiment with a 1 M solution showed a relatively high **SA** content in the mixture (see Table 1, n. 1, 2). Additionally, the reaction proceeded faster, and the yield of the acid mixture was higher than in DMSO ([26] in 1 M—40%; 1.5 M—43% for 16 h). A gradual increase in the concentration of PhONa led to an increase in the **SA** content and, consequently, to a rise in the total yield to 66% (Table 1, n. 1–6). It should be noted that up to a DMF/PhONa ratio of 2.0–1.5:1, the absolute content of **4HBA** (32–34%) remained virtually unchanged. Further reduction in the DMF quantity led to a gradual decrease in the total yield to 37.8% due to a sharp reduction in the **4HBA** amounts (Table 1, n. 7–9). The graphical representation of the obtained results on the total yield is shown in Figure 5, which exhibits a characteristic maximum in the range of ratios 1:1.5–2.0.

Since a 3 h period was initially used as the test time to evaluate the reaction yield, this time was extended to 16 h at an optimal PhONa/DMF ratio (1.0:2.0). To our delight, the total yield of **4HBA&SA** reached almost 84%. The regioselectivity of PhONa carboxylation in DMF differs significantly from that in DMSO, with a higher proportion of **SA** formed even in a dilute 1 M solution. As the concentration increases, the **4HBA/SA** ratio first equalizes (see n 1–5), and then **SA** begins to predominate in the mixture. However, a further reduction in the amount of solvent and the transition of the reaction to a heterogeneous solid phase process result in the termination of carboxylation. Actually, the reaction proceeds locally at the place where the solvent is introduced. At the same time, the unsolvated part of the solid sodium phenoxide (phenylcarbonate) remains unchanged, without entering into the carboxylation reaction of the aromatic system. The differences in the regioselectivity of carboxylation in DMF and DMSO are in good agreement with the decrease in standard partial molar volumes (V_0_) of alkali cations [29], particularly for Na^+^ (−2 cm^3^ mol^−1^/DMF vs. 3 cm^3^ mol^−1^/DMSO), K^+^ (6 cm^3^ mol^−1^/DMF vs. 11 cm^3^ mol^−1^/DMSO) and Cs^+^ (17 cm^3^ mol^−1^/DMF vs. 23 cm^3^ mol^−1^/DMSO). These differences in V_0_ would be responsible for more tight steric environment in molecular associates confirming our hypothesis about the influence of cation solvation on regioselectivity. Moreover, DMSO provides stronger solvation of charged molecules as known from its electron donor DN (Donor Number) DMSO = 29.8 and DMF = 26.6 as well as electron acceptor abilities AN (Acceptor Number) DMSO = 19.3 and DMF = 16.0 [30]. These solvation properties would limit accessing of CO_2_ towards oxygen and *ortho*-site in phenoxide.

Thus, although our attempt to connect homogeneous and solid-phase processes was largely ineffective, DMF is a successful solvent that increases phenoxide conversion and promotes more efficient *ortho*-carboxylation.

### 2.3. Gasometric Determination of the Stability of Carbonate Complexes of Phenoxides in DMSO Solution

**Figure 6:**
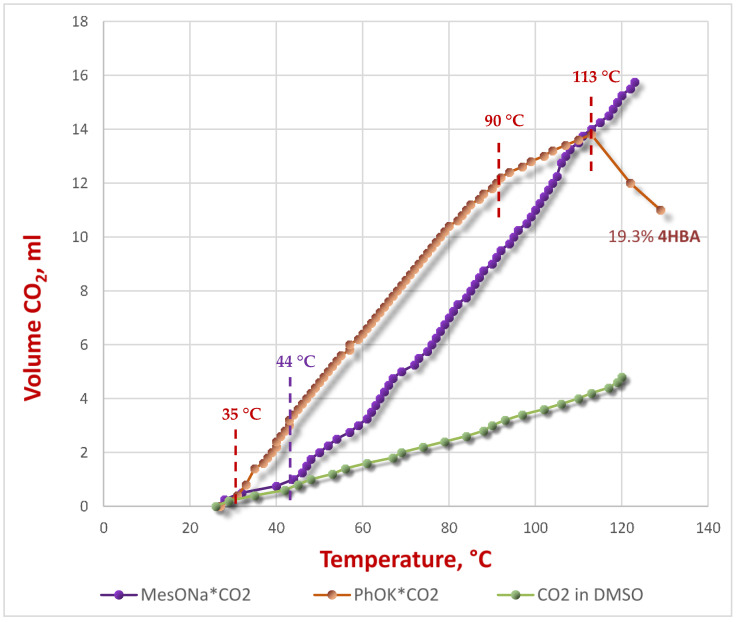
Results of gasometry measurements in DMSO.

One of the aims of our work was to determine the stability of the PhOK*CO_2_ complex in a DMSO solution. It was proposed that the carboxylation of the phenoxide occurs in the anionic form, and the conditions of the intermediate carbonate’s decomposition are among the influencing factors [22,26]. For this purpose, we used the gasometry method, in which we recorded the volume of CO_2_ evolved from a 1 M solution of the PhOK*CO_2_ along with a gradual increase in temperature. To eliminate the influence of dissolved CO_2_ and its thermal expansion, comparative measurements of CO_2_ evolution from a saturated DMSO solution were conducted under identical conditions, including thermostatting of the gas meter’s measuring tube. Additionally, we measured the decomposition of the MesONa*CO_2_ complex, yielding the following graphical dependences (Figure 6).

The analysis of the decomposition curve for PhOK*CO_2_ reveals three clearly separated stages of decomposition. The first stage begins at 35 °C, marking the onset of decomposition of the carbonate complex. At 90 °C, a bend appears on the curve, indicating a slower release of CO_2_, similar to the rate of CO_2_ release dissolved in DMSO. This stage continues until the temperature reaches 113 °C, at which point a second bend appears, accompanied by rapid CO_2_ absorption from the gasometer. HPLC analysis of the endpoint gasometric measurement shows the formation of 19.3% **4HBA** after 38 min at atmospheric CO_2_ pressure. Gasometry demonstrates that the carbonate complex in solution begins to decompose at a much lower temperature than in a classical heterogeneous system. Notably, Dinjus et al. demonstrated [31] that decomposition of the solid carbonate complex occurs at 75–80 °C. It was shown that this process is not accompanied by the formation of **SA**, but represents the decarboxylation of the complex. Hirao and Kito also revealed [32] that thermal decomposition of the PhOK*CO_2_ occurs above 70 °C.

The stage of slowing CO_2_ release at 90 °C apparently corresponds to both the completion of the carbonate complex decomposition and the onset of carboxylation of phenoxide at atmospheric pressure. The third bend in the curve after 113 °C is apparently due to the carboxylation reaction, which is confirmed by the formation of noticeable amounts of **4HBA** during this short period.

The decomposition of the solution of the carbonate complex MesONa also exhibits a characteristic form. In this case, the complex decomposes only at 44 °C, indicating its greater stability. This is explained by the greater donor properties of the aryl fragment, which stabilize the carbonate form. Interestingly, the *ortho*-methyl groups of mesitolate do not significantly affect the complex’s stability. A further increase in temperature shows only a gradual decomposition of the complex without a carboxylation reaction, which, in principle, is not realizable in mesitolate.

### 2.4. NMR Study of the Influence of Concentration of Carbonate Complex of Phenoxide on Its Molecular Association in Solution and Registration of Spectral Characteristics of Various Carbonate Complexes in DMSO Solution

#### 2.4.1. Molecular Weight Determination of Carbonate Complex of PhONa by DOSY

The study of concentration dependences of the carboxylation of phenoxides (yield of hydroxybenzoic acids and their regioisomeric composition) is consistent with the participation of non-monomeric phenoxide molecules in the carboxylation reaction. To investigate the proposed formation of molecular associates in solutions of phenoxides (or their carbonate complexes) in DMSO, we used diffusion-ordered NMR spectroscopy (DOSY), which enables accurate molecular mass determination. For this purpose, solutions of PhONa*CO_2_ were prepared in dry DMSO-D_6_ (dried over 4Å molecular sieves) with concentrations of 0.036 M, 0.143 M, 0.250 M, and 0.357 M. Uncharged standard compounds with different molecular weights (Ph_3_PO (278 g/mol), 1,1′-bis(diphenylphosphino)ferrocene (dppf, 554 g/mol) and standard polystyrene (PS, 1320 g/mol)) that do not form associates were added to each solution, and residual DMSO-D_5_ (83 g/mol) was used as low-molecular weight standard. From the DOSY spectra, the diffusion coefficients of the standards and the carbonate complex PhONa were determined, and the dependence of the decimal logarithm of the diffusion coefficients on the decimal logarithm of the molar masses of the standards was constructed. According to the Stokes-Einstein equation, the diffusion coefficient of a substance in a solution is directly proportional to the temperature and inversely proportional to the viscosity of the medium and the hydrodynamic radius, which is proportional to the molar mass of the substance.

(1) $$D\;=\;\frac{k_{B}T}{6\pi \eta R_{s}}\;\Rightarrow \;D∼\;\frac{1}{R_{s}}∼\;\frac{1}{\sqrt[{3}]{M}}\;\Rightarrow \;{log}_{10}D\;∼\;-{log}_{10}M$$

Equation (1): Equation for bonding diffusion coefficient and molecular weight

**Figure 7:**
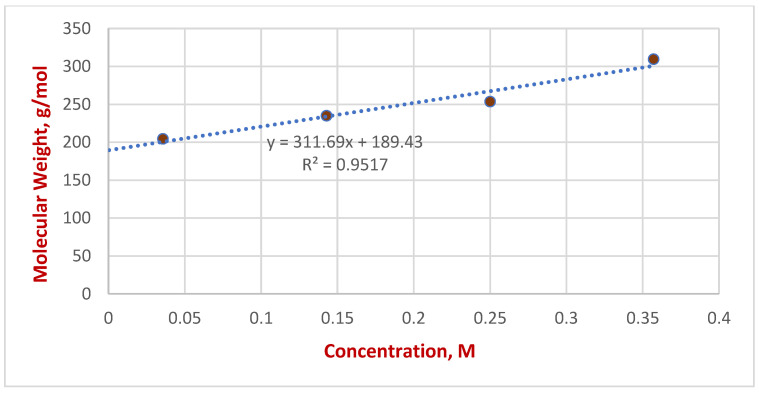
Dependence of the molecular mass determined by the DOSY NMR method on the concentration of the carbonate complex PhONa.

The corresponding dependences of diffusion coefficients on molecular weight were obtained. For the obtained dependencies, linear approximations were constructed at each concentration (see Supplementary Materials), with determination coefficients (R^2^) of 0.991, 0.977, 0.993, and 0.995, respectively, indicating a good fit of the data by a linear function. From the obtained diffusion coefficients, the apparent molecular mass of the PhONa carbonate complex in a DMSO-D_6_ solution was determined using approximated straight lines. For solutions of the carbonate complex PhONa with concentrations of 0.036M, 0.143M, 0.250M, and 0.357M, the molecular masses obtained were 204.5, 234.9, 253.6, and 309.6 g/mol, respectively. Next, the dependence of the molecular mass determined by the DOSY NMR method on the concentration of the carbonate complex PhONa was constructed (Figure 7). The theoretical value of molecular weight of the monomeric form of the carbonate complex (PhONa*CO_2_) is 160.1 g/mol, though in a solution sodium ion will be solvated with several molecules of DMSO-D_6_ and observed molecular weight will be higher. The exact molecular weight of solvated cations is difficult to estimate because solvent molecules are in dynamic exchange. For the obtained dependence of the observed molar mass of sodium phenyl carbonate on its concentration, a linear approximation was constructed, the angular coefficient of which was 311.7, and the free coefficient was equal to 189.4. The equation of the approximating line is: MWt = 311.7*C + 189.4, R^2^ = 0.95. From the obtained line, one can conclude that the observed molecular mass increases with the increasing concentration of PhONa*CO_2_ in the solution.

The value of the free term of the obtained approximating line is 189.4. This dependence may indicate a dynamic equilibrium and the formation of molecular associates consisting of phenyl carbonate anions, sodium cations and DMSO (DMSO-D_6_). The slope of the approximating line is 311.7, from which it follows that, for each unit increase in concentration, the average molecular weight increases by 311.7 g/mol. For example, at 1.0 M concentration, the molecular weight of the phenyl carbonate complex will be about 501 g/mol, and at 3.0 M it can be about 1124 g/mol, maintaining linearity with increasing concentration. Particularly, the molecular weight of the dimeric carbonate complex without the second solvated cation is 465 g/mol (see Scheme 2).

Considering that the reaction efficiency increases most rapidly in the region of 0.75–1.0 Mol/L (see Figure 1 and Figure 3), where bimolecular associates predominate, it can be suggested that this composition of molecular associates is optimal for carboxylation in DMSO.

#### 2.4.2. NMR Characteristics of Various Carbonate Complexes in DMSO Solution

Another important problem that can be studied using the NMR method is the spectral characteristics of carbonate complexes on ^13^C nuclei. We believe that this information can be used in the future for quantum chemical modeling of the reactivity of carbonate complexes. In the preceding paper, we obtained the ^13^C NMR spectrum of a phenyl carbonate complex in which a crown ether moiety bound the sodium cation. The chemical shift in the carboxylate carbon was 142 ppm, significantly shifted to the upper field relative to the chemical shifts in typical stable carbonates (Table 2). Therefore, we concluded that the carbonate complex has an intermediate structure between a carbonate and dissolved CO_2_ [26]. In the present work, a series of new carbonate complexes **2a–j** were obtained from the corresponding phenoxides **1a–j** (Table 2) by saturating the phenoxide solutions with CO_2_ (10 bar) in a steel autoclave and subsequent NMR analysis at ambient pressure. For comparison, literature data are given for the chemical shifts in the carboxylate carbon atoms of solid potassium phenylcarbonate (Table 2, n 1), a saturated solution of CO_2_ in DMF (Table 2, n 2), and a phenylcarbonate complex with crown ether (Table 2, n 3).

The new method does not require the use of a crown ether, and for most compounds, data can be obtained specifically for carbonate complexes (Table 2, n 4–10; **2a–g**). The chemical shifts in alkyl-substituted phenyl carbonates **2b–2d**, **2f** are in the region of 148–149 ppm, and those of unsubstituted phenyl carbonate **2a** and 2,4-di*t*BuPh-carbonate **2e** are 142–143 ppm (Table 2). Apparently, the chemical shift in the carboxylate carbon is related to the stability of the carbonate complex. In compounds with a donor aromatic system, the chemical shift is shifted towards stable carbonates, and weakening of donor activity or steric factors (as in **2e**) leads to the opposite values. Methoxy-substituted carbonate **2g** shows a chemical shift at 137 ppm. Further deactivation of the aromatic system leads to the failure to fix a stable carbonate complex. The carbon atom signals become very close to dissolved CO_2_ both in position (Table 2, n 11–12, **2h,i**) and in shape (much narrower signals). However, we believe this is not purely dissolved CO_2_ but rather a form of its interaction with phenoxide. This is confirmed by the influence of CO_2_ pressure on chemical shift positions. At 4 bar (NMR ampoules for spectra under pressure), the signals of the carboxylate atom and the *ortho*-carbon atoms begin to shift, indicating interaction with CO_2_. The presence of a 4-chloro substituent in **2k** does not cause significant destabilization of the carbonate complex, shifting the carbon signal to 140 ppm (Table 2, n 14). At the same time, the 3-alkyl group enhances carbonate stability, as the **2o** chemical shift is highest for the 3-methyl derivative (Table 2, n 15).

Though the correlation of electronic properties of substituents with the stability of carbonate complexes (Figure 6) and chemical shifts in carboxylate carbon (Table 2) is quite clear, the correlation of reactivity with ^13^C chemical shifts is not evident. A more stable intermediate carbonate can be less reactive and vice versa. To determine the degree of mutual influence of the stability of the carbonate complex and its reactivity, four comparative carboxylation experiments were carried out with sodium phenoxide and three *ortho*-substituted phenoxides—2-chloro, 2-methoxy, and 2-allyl (100 °C, 1 h, 15 bar in DMSO). The choice of substrates was made in such a way as to, on the one hand, exclude the influence of the steric factor on the reaction center, and on the other hand, to ensure that the values of the chemical shifts in the carboxylate carbons lay on a line with decreasing values. In addition, the reaction time was reduced to 1 h to obtain non-completed reactions. As a result, the following values of chemical yield of hydroxybenzoic acids were obtained (Table 3).

The most stable allyl-substituted derivative **2f** provides a higher yield of acids **3f**&**4f** (50.8%), whereas the less stable non-substituted phenyl-derivative **2a** gave already 40% yield of **3a**&**4a** (Table 4). This trend is followed by the two remaining carbonates, **2g** and **2h**, yielding 37.2% and 10.2% of the corresponding acids. Thus, ^13^C chemical shifts can be convenient markers for assessing the reactivity of phenoxides.

### 2.5. Synthetic Application of the Homogeneous Carboxylation Method in the Synthesis of HACAs

**Scheme 4:**
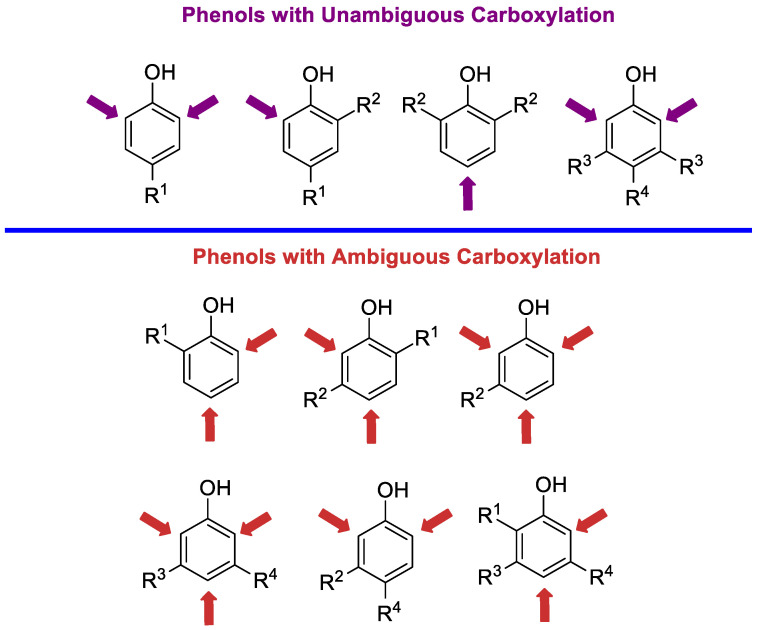
Direction of carboxylation reactions in substituted phenols.

Having in hand data on the reactivity of phenoxides under various conditions and understanding how to optimize yield and control selectivity during carboxylation, we prepared different phenols for homogeneous carboxylation. All compounds can be divided into two types: those with unambiguous and those with ambiguous regioselectivity (Scheme 4). Depending on the phenol group, the conditions for carboxylation should be selected. Of course, in the group of phenols with ambiguous selectivity, it is necessary to take into account the steric influence of substituents, which play an essential role in the regioselectivity of carboxylation.

To conduct synthetic experiments, phenoxides were prepared using various methods: (a) metal hydroxides (K, Na, Cs, Rb); (b) solutions of methylates in methanol (Li, Na, K); (c) sodium hydride in THF or DCM.

**Scheme 5:**
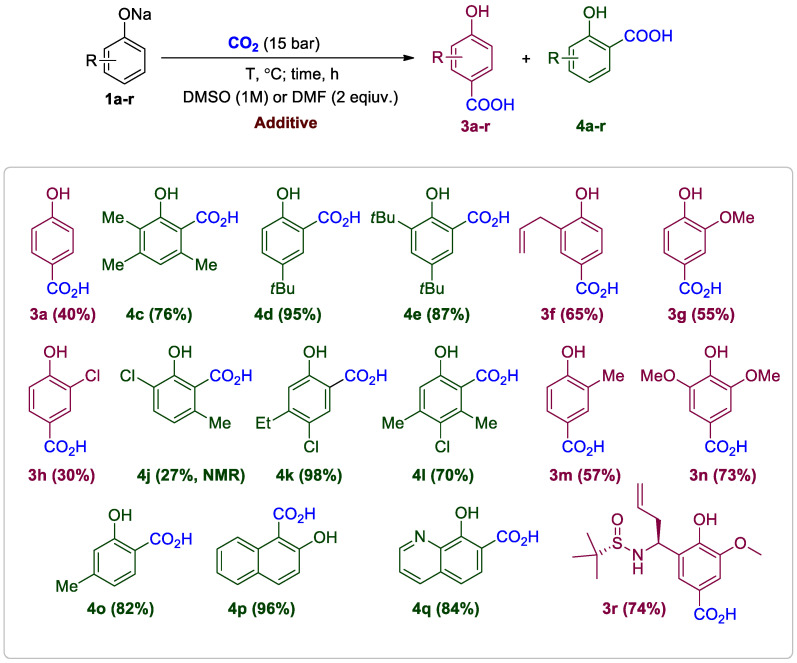
HACAs from phenoxides carboxylation in solvents.

In DMF, sodium phenoxide **1a** showed no selectivity (Table 4; also see Table 2). The best selectivity was observed with Cs- or Rb-phenoxides, where Cs-phenoxide efficiently yielded **4HBA 3a** (40%) when the reaction was carried out in DMSO. The acid was isolated from the reaction mixture through filtration. It was noted that obtaining the acid from sodium mesitolate **1b** through carboxylation was impossible. In the trimethyl-substituted phenol **1c**, two carboxylation pathways—*ortho* and *para*—were possible. However, the presence of two methyl groups at the *para* position of **1c** completely blocked the reaction along this pathway, resulting in the formation of only acid **4c** (Scheme 5, Table 4). The reaction rate was low due to the adjacent methyl group, requiring a combination of conditions for a good yield: the addition of MesONa **1b** in DMF and heating at 120 °C. Under these conditions, the yield of **4c** reaches 76%. When introducing only one *t*Bu substituent into the *para* position of the phenyl ring in **1d**, unambiguous carboxylation to the *ortho* position was expected. Carboxylation was first tested in DMSO, and to our surprise, the product—**4d** (5%)—was virtually absent (Table 4). It was found that carbonate associates in DMSO virtually blocked the *ortho* positions of phenol (see also Scheme 3), confirming the conclusion that the carboxylation pathways to the *ortho* and *para* positions are completely kinetically independent [26]. Replacing the solvent with DMF and using additive **1b** allows one to obtain acid **4d** in fairly quantitative yield (95%). Disubstituted phenol **1e** was also successfully carboxylated under these conditions, yielding **4e** (87%) (Scheme 5, Table 4). For mono-substituted ambiguous phenols **1f** and **1g**, the selectivity issue is resolved by using DMSO, while DMF did not provide such a possibility for these substrates. The corresponding *para*-substituted acids **3f** (65%) and **3g** (55%) were isolated by chromatography (Scheme 5, Table 4). The introduction of the acceptor chlorine substituent in **1h** significantly slowed down the reaction (even the carbonate complex is unstable, Table 2). Therefore, the yield in DMSO is low for **3h** (30%) (Scheme 5, Table 4). The unfavorable electronic properties of phenol **1j** were complemented by the steric influence of the methyl group, resulting in a low yield of **4j** (27%) even in DMF (Scheme 5, Table 4).

At the same time, the halogen at the 4-position of phenol **1k** does not interfere with carboxylation at all. Under optimized conditions in DMF with **1b** additive, **4k** is obtained almost quantitatively (98%) (Scheme 5, Table 4). Though **1k** is an ambiguous phenol (see Scheme 4), the ethyl group has sufficient steric effect for achieving complete regioselectivity. To compare the course of the classical solid-phase Kolbe-Schmitt reaction with **1k**, we performed the reaction without solvent at 150–160 °C. The acid **4k** (80%) was obtained for 2 h, but along with 1.8% of the regioisomeric *ortho*-acid. Clearly, milder carboxylation conditions in the solvent yield a cleaner reaction.

The carboxylation reaction of phenol **1l** proceeds unambiguously but slowly, similarly to **1c**, so the same conditions were used: MesONa **1b**, DMF, and heating at 120 °C. As a result, acid **4l** (70%) (Scheme 5, Table 4) was obtained and collected by simple filtration.

*ortho*-Cresol **1m** was selectively carboxylated in DMSO using its cesium salt, and the *para*-acid **3m** (57%) was isolated by chromatography. Perry and Kong reported similar effect of *para*-selectivity of cesium salt in carboxylation in DMF solution [33] (this paper appeared during our submission). Carboxylation of syringol salt **1n** (together with guaiacol **1g**, a product of lignin pyrolysis) in DMSO with **1b** additive smoothly yielded acid **3n** (73%). The unexpectedly lower yield in DMF of **3n** (39%) is likely due to the salt’s poorer solubility in this solvent. It should be noted that solubility affects the reaction yield and should be taken into account.

The reaction of the *meta*-cresol salt **1o**, carried out under our mild conditions, is highly indicative of selectivity. *meta*-Cresol is an ambiguous phenol and the observed earlier regioselectivity of its carboxylation was quite moderate—5.7:1 [25]. We believe this is due to the high reaction temperature. In our conditions, the reaction in DMF was very selective for the formation of **4o** (99:1, 70%). Using **1b** additive, the yield of **4o** was increased to 92% while maintaining the high selectivity of 97:3 of the carboxylation (Scheme 5, Table 4).

Phenoxides derived from condensed aromatic systems—2-naphtholate **1p** and 8-hydroxyquinolinate **1q**—were tested under the developed conditions. Sodium naphtholate **1p** yields acid **4p** (96%) almost quantitatively at 70 °C in DMSO (Scheme 5, Table 4). After optimization of the reaction conditions (DMF with **1b** additive), acid **4q** (84%) was obtained from **1q** in high yield, though previously the yield of **4q** in DMF under atmospheric CO_2_ pressure was lower, 34% [34].

In our method, the reaction conditions are much milder than those of the classical Kolbe-Schmitt method. This allows us to carboxylate not only typical phenols but also more sensitive compounds containing chiral centers capable of racemization. Specifically, we synthesized enantiomerically pure Ellman homoallyl amide **1r** from *ortho*-vanillin, converted it to the disodium salt, and carboxylated it in DMSO with **1b** (Scheme 5, Table 4). The reaction proceeds unambiguously, but quite slowly with **1r**, so we extended the reaction time to 24 h, yielding **3q** in good yield (74%) without the formation of stereoisomeric products.

## 3. Materials and Methods

### 3.1. Synthesis of Sodium Phenoxides

#### 3.1.1. Variation A (**1f**, **1g**, **1m**, **1n**, **1p**)

Sodium (569 mg, 24.8 mmol) was dissolved in dry MeOH (10 mL) from 0 to 25 °C. Phenol (25.0 mmol) was dissolved in a minimal volume of MeOH, then a MeONa solution was added. The solvent was distilled under reduced pressure (15 Torr) on a water bath (30 °C), then solid phenoxide salt was crushed into a powder under Ar. This powder was dried under vacuum (0.1 mbar) on an oil bath (200 °C) for 10 h. This extended heating is required to remove traces of phenols

#### 3.1.2. Variation B (**1c–1e**, **1h–1l**, **1o**, **1q**)

Under Ar atmosphere NaH (60% dispersion in mineral oil) (280 mg, 7.0 mmol) was washed trice with hexane (5 mL) to remove the oil. dry THF or DCM (10 mL) was added to the washed NaH powder, and phenol (7.0 mmol) was added portion wise. After 1 h, when H_2_ evolution was complete, the solvents were evaporated under reduced pressure (15 Torr), and sodium phenoxide was dried under vacuum (0.1 mbar) on an oil bath (200 °C) for 10 h. This extended heating is required to remove traces of phenols

### 3.2. Carboxylation Experimental Procedures

#### 3.2.1. Standard Procedure for the Carboxylation of Sodium Phenoxide in DMSO

Under Ar atmosphere sodium phenoxide (1.0 mmol) was poured as a solid from a bulk dry salt into a small glass vessel (3 mL) equipped with a magnetic stirrer bar. Anhydrous DMSO (1 mL) was added, and the vessel was placed into a steel pressure reactor (V = 20 mL, Photo S1). The reactor was pressurized under 15 bar of CO_2_ and placed into an oil bath (100 °C) with stirring for 16 h. After the completion of the reaction, the reactor was cooled, and CO_2_ pressure was slowly released (≈10 min). NaHCO_3_ (sat. solution) (2 mL, 2.0 equiv.) was added to the reaction mixture, and organic impurities were removed by extraction with EtOAc (3 × 5 mL). The aqueous layer was acidified to pH = 1 with conc. HCl, and the product was extracted with EtOAc (3 × 5 mL). The combined organic extract was washed with NaCl (sat solution) (1 × 5 mL), dried over Na_2_SO_4_ and evaporated to dryness. The pure product was obtained by recrystallization or column chromatography.

#### 3.2.2. Standard Procedure for the Carboxylation of Sodium Phenoxide in DMF

Under Ar atmosphere sodium phenoxide (1.0 mmol) was poured as a solid from a bulk dry salt into a small glass vessel (3 mL) equipped with a magnetic stirrer bar. Anhydrous DMF (155 µL, 2.0 mmol, 2.0 equiv.) was added, and the vessel was placed into a steel pressure reactor (V = 20 mL, Photo S1). The reactor was pressurized under 15 bar of CO_2_ and placed into an oil bath (100 °C) with stirring for 16 h. After the completion of the reaction, the reactor was cooled, and CO_2_ pressure was slowly released (≈10 min). NaHCO_3_ (sat. solution) (2 mL, 2.0 equiv.) and water (5 mL) were added to the reaction mixture, and organic impurities were removed by extraction with EtOAc (3 × 5 mL). The aqueous layer was acidified to pH = 1 with conc. HCl, and the product was extracted with EtOAc (3 × 5 mL). The combined organic extracts were washed with NaCl (sat solution) (1 × 5 mL), water (1 × 5 mL), dried over Na_2_SO_4_, and evaporated to dryness. The pure product was obtained by recrystallization or column chromatography.

### 3.3. Preparation and Carboxylation of a Chiral Ellman’s Sulfinamide with a Phenolic Group

#### 3.3.1. Synthesis of Ellman’s Imine

*ortho*-Vanillin (2.28 g, 15.0 mmol), (*S*)-2-methyl-2-propanesulfinamide (1.87 g, 15.5 mmol) and Ti(O*i*Pr)_4_ (17.1 g, 17.8 mL, 60.0 mmol) in THF (15 mL) were placed in a 50 mL flask under Ar. The flask was stoppered, and the solution was stirred while heating on an oil bath at 60 °C for 24 h. The resulting mixture was gradually poured into a mixture of water (180 mL) and DCM (50 mL) and stirred for 3 h until a yellowish precipitate formed. The resulting suspension was filtered through a Hyflo^®^ Super-Cell^®^ filter pad, the filter pad was carefully washed with DCM (3 × 20 mL). The organic phase was separated, the aqueous phase was extracted with DCM (20 mL), and the combined organic extracts were dried over MgSO_4_. The resulting solution was evaporated on a rotatory evaporator. The residual oil was recrystallized from hexane to give (*S*,*E*)-*N*-(2-hydroxy-3-methoxybenzylidene)-2-methylpropane-2-sulfinamide (3.38 g, 88%) as a beige solid, m.p. 118–120 °C (hexane), R_f_ 0.20 (hexane/EtOAc, 4:1). ^1^H NMR (CDCl_3_, 400 MHz): δ 11.25 (s, 1H, OH), 8.67 (s, 1H, CH=N), 7.07 (dd, 1H, *J* = 7.8, 1.4 Hz, Ar), 7.02 (dd, 1H, *J* = 8.1, 1.0 Hz, Ar), 6.90 (t, 1H, *J* = 7.9 Hz, Ar), 3.89 (s, 3H, OMe), 1.22 (s, 9H, *t*Bu) ppm. ^13^C NMR (CDCl_3_, 101 MHz): δ 165.4, 150.2, 148.4, 124.5, 119.5, 118.4, 116.0, 57.9, 56.2, 22.2 (3C) ppm. NMR spectra coincide with the literature data [35].

#### 3.3.2. Copper(I)-Catalyzed Diastereoselective Allylation of Ellman’s Imine

Ellman’s imine (10.0 mmol) and Cu(PPh_3_)_3_Cl*MeCN (0.463 g, 0.5 mmol) were dissolved in THF (25 mL) under Ar. To this solution at −15 °C were added *t*BuOK solution (1.0 M in THF, 0.5 mL, 0.5 mmol), Et_3_N (3.02 g, 4.18 mL, 30.0 mmol) and triallylborane- 1,4-diazabicyclo[2.2.2]octane adduct (1.23 g, 5.0 mmol, 0.5 equiv.) and the solution was stirred for 1.5 h at this temperature. A solution of MeOH (0.32 g, 0.4 mL, 10.0 mmol, 1.0 equiv.) in THF (10 mL) was added to the formed mixture via syringe pump over 1 h, after which the mixture was stirred for another 1 h and quenched with AcOH (3.0 mL, 50.0 mmol) at −15 °C. The mixture was diluted with EtOAc (15 mL) and a mixture of K_2_CO_3_ solution and 25% aq.NH_3_ (for copper extraction). The organic layer was separated and washed several times with a mixture of brine and 25% aq.NH_3_ until the blue coloration disappeared. The resulting extract was dried over MgSO_4_ and evaporated to give the crude homoallylamine as a viscous yellow oil. This product was purified by column chromatography (hexane/EtOAc 1:1) to afford (*S*)-*N*-((*S*)-1-(2-hydroxy-3-methoxyphenyl)but-3-en-1-yl)-2-methylpropane-2-sulfinamide (2.56 g, 86%) as a white solid, m.p. 87–89 °C (hexane/EtOAc), R_f_ = 0.29 (hexane/EtOAc 1:1), de > 99% (NMR), [α]^D^_25_ + 40.3 (c 1.0, CHCl_3_). ^1^H NMR (CDCl_3_, 400 MHz): δ 6.80–6.75 (m, 2H, Ar), 6.75–6.71 (m, 1H, Ar), 6.36 (s, 1H, OH), 5.69 (ddt, 1H, *J* = 17.2, 10.2, 7.0 Hz, CH=), 5.06–4.98 (m, 2H, CH_2_=), 4.51 (q, 1H, *J* = 7.3 Hz, CH), 4.20 (d, 1H, *J* = 7.7 Hz, NH), 3.79 (s, 3H, OMe), 2.74–2.67 (m, 1H, CH_2_), 2.61–2.54 (m, 1H, CH_2_), 1.20 (s, 9H, *t*Bu) ppm. ^13^C NMR (CDCl_3_, 101 MHz): δ 146.9, 143.2, 135.0, 128.1, 120.1, 119.6, 117.4, 109.8, 57.3, 56.2, 55.9, 40.4, 22.8 (3C) ppm. Anal. M_r_(C_15_H_23_NO_3_S) = 297.140, calcd: C, 60.58; H, 7.80; N, 4.71; found: C, 60.61; H, 7.92; N, 4.58.

#### 3.3.3. Carboxylation of Ellman’s Phenolic Sulfinamide

Under Ar atmosphere NaH (60% dispersion in mineral oil) (120 mg, 3.0 mmol, 2.0 equiv.) was washed with *n*-hexane (3 × 3 mL) to remove oil. To the washed NaH were added THF (5 mL) and a solution of phenolic sulfonamide (446 mg, 1.5 mmol) in THF (5 mL). After 1 h, when H_2_ evolution ceased, the solvent was evaporated under reduced pressure (15 Torr), and **1r** was dried under vacuum (0.1 mbar) on an oil bath (100 °C) for 3 h, yielding **1r** as a pale yellow solid. Under Ar atmosphere **1r** (341 mg, 1.0 mmol), MesONa (158 mg, 1.0 mmol, 1.0 equiv.), and DMSO (1 mL) were placed into a steel pressure reactor (20 mL) equipped with a magnetic stirring bar. The reactor was sealed under 15 bar of CO_2_ and immersed into an oil bath (100 °C) with stirring for 24 h. The reaction mixture was cooled; the CO_2_ pressure was slowly released (≈10 min). NaHCO_3_ (sat. solution) (2 mL, 2 equiv.) was added to the reaction mixture, and organic impurities were removed by extraction with EtOAc (3 × 5 mL). The aqueous phase was acidified with AcOH (577 μL, 10 equiv.), and the product was extracted with EtOAc (3 × 5 mL). The combined organic extract was washed with NaCl (sat solution) (1 × 5 mL), dried over Na_2_SO_4_ and evaporated to dryness. The residue was purified by column chromatography (EtOAc) to give **3r** (252 mg, 74%) as a pale beige solid. R_f_ = 0.15 (EtOAc), m.p. = 108–110 °C (EtOAc), [α]^D^_25_ + 132.3 (c 1.0, CHCl_3_). ^1^H NMR (DMSO-d_6_, 400 MHz): δ 12.52 (br.s, 1H, COOH), 9.57 (br.s, 1H, OH), 7.69 (d, 1H, *J* = 2.0 Hz, Ar), 7.35 (d, 1H, *J* = 1.9 Hz, Ar), 5.78–5.67 (m, 2H, CH= and NH), 5.02–4.94 (m, 2H, CH_2_=), 4.63 (td, 1H, *J* = 8.6, 5.8 Hz, CH), 3.84 (s, 3H, OMe), 2.50–2.43 (m, 1H, CH_2_), 2.38–2.31 (m, 1H, CH_2_), 1.08 (s, 9H, *t*Bu) ppm. ^13^C NMR (DMSO-d_6_, 101 MHz): δ 167.5, 147.2, 146.6, 135.6, 130.3, 122.3, 121.0, 116.9, 110.4, 55.8, 55.7, 53.1, 40.9, 22.6 (3C) ppm. Anal. M_r_(C_16_H_23_NO_5_S) = 341.130, calcd: C, 56.29; H, 6.79; N, 4.10; found: C, 56.20; H, 6.82; N, 4.08.

## 4. Conclusions

As a result of the current investigation, we have developed a method for gentle, efficient, and homogeneous carboxylation of phenoxides using the Kolbe-Schmitt reaction. Novel fundamental data on the reactivity of alkali metal phenoxides in DMSO and DMF were obtained, including concentration-dependent regioselectivity of the carboxylation. Especially interesting were record values of *para*-regioselectivity on carboxylation of rubidium and cesium salts of phenoxides in DMSO (**SA** was not detected at low concentrations of phenoxide). The use of sodium mesitolate as a promoting additive provides higher chemical yield of a variety of hydroxyaromatic acids, including heterocyclics. Under optimized conditions we achieved nearly quantitative yields of hydroxyaromatic acids with several substrates, confirming the effectiveness and practicality of our method.

Furthermore, we utilized our developed method to record ^13^C NMR spectra of carbonate complexes of substituted phenoxides in DMSO solution for the first time, including the chemical shift in the carboxylate carbon, the least intense signal in the spectra. By studying DOSY spectra of phenoxides alongside calibration standards, we identified molecular associates which are suggested to be responsible for the most rapid carboxylation in solution.

## Figures and Tables

**Table 1:** Carboxylation of PhONa in DMF.

n	PhONa/DMF Ratio, M/M	4HBA, %	SA, %	4HBA&SA, %	4HBA/SA
1	1.0:11.4 (1 M)	34.1	12.5	46.6	2.73
2	1.0:4.0	36.1	18.8	54.9	1.92
3	1.0:3.0	35.1	22.8	57.9	1.54
4	1.0:2.5 (5 M)	34.0	29.0	63.0	1.17
5	1.0:2.0	33.3	32.9	66.2	1.01
6	1.0:1.5	32.4	33.9	66.3	0.96
7	1.0:1.0	21.4	37.8	59.2	0.56
8	3.0:1.0	12.0	33.7	45.7	0.36
9	5.0:1.0	7.2	30.5	37.8	0.24
10	1.0:2.0 ^a^	36.4	47.2	83.6	0.77

15 bar CO_2_; 100 °C; for 3 h; ^a^—for 16 h.

**Table 2:** Chemical shifts ^13^C NMR of CO_2_ and synthesized carbonate complexes.

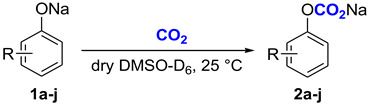		
n	Compounds	Chemical Shift ^13^C, ppm
1	PhOK-CO_2_ in solid state	154 [22]
2	Sat. Solution of CO_2_ in DMF	124 [22]
3	PhOCO_2_[Na-Benzo-15-crown-5]	142 [26]
4	PhOCO_2_Na, **2a**	143
5	MesOCO_2_Na, **2b**	149
6	2,3,5-triMePhOCO_2_Na, **2c**	149
7	4-*t*BuPhOCO_2_Na, **2d**	148
8	2,4-di*t*BuPhOCO_2_Na, **2e**	141
9	2-AllPhOCO_2_Na, **2f**	148
10	2-MeOPhOCO_2_Na, **2g**	137
11	2-ClPhOCO_2_Na, **2h**	125
12	2,5-diClPhOCO_2_Na, **2i**	124
13	2-Cl-5-MePhOCO_2_Na, **2j**	126
14	3-Et-4-ClPhOCO_2_Na, **2k**	140
15	3-MePhOCO_2_Na, **2o**	150

Solutions **2a–j** (100–150 mg) in 1.0 mL of dry DMSO-D_6_ after CO_2_ (10 bar) saturation were subjected to NMR.

**Table 3:** Comparable carboxylation of substituted phenoxides.

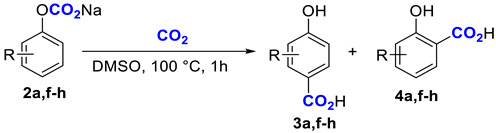				
n	Carbonate	δ ^13^C, ppm	Yield 3&4, %	Ratio 3/4
1	2-AllPhOCO_2_Na, **2f**	148	50.8	93:7
2	PhOCO_2_Na, **2a**	143	40.0	92:8
3	2-MeOPhOCO_2_Na, **2g**	137	37.2	93:7
4	2-ClPhOCO_2_Na, **2h**	125	10.2	94:6

100 °C, 1 h, 15 bar in DMSO.

**Table 4:** Substrate scope for carboxylation.

Compound	Solvent	Additive	Yield 3&4, % ^a^	Ratio 3/4 ^a^
**1a**	DMSO		52(40) ^b,c^	97:3
	DMF		67	54:46
**1c**	DMSO		20	**3** ND
	DMF		46	**3** ND
	DMF	MesONa	64	**3** ND
	DMF ^d^	MesONa	84(76)	**3** ND
**1d**	DMSO		6	
	DMF		74	
	DMF	MesONa	98(95)	
**1e**	DMF		74	
	DMF	MesONa	93(87)	
**1f**	DMSO		54	94:6
	DMSO	MesONa	73(65)	92:8
	DMF		86	66:34
**1g**	DMSO		43	94:6
	DMSO	MesONa	68(55)	90:10
	DMF		73	77:23
**1h**	DMSO		42(30)	96:4
	DMF		36	83:17
**1j**	DMF		27	68:32
**1k**	DMF		75	
	DMF	MesONa	>99(98)	
**1l**	DMF		41	
	DMF ^d^	MesONa	82(70)	
**1m**	DMSO		61	94:6
	DMSO	MesOCs	68(57) ^e^	99:1
	DMF		78	58:42
**1n**	DMSO		55	
	DMSO	MesONa	84(73)	
	DMF		39	
**1o**	DMSO		27	32:68
	DMF		70	1:99
	DMF	MesONa	92(82)	3:97
**1p**	DMSO ^f^		96	
	DMSO ^f^	MesONa	99(96)	
**1q**	DMSO		57	
	DMSO	MesONa	95(84)	
	DMF		34	
**1r ^g^**	DMSO		32	
	DMSO	MesONa	80(74) ^h^	
	DMF		13	

^a^ Determined by NMR; ^b^ Isolated yield in parentheses; ^c^ PhORb; ^d^ 120 °C; ^e^ cesium salt of *o*-cresol; ^f^ 75 °C; ^g^ disodium salt of sulfonamide **1r**; ^h^—40 h; ND—not detected.

## Data Availability

The original contributions presented in this study are included in the article/Supplementary Materials. Further inquiries can be directed to the corresponding author(s).

## Supplementary Materials

The following supporting information can be downloaded at https://www.mdpi.com/article/10.3390/molecules31020239/s1, experimental details; NMR spectra are available in the supporting information file. Table S1. Required volumes of DMSO and PhOM solutions. Photo S1. Left: Small steel pressure reactors (V = 20 mL). Right: A large steel pressure reactor for a multi-vial reaction (V = 700 mL). Photo S2. Concentrated solutions of PhOCs (left) and PhOLi*CO_2_ (right) at 3.0 M concentration in DMSO. Scheme S1. Gas measuring apparatus. Table S2. Required volumes of DMSO and PhONa solutions. Figure S1. Dependence of the logarithm of the diffusion coefficient on the logarithm of the molecular weight at C(PhONa) = 0.036 M. Figure S2. Dependence of the logarithm of the diffusion coefficient on the logarithm of the molecular weight at C(PhONa) = 0.143 M. Figure S3. Dependence of the logarithm of the diffusion coefficient on the logarithm of the molecular weight at C(PhONa) = 0.250 M. Figure S4. Dependence of the logarithm of the diffusion coefficient on the logarithm of the molecular weight at C(PhONa) = 0.357 M. Table S3. Calculation of diffusion coefficients and molecular weights. Refs. [25,26,35,36,37,38,39,40,41,42,43,44,45,46] are cited in the Supplementary Materials.
