# Supplementary material for: Synthesis of Hydroxyaromatic Carboxylic Acids via Homogeneous Kolbe-Schmitt Carboxylation of Phenoxides
Source: Molecules. 2026 Jan 10;31(2):239. doi: 10.3390/molecules31020239 (PMC12843966; doi:10.3390/molecules31020239)

Synthesis of Hydroxyaromatic Carboxylic Acids via Homogeneous Kolbe-Schmitt Carboxylation of Phenoxides

Dmitriy A. Merzliakov [1,2], Michael S. Alexeev [1], Maxim A. Topchiy [1], Dmitry G. Yakhvarov [3], Nikolai Yu. Kuznetsov [1,*], Anton L. Maximov [1,2] and Irina P. Beletskaya [1,2]

[1] A.V. Topchiev Institute of Petrochemical Synthesis, Russian Academy of Sciences, Leninsky Prospect 29, 119991 Moscow, Russia; mda@ips.ac.ru (D.A.M.); alekseev@ips.ac.ru (M.S.A.); maxtopchiy@ips.ac.ru (M.A.T.); max@ips.ac.ru (A.L.M.); beletska@org.chem.msu.ru (I.P.B.)
[2] Faculty of Chemistry, Lomonosov Moscow State University, Leninskie Gory 1, 119991 Moscow, Russia
[3] Federal Research Center Kazan Scientific Center of Russian Academy of Sciences, Lobachevskogo st. 2/31, 420111 Kazan, Russia; yakhvar@iopc.ru
* Correspondence: nkuznff@ips.ac.ru

Supporting Information

Table of contents

General 2
Reagents and solvents 2
Dry DMSO and dry DMF preparation 3
  Activation of 4Å molecular sieves 3
  Drying of DMSO 3
  Drying of DMSO-d6 3
  Drying of DMF 3
Procedures of synthesis of substances 4
  PhORb 4
  PhOCs 4
  MesOCs (cesium 2,4,6-trimethylphenoxide) 4
  Synthesis of sodium phenoxides 4
Carboxylation experimental procedures 5
  Standard procedure for the carboxylation of sodium phenoxide in DMSO 5
  Standard procedure for the carboxylation of sodium phenoxide in DMF 5
  Procedure for determining of concentration dependencies for PhORb and PhOCs solutions 5
  Procedure for determining of concentration dependences for PhOLi solutions 6
Photos of steel pressure reactors and concentrated phenoxide solutions 6
  Photo S1. *Left: Small steel pressure reactors (V = 20 mL).* 7
Gasometric determination of the stability of carbonate complexes of phenoxides in DMSO solution 7
Determination of the molecular weight of the PhONa carbonate complex by DOSY 8
Registration of NMR spectra of the “carbonate complex” 11
NMR spectra of hydroxyaromatic carboxylic acids 14
Preparation of a chiral Ellman’s sulfonamide with a phenolic group 18
References 20
Copies of NMR spectra 21

General. All manipulations with air- and moisture-sensitive compounds were carried out under inert atmosphere of argon N5.5 (99.9995%) by Schlenk technique.[1] Carboxylation of metal phenoxides was carried out in an atmosphere of CO_2 N4.0 (99.99%). NMR spectra were recorded on Bruker Avance III HD 400 instrument. Chemical shifts in the ^{1}H NMR spectra are given relative to the residual signals of H-solvents: $CHCl_3$ 7.26 ppm and DMSO 2.50 ppm; in the ^{13}C NMR spectra, relative to the signals of deuterated solvents: $CDCl_3$ 77.16 ppm and DMSO-D_6 39.52 ppm. Chromatography was performed on a Sintecon HPLC 10.400 (China) with a built-in degasser, autosampler, thermostat and Diode Array Detector (DAD). Chromatograms were processed using Empower software (version 3). Samples were injected onto a Hypersil ODS column (5 µm, 250 x 4.6 mm). HPLC conditions for the separation of phenol, salicylic acid and 4-hydroxybenzoic acid were similar to those used in the previous work.[2] Column chromatography was performed using silica gel 60–230 mesh (Merck). Thin-layer chromatography was performed on aluminum TLC plates with silica gel 60F UV_{254} (GLR Innovations). Melting points were measured on a Cole-Parmer SMP-30 capillary melting point apparatus (uncorrected). Optical rotation was measured using a Perkin Elmer 341 polarimeter. Elemental analyses were performed in the laboratory of microanalysis of IPS RAS.

Reagents and solvents. All reagents and solvents, unless otherwise stated, were commercially available and used without any treatment. THF (99.5%, Component-Reaktiv) was freshly distilled over sodium benzophenone ketyl radical. DCM (99.8%, Component-Reaktiv) was freshly distilled over P_2O_5 (98%, Component-Reaktiv). Acetonitrile (99.9%, Carlo Erba), 2-propanol (99.8%, Scharlau), and methanol (99.9%, Carlo Erba) were HPLC grade. Water was obtained using a Millipore AFS 8D Water Purification System. All HPLC solvents were filtered through a 0.5 µm filter before use. Phenol (99.5%, Riedel-de Haën), (*S*)-2-methyl-2-propanesulfinamide (99%, AK Scientific), titanium(IV) isopropoxide (97%, Aldrich), triphenylphosphine oxide (97%), 1,1′-bis(diphenylphosphino)ferrocene (99%, DalChem), polysterene analytical standard for gel permeation chromatography (1320 g/mol), potassium hydroxide (86%, Component-Reaktiv), rubidium hydroxide hydrate (99%, Alfa Aesar), cesium hydroxide monohydrate (95%, Angene), NaH (60% dispersion in oil, DalChem), CaH_2 (93%), $NaHCO_3$ (99%, BSC Jsc.), Na_2SO_4 (99%, Component-Reaktiv), $MgSO_4$ (98%), K_2CO_3 (98%, Component-Reaktiv), NaCl (99.8%, Component-Reaktiv), formic acid (FA, 99%, Carlo Erba), hydrochloric acid (35-38%, Component-Reaktiv), NH_3 25% solution (Sigma-Tek LLC.), AcOH (99.8%, Component-Reaktiv), ethyl acetate (99.7%, Component-Reaktiv), chloroform (99.85%, Component-Reaktiv), *n*-hexane (98.0%, Component-Reaktiv), Et_3N (98.6%, Component-Reaktiv), toluene (99%, Component-Reaktiv) were used as received. All commercially available phenols were dried by distillation under reduced pressure before the synthesis of metal phenoxides. Potassium *tert*-butoxide (98%, Acros Organics) was sublimated at 200 °C (0.5 Torr) prior to use. NaBr was dried at 220 °C under vacuum (0.1 mbar) for 2 h. LiOPh, MesOH (2,4,6-trimethylphenol), MesONa (sodium 2,4,6-trimethylphenoxide), and NaOPh were synthesized as described previously.[2] $(PPh_3)_3CuCl$*MeCN[3] and TABDABCO[4] were synthesized as described previously.

Dry DMSO and dry DMF preparation

Activation of 4Å molecular sieves

1 kg of MS 4Å (Aldrich) in a 2-L flask was heated on a Wood's alloy bath at 300 °C under vacuum (0.1 mbar), shaking the sieves occasionally. Drying continued for several hours until water condensation ceased to form in the liquid N_2 trap. After cooling to rt, the flask containing MS 4Å was flushed with Ar.

Drying of DMSO[5]

Under Ar atmosphere a 2-L, two-necked flask, fitted with a magnetic stirring bar and attached to an oil bubbler via a stopcock, was charged with 1.5 L of commercially available DMSO (PanReac, water content 0.2%) and grinded CaH_2 (20 g). This suspension was stirred on an oil bath at 75 °C for 24 h with H_2 evolution. DMSO was distilled over this suspension of CaH_2 under reduced pressure (15 Torr) using an oil bath (110 °C) via a distilling link yielding 1.45 L of DMSO (water content 150 ppm by coulometric Karl Fischer titration, one sample $m_{sampleN0}$ = 2.5741 g). Under Ar atmosphere activated MS 4Å (175 g, 12% w/v DMSO) were transferred to the distilled DMSO. After two weeks, the water content was analyzed by coulometric Karl Fischer titration on 3 DMSO samples ($m_{sampleN1}$ = 3.2474 g, $m_{sampleN2}$ = 3.0458 g, $m_{sampleN3}$ = 3.1761 g, $[water]_{sampleN1}$ = 14.0 ppm, $[water]_{sampleN2}$ = 13.6 ppm, $[water]_{sampleN3}$ = 14.4 ppm), giving an average value of 14 ppm. DMSO was stored over MS 4Å in the flask with silicone-greased glass stoppers to ensure a good seal throughout chemical experiments.

Drying of DMSO-d_6

Under Ar atmosphere a 100 mL Schlenk storage tube with high vacuum valve was charged with activated MS 4Å (5 g, 10% w/v DMSO) and 50 mL of commercially available DMSO-d_6 (Solvex-D, D 99.8%, water content 0.01%). After two weeks, the water content was analyzed by coulometric Karl Fischer titration on 3 DMSO-d_6 samples ($m_{sampleN1}$ = 0.4633 g, $m_{sampleN2}$ = 0.7980 g, $m_{sampleN3}$ = 0.6951 g, $[water]_{sampleN1}$ = 4.0 ppm, $[water]_{sampleN2}$ = 6.1 ppm, $[water]_{sampleN3}$ = 8.3 ppm), which gave an average value of 6 ppm. DMSO-d_6 was stored over MS 4Å in the Schlenk storage tube with silicone greased valve cock to ensure a good seal throughout chemical experiments.

Drying of DMF

DMF was dried as described above using activated MS 4Å (11 g, 14% w/v DMF) and 80 mL of DMF (Acros Organics, extra dry, water content <50 ppm). After two weeks, the water content was analyzed by coulometric Karl Fischer titration on 3 DMF samples ($m_{sampleN1}$ = 9.6678 g, $m_{sampleN2}$ = 8.7450 g, $m_{sampleN3}$ = 8.9341 g, $[water]_{sampleN1}$ = 4.2 ppm, $[water]_{sampleN2}$ = 8.0 ppm, $[water]_{sampleN3}$ = 5.9 ppm), which gave an average value of 6 ppm. Similarly, storage over MS 4Å was performed throughout chemical experiments.

Procedures of synthesis of substances

PhORb

Phenol (9.4 g, 100.0 mmol) was added to a solution of RbOH*H_2O (12.0 g, 100.0 mmol) in MeOH (30 mL); the solvent was distilled under reduced pressure (15 Torr) on a water bath (30 °C). Water was removed by azeotropic distillation with toluene using a Dean-Stark apparatus. Toluene was evaporated under reduced pressure, after that rubidium phenoxide was dried under vacuum (0.1 mbar) on an oil bath (250 °C) for 10 h, yielding a beige solid. The NMR spectrum of the rubidium salt is similar to that of the sodium salt.

PhOCs

Phenol (9.4 g, 100.0 mmol) was stirred with a suspension of CsOH*H_2O (16.8 g, 100.0 mmol) in toluene (30 mL). Water was removed by azeotropic distillation with toluene using a Dean-Stark apparatus. Toluene was evaporated under reduced pressure, after that cesium phenoxide was dried under vacuum (0.1 mbar) on an oil bath (250 °C) for 10 h, yielding a pink solid. The NMR spectrum of the cesium salt is similar to that of the sodium salt.

MesOCs (cesium 2,4,6-trimethylphenoxide)

2,4,6-Trimethylphenol (5.448 g, 40.0 mmol) and CsOH*H_2O (6.717 g, 40.0 mmol) were dissolved in MeOH (20 mL) with a slight exoterm. MeOH was evaporated under reduced pressure. Water was removed by azeotropic distillation with toluene using a Dean-Stark apparatus. Toluene was evaporated under reduced pressure; after that the residue was dried under vacuum (0.1 mbar) on an oil bath (180 °C) for 10 h, yielding a dry brown glass-like mass. To grind this mass, the flask was immersed in liquid N_2, yielding a nice beige powder. The NMR spectrum of the cesium salt is similar to that of the sodium salt.

Synthesis of sodium phenoxides

Variation A (1f, 1g, 1m, 1n, 1p)

Sodium (569 mg, 24.8 mmol) was dissolved in dry MeOH (10 mL) from 0 to 25 °C. Phenol (25.0 mmol) was dissolved in a minimal volume of MeOH, then a MeONa solution was added. The solvent was distilled under reduced pressure (15 Torr) on a water bath (30 °C), then solid phenoxide salt was crushed into a powder under Ar. This powder was dried under vacuum (0.1 mbar) on an oil bath (200 °C) for 10 h.

Variation B (1c-1e, 1h-1l, 1o, 1q)

Under Ar atmosphere NaH (60% dispersion in mineral oil) (280 mg, 7.0 mmol) was washed three times with hexane (5 mL) to remove the oil. dry THF or DCM (10 mL) was added to the washed NaH powder, and phenol (7.0 mmol) was added portion wise. After 1 h, when H_2 evolution was complete, the solvents were evaporated under reduced pressure (15 Torr), and sodium phenoxide was dried under vacuum (0.1 mbar) on an oil bath (200 °C) for 10 h.

Carboxylation experimental procedures

Standard procedure for the carboxylation of sodium phenoxide in DMSO

Under Ar atmosphere sodium phenoxide (1.0 mmol) was poured as a solid from a bulk dry salt into a small glass vessel (3 mL) equipped with a magnetic stirrer bar. Anhydrous DMSO (1 mL) was added, and the vessel was placed into a steel pressure reactor (V = 20 mL, Photo S1). The reactor was pressurized under 15 bar of CO_2 and placed into an oil bath (100 °C) with stirring for 16 h. After the completion of the reaction, the reactor was cooled, and CO_2 pressure was slowly released (≈ 10 min).

For NMR analysis, the crude reaction mixture (1 mmol) was quenched with formic acid (189 µL, 5.0 mmol, 5.0 equiv.), and a 75 µl aliquot was diluted in DMSO-d_6 (0.5 mL).

For separation of the product, $NaHCO_3$ (sat. solution) (2 mL, 2.0 equiv.) was added to the reaction mixture, and organic impurities were removed by extraction with EtOAc (3 x 5 mL). The aqueous layer was acidified to pH = 1 with conc. HCl, and the product was extracted with EtOAc (3 x 5 mL). The combined organic extract was washed with NaCl (sat solution) (1 x 5 mL), dried over Na_2SO_4 and evaporated to dryness. The pure product was obtained by recrystallization or column chromatography.

Standard procedure for the carboxylation of sodium phenoxide in DMF

Under Ar atmosphere sodium phenoxide (1.0 mmol) was poured as a solid from a bulk dry salt into a small glass vessel (3 mL) equipped with a magnetic stirrer bar. Anhydrous DMF (155 µL, 2.0 mmol, 2.0 equiv.) was added, and the vessel was placed into a steel pressure reactor (V = 20 mL, Photo S1). The reactor was pressurized under 15 bar of CO_2 and placed into an oil bath (100 °C) with stirring for 16 h. After the completion of the reaction, the reactor was cooled, and CO_2 pressure was slowly released (≈ 10 min).

For NMR analysis, if the sample is heterogeneous, it can be diluted with DMF or DMSO (1 mL), and after quenching with formic acid (189 µL, 5.0 mmol, 5.0 equiv.) a 75 µl aliquot was diluted in DMSO-d_6 (0.5 ml).

For separation of the product, $NaHCO_3$ (sat. solution) (2 mL, 2.0 equiv.) and water (5 mL) were added to the reaction mixture, and organic impurities were removed by extraction with EtOAc (3 x 5 mL). The aqueous layer was acidified to pH = 1 with conc. HCl, and the product was extracted with EtOAc (3 x 5 mL). The combined organic extracts were washed with NaCl (sat solution) (1 x 5 mL), water (1 x 5 mL), dried over Na_2SO_4, and evaporated to dryness. The pure product was obtained by recrystallization or column chromatography.

Procedure for determining concentration dependencies for PhORb and PhOCs solutions

Under Ar atmosphere a concentrated 3.0 M solution of PhOM (M = Rb or Cs) in DMSO was prepared by heating on an oil bath (100 °C). For example, under Ar atmosphere RbOPh (1.786 g, 10.0 mmol) and DMSO (2.0 mL) were placed into a Schlenk vessel (10 mL) equipped with a magnetic stirrer bar, and the

mixture was stirred at 100 °C until the salt was completely dissolved (Photo S2). An aliquot (100 µL) of this solution was transferred to a 100 mL volumetric flask and diluted with MeCN/H_2O (1:1, v/v) to 100.0 mL. The resulted concentration was analyzed by HPLC using a phenol calibration curve. HPLC analysis yielded a 3.2 M concentration, so an additional amount of DMSO (0.21 mL) was added to prepare a 3.0 M solution. After adding the required amount of DMSO, the RbOPh solution concentration was 3.0 M according to HPLC.

A concentrated 3.0 M PhOM solution and dry DMSO were added to the glass vials (V = 5 mL) equipped with magnetic stirring bars in appropriate volumes to achieve the desired concentration (see Table S1). The prepared vials were placed into a steel pressure reactor (V = 700 mL, Photo S1), which was flashed with CO_2, sealed, and pressurized under 15 bar of CO_2. The reactor was heated on an oil bath at 100 °C (controlled with an internal thermocouple) with stirring overnight (16 h). After cooling to rt, the CO_2 pressure was released, and the reaction was quenched with formic acid (5.0 equiv.). The reaction mixtures were analyzed using previously described HPLC procedure.[2]

Table S1. Required volumes of DMSO and PhOM solutions.

N	Resulting concentration of PhOM, M	Volume of 3.0 M PhOK, µl	Volume of DMSO, µl
1	3.0	1000	0
2	2.0	800	400
3	1.5	400	800
4	0.75	250	750
5	0.5	160	800
6	0.25	80	880
7	0.125	40	920

Procedure for determining of concentration dependences for PhOLi solutions

Since PhOLi is poorly soluble even in hot DMSO (100 °C), it was dissolved as the carbonate complex PhOLi*CO_2. Under Ar atmosphere PhOLi (600.0 mg, 6.0 mmol) and DMSO (1.6 mL) were added to a Duran tube (V = 10 mL) equipped with a magnetic stirrer bar. The opened tube was placed into steel pressure reactor and stirred under CO_2 pressure (15 bar) for 1 h at rt. The CO_2 pressure was slowly released (≈ 10 min), and the insoluble parts of the lithium phenoxide were crushed with a glass rod under CO_2. The resulting suspension was again maintained under CO_2 pressure for 1 h, yielding a concentrated 3.0 M solution (Photo S2). Other experimental details were identical to the procedure for the preparation of solution of Rb and Cs phenoxide salts.

Photos of steel pressure reactors and concentrated phenoxide solutions

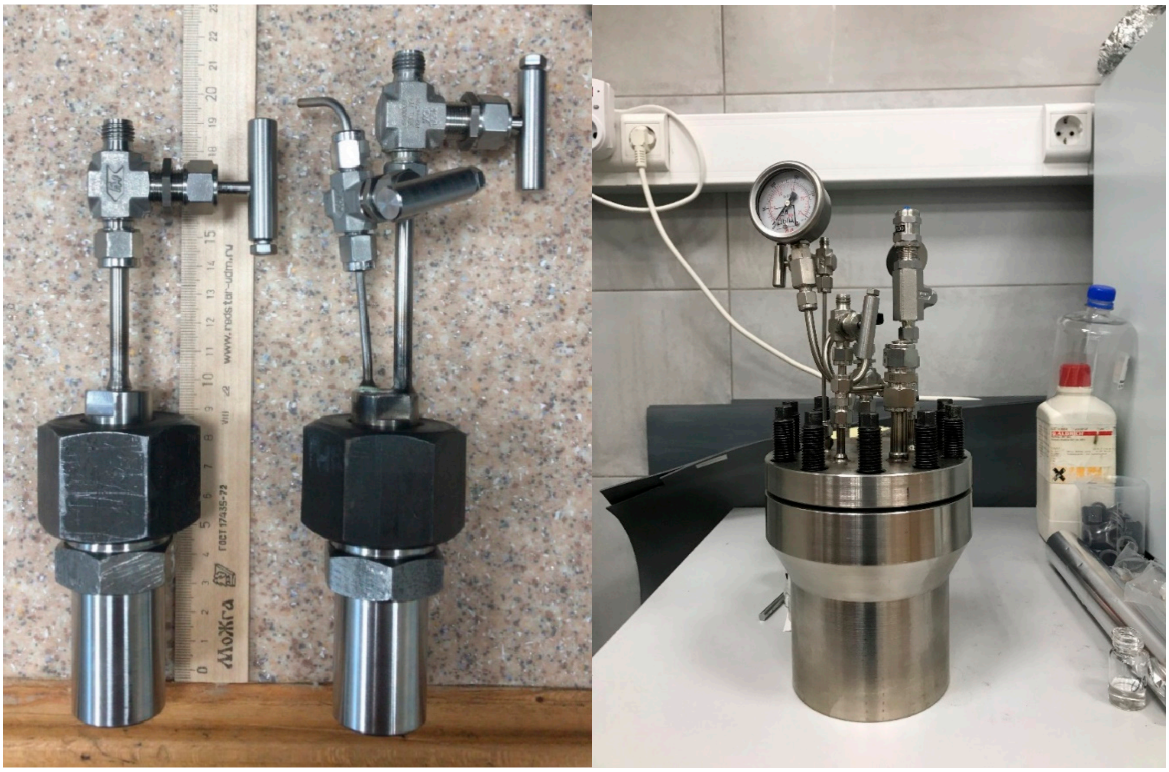

Photo S1. *Left: Small steel pressure reactors (V = 20 mL).* On the left is an usual reactor with a valve for feeding CO_2. On the right is a reactor with a sampling valve for withdrawing aliquots in a kinetic experiment; it has a long capillary immersed into the stirred solution. *Right: A large steel pressure reactor for a multi-vial reaction (V = 700 mL).* The steel pressure reactor is equipped with a pressure gauge (250 bar), a sampling valve, a safety valve (200 bar), a CO_2 feeding valve, a solid loading valve (d = 10 mm), and a long sealed metal capillary (d = 3 mm, l = 160 mm) for temperature measurement.

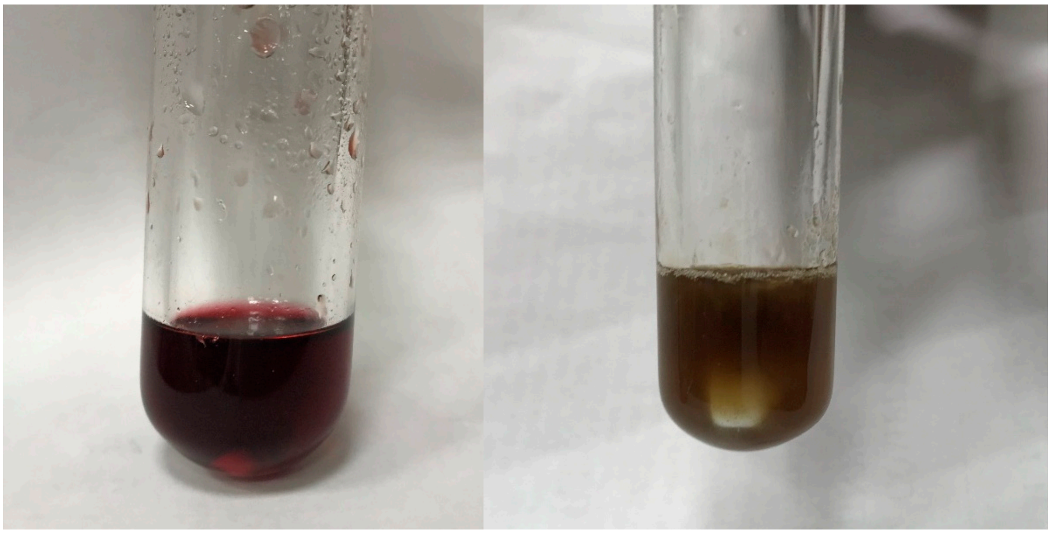

Photo S2. Concentrated solutions of PhOCs (left) and PhOLi*CO_2 (right) at 3.0 M concentration in DMSO.

Gasometric determination of the stability of carbonate complexes of phenoxides in DMSO solution

PhOKCO_2 stability determination.* A 1.0 M solution of potassium phenoxide in DMSO (2.0 mL) was placed into an argon-purged steel pressure reactor (20 mL). The solution was stirred for 15 min under CO_2

pressure (15 bar). After releasing the excess CO_2 pressure, 1.0 mL of the resulting PhOK*CO_2 solution in DMSO was placed in a flask with an outlet. The flask was connected via silicon tube to a gas measuring jacketed gasometer tube with water thermostating of the evolved gas. The flask with the DMSO solution was sealed, and the pressure in the flask and gas measuring tube was allowed to stabilize. Then the flask with the resulting solution was gradually heated in an oil bath, while recording the volume of CO_2 released from the flask into the gas measuring apparatus, monitoring the temperature change per degree Celsius. Upon heating, PhOK*CO_2 decomposes into PhOK and CO_2. Heating was carried out for 38 min, with an initial temperature of 27 °C and a final one of 130°C, with an average heating rate of 2.7 °C/min.

MesONaCO_2 stability determination.* 1.0 ml of a 1.0 M MesONa solution in DMSO was placed into an argon-purged steel pressure reactor (20 mL) and stirred for 15 min under CO_2 pressure (15 bar). Then, 0.8 mL of the MesONa*CO_2 solution was placed in a flask with an outlet. The flask was connected, and measurements were performed as described above. Heating was carried out for 45 min, with an initial temperature of 27 °C and a final one of 123 °C, with an average heating rate of 2.1 °C/min.

Determination of CO_2 thermal expansion. To evaluate the contribution of thermal expansion and evolution of CO_2 dissolved in DMSO and to distinguish it from the evolved CO_2 formed during the decomposition of PhOK*CO_2 and MesONa*CO_2, the gasometric measurements were performed using a CO_2 solution in DMSO. For this, 3 mL of DMSO was stirred in an argon-purged steel pressure reactor (20 mL) for 15 min under CO_2 pressure (15 bar). After releasing the excess of dissolved CO_2 (10 min.) with stirring, 1.0 mL of DMSO with dissolved CO_2 was placed in a flask connected to a gasometer, and measurements were performed as described above. Heating was carried out for 27 min, the initial temperature was 26 °C, and a final one was 120 °C, above which no more gas evolution was observed.

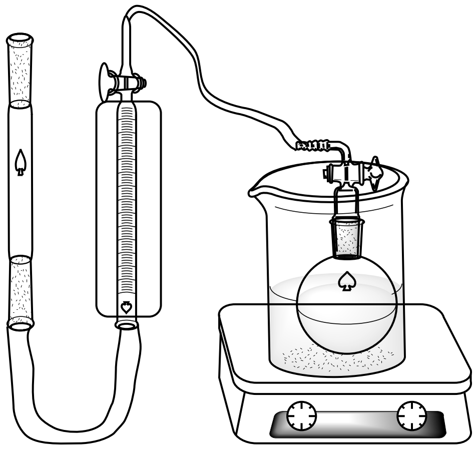

Scheme S1. Gas measuring apparatus.

Determination of the molecular weight of the PhONa carbonate complex by DOSY

A stock solution of 4 standards was prepared in dry DMSO-d_6: DMSO-d_5 (M_r = 83 g/mol), triphenylphosphine oxide (Ph_3PO), 1,1′-bis(diphenylphosphino)ferrocene (dppf), and polystyrene with an average molecular weight of 1320 g/mol. A 0.5 M PhONa*CO_2 solution was prepared by mixing PhONa

(165 mg, 1.42 mmol) with DMSO-d_6(2.7 mL) in a steel pressure reactor (20 mL) under CO_2 pressure (10 bar) for 1 h. The obtained homogeneous PhONa*CO_2 solution was transferred into 4 argon-purged NMR tubes in the following volumes: 0.1, 0.4, 0.7, and 1.0 mL. Then DMSO-d_6 (0.9, 0.6, 0.3, and 0.0 ml, respectively) and the standard mixture solution (0.4 ml to each tube) were added. The NMR tubes were placed in a long steel pressure reactor and kept under CO_2 (10 bar) for 30 min. All spectra were acquired in 5 mm NMR tubes. DOSY experiments were recorded using the «ledbpgp2s» pulse sequence (stimulated echo and longitudinal eddy current delay with bipolar gradient pulses). DOSY transformation was performed in MestReNova using the «peak fit» method. Diffusion coefficients are reported in cm²/s; molecular weights are given in g/mol.

Table S2. Required volumes of DMSO and PhONa solutions.

Run	V (0.5M sol. PhONa), ml	V (DMSO-d_6), ml	V (sol. of standarts), ml	V sum, ml	C (PhONa), M
1	0.1	0.9	0.4	1.4	0.036
2	0.4	0.6	0.4	1.4	0.143
3	0.7	0.3	0.4	1.4	0.250
4	1.0	0.0	0.4	1.4	0.357

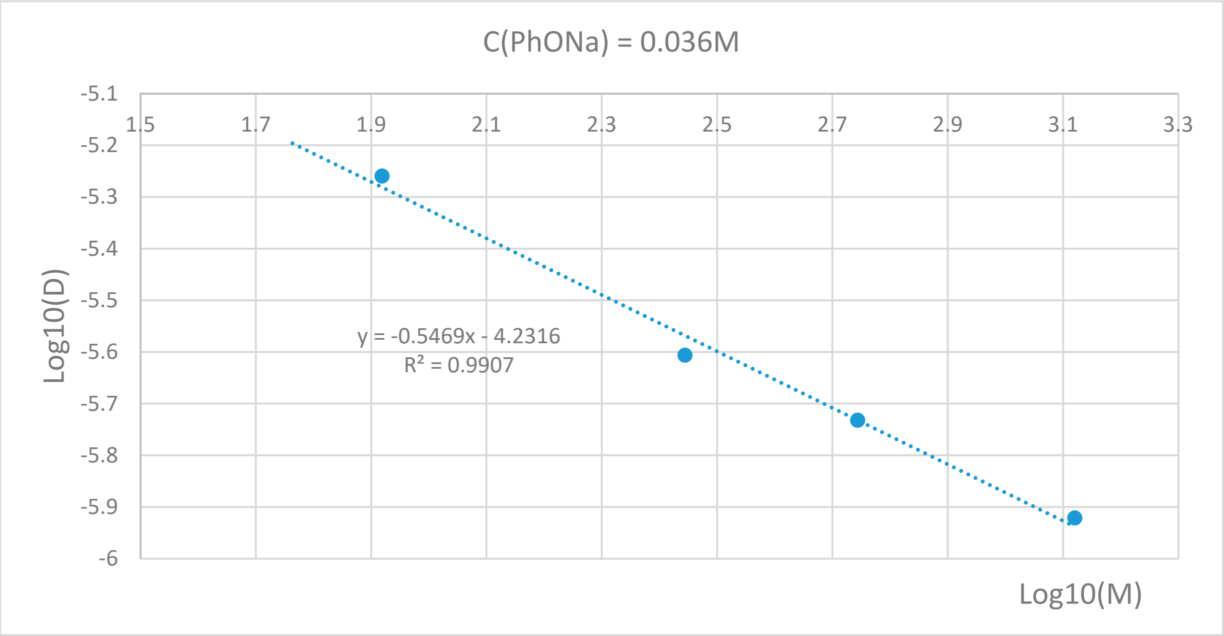

Figure S2. Dependence of the logarithm of the diffusion coefficient on the logarithm of the molecular weight at C(PhONa) = 0.036M.

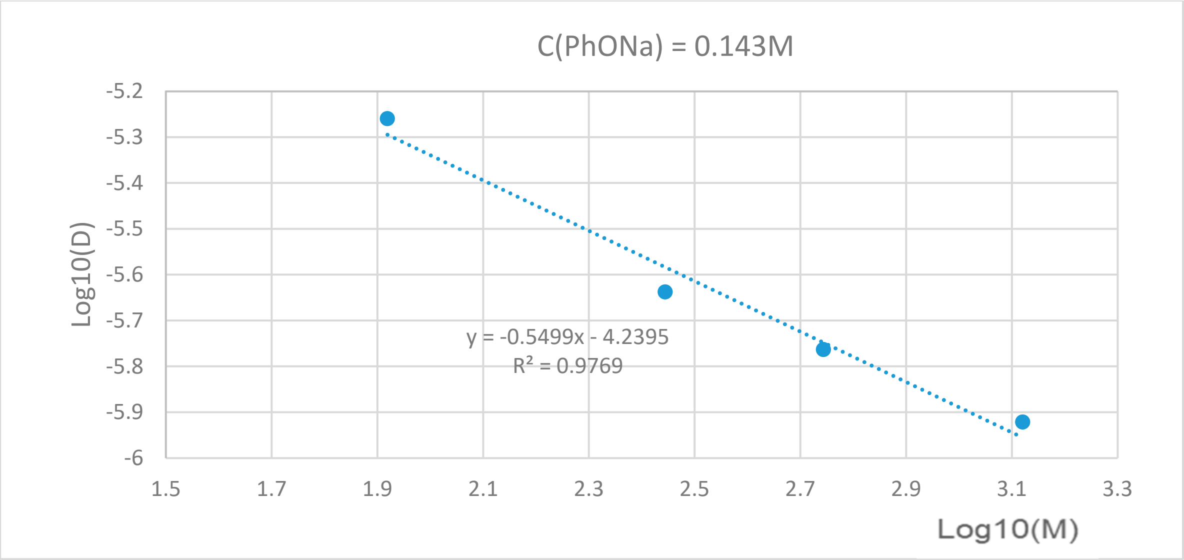

Figure S3. Dependence of the logarithm of the diffusion coefficient on the logarithm of the molecular weight at C(PhONa) = 0.143M.

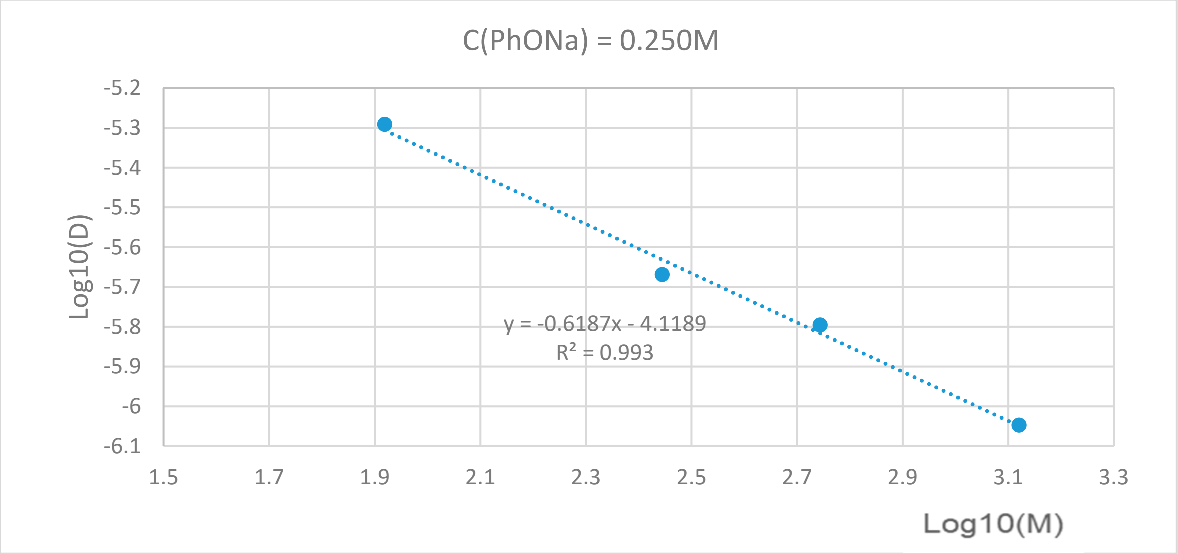

Figure S4. Dependence of the logarithm of the diffusion coefficient on the logarithm of the molecular weight at C(PhONa) = 0.250M.

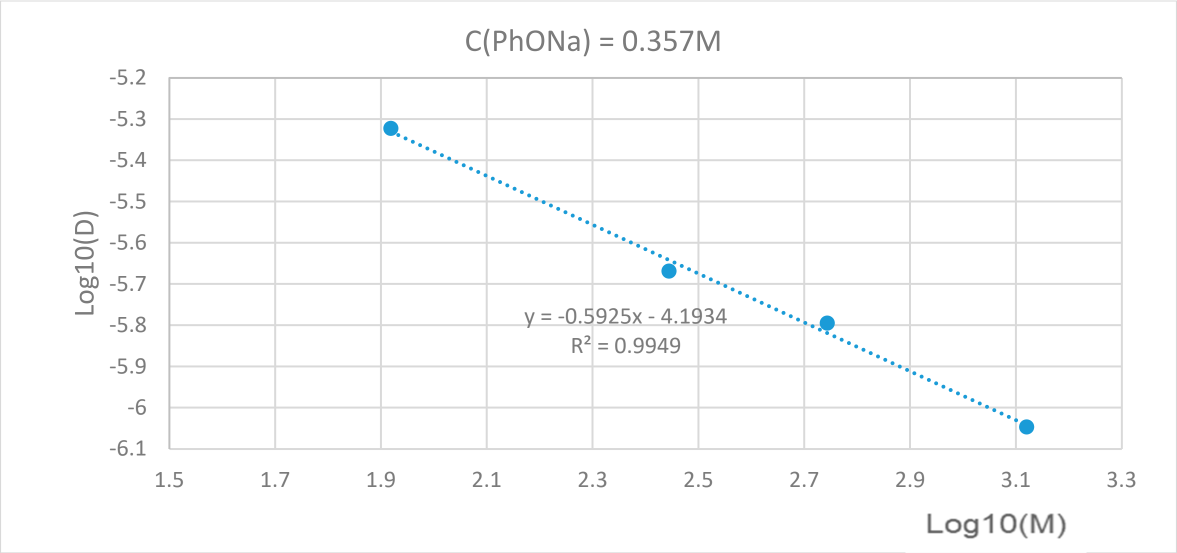

Figure S4. Dependence of the logarithm of the diffusion coefficient on the logarithm of the molecular weight at C(PhONa) = 0.357M.

Table S3. Calculation of diffusion coefficients and molecular weights.

	C (PhONa), M							
	0.036				0.143			
	D, 10^{-6}cm²/s	LOG_{10}(D)	M, g/mol	LOG_{10}(M)	D, 10^{-6}cm²/s	LOG_{10}(D)	M, g/mol	LOG_{10}(M)
DMSO-d_5	5.500	-5.260	83.0	1.919	5.500	-5.260	83.0	1.919
Ph_3PO	2.475	-5.606	278.3	2.444	2.304	-5.638	278.3	2.444
dppf	1.852	-5.732	554.4	2.744	1.724	-5.763	554.4	2.744
PS	1.198	-5.921	1320.0	3.121	1.199	-5.921	1320.0	3.121
	D, 10^{-6}cm²/s	LOG_{10}(D)	M FOUND, g/mol	LOG_{10}(M) FOUND	D, 10^{-6}cm²/s	LOG_{10}(D)	M FOUND, g/mol	LOG_{10}(M) FOUND
PhONa*CO_2	3.310	-5.480	204.5	2.311	2.862	-5.543	234.9	2.371
C (PhONa), M								
	0.250				0.357			
	D, 10^{-6}cm²/s	LOG10(D)	M, g/mol	LOG10(M)	D, 10^{-6}cm²/s	LOG10(D)	M, g/mol	LOG10(M)
DMSO-d_5	5.113	-5.291	83.0	1.919	4.755	-5.323	83.0	1.919
Ph_3PO	2.142	-5.669	278.3	2.444	1.992	-5.701	278.3	2.444
dppf	1.601	-5.796	554.4	2.744	1.603	-5.795	554.4	2.744
PS	0.897	-6.047	1320.0	3.121	0.897	-6.047	1320.0	3.121
	D, 10^{-6}cm²/s	LOG_{10}(D)	M FOUND, g/mol	LOG_{10}(M) FOUND	D, 10^{-6}cm²/s	LOG_{10}(D)	M FOUND, g/mol	LOG_{10}(M) FOUND
PhONa*CO_2	2.475	-5.606	253.6	2.404	2.142	-5.669	309.6	2.491

Registration of NMR spectra of the "carbonate complex"

Sodium phenoxide (100 mg) was suspended in anhydrous DMSO-d_6 (0.5 mL) in glass vial (V = 5 mL). This mixture was transferred into a steel pressure reactor and maintained under 15 bar CO_2 for 1 h until

complete dissolution. After depressurizing of the reactor, the solution was transferred using a syringe into a NMR tube, and this tube was again maintained in steel pressure reactor under 15 bar of CO_2 for 10 min. After depressurizing of the reactor, ^{1}H and ^{13}C NMR spectra were recorded.

Sodium phenoxide * CO_2 (2a)

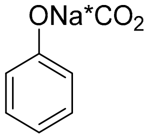

^{1}H NMR (DMSO-d$_6$, 400 MHz): δ 7.08 (t, 2H, *J* = 7.7 Hz, Ar), 6.76 (d, 2H, *J* = 7.9 Hz, Ar), 6.67 (t, 1H, *J* = 7.2 Hz, Ar) ppm. ^{13}C NMR (DMSO-d$_6$, 101 MHz): δ 159.7, **143.1**, 128.5 (2C), 120.0 (2C), 117.8 ppm.

Sodium 2,4,6-trimethylphenoxide * CO_2 (2b)

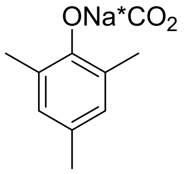

^{1}H NMR (DMSO-d$_6$, 400 MHz): δ 6.69 (s, 2H, Ar), 2.15 (s, 3H, Me), 2.08 (s, 6H, 2Me) ppm. ^{13}C NMR (DMSO-d$_6$, 101 MHz): δ 150.6, **149.1**, 128.8, 128.3 (2C), 127.4 (2C), 20.3, 16.6 (2C) ppm.

Sodium 2,3,5-trimethylphenoxide * CO_2 (2c)

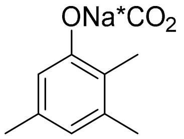

^{1}H NMR (DMSO-d$_6$, 400 MHz): δ 6.56 (s, 1H, Ar), 6.55 (s, 1H, Ar), 2.16 (s, 6H, 2Me), 2.01 (s, 3H, Me) ppm. ^{13}C NMR (DMSO-d$_6$, 101 MHz): δ 154.3, **148.6**, 136.2, 133.9, 124.4, 123.6, 119.6, 20.8, 19.9, 12.1 ppm.

Sodium 4-(*tert*-butyl)phenoxide * CO_2 (2d)

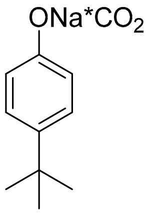

^{1}H NMR (DMSO-d$_6$, 400 MHz): δ 7.16 (d, 2H, *J* = 8.2 Hz, Ar), 6.79 (d, 2H, *J* = 8.2 Hz, Ar), 1.26 (s, 3H, *t*Bu) ppm. ^{13}C NMR (DMSO-d$_6$, 101 MHz): δ 155.2, **148.1**, 141.6, 125.1 (2C), 119.9 (2C), 33.7, 31.6 (3C) ppm.

Sodium 2,4-di-*tert*-butylphenoxide * CO_2 (2e)

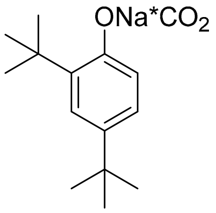

^{1}H NMR (DMSO-d$_6$, 400 MHz): δ 7.15 (s, 1H, Ar), 7.01 (d, 1H, *J* = 8.6 Hz, Ar), 6.77 (d, 1H, *J* = 8.3 Hz, Ar), 1.34 (s, 3H, *t*Bu), 1.25 (s, 3H, *t*Bu) ppm. ^{13}C NMR (DMSO-d$_6$, 101 MHz): δ 153.2, **142.5**, 137.3, 122.6 (2C), 121.9 (2C), 34.4, 33.9, 31.6 (3C), 30.0 (3C) ppm.

Sodium 2-allylphenoxide * CO_2 (2f)

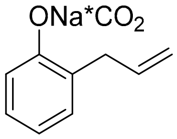

^{1}H NMR (DMSO-d$_6$, 400 MHz): δ 7.03 (t, 2H, *J* = 8.1 Hz, Ar), 6.89 (d, 1H, *J* = 7.6 Hz, Ar), 6.77 (t, 1H, *J* = 7.2 Hz, Ar), 6.01 (ddt, 1H, *J* = 16.9, 10.1, 6.8 Hz, CH=), 5.11-5.00 (m, 2H, CH_2=), 3.35 (d, 2H, *J* = 6.7 Hz, CH_2) ppm. ^{13}C NMR (DMSO-d$_6$, 101 MHz): δ 156.2, **148.3**, 138.0, 130.6, 128.8, 126.6, 121.2, 119.6, 115.2, 34.5 ppm.

Sodium 2-methoxyphenoxide * CO_2 (2g)

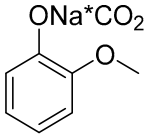

^{1}H NMR (DMSO-d$_6$, 400 MHz): δ 6.71 (d, 1H, *J* = 7.6 Hz, Ar), 6.64 (t, 1H, *J* = 7.5 Hz, Ar), 6.58 (d, 1H, *J* = 6.6 Hz, Ar), 6.37 (t, 1H, *J* = 7.0 Hz, Ar), 3.67 (s, 3H, OMe) ppm. ^{13}C NMR (DMSO-d$_6$, 101 MHz): δ 155.6, 151.0, **136.6**, 121.2, 119.2, 112.8, 110.5, 54.9 ppm.

Sodium 2-chlorophenoxide * CO_2 (2h)

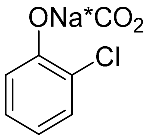

^{1}H NMR (DMSO-d$_6$, 400 MHz): δ 7.03 (d, 1H, *J* = 7.6 Hz, Ar), 6.81 (t, 1H, *J* = 7.6 Hz, Ar), 6.52 (d, 1H, *J* = 8.1 Hz, Ar), 6.10 (t, 1H, *J* = 7.3 Hz, Ar) ppm. ^{13}C NMR (DMSO-d$_6$, 101 MHz): δ 164.2, 128.5, 127.5, **125.0**, 122.2, 119.9, 110.0 ppm.

Sodium 2,5-dichlorophenoxide * CO_2 (2i)

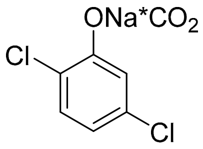

^{1}H NMR (DMSO-d$_6$, 400 MHz): δ 7.07 (d, 1H, *J* = 8.4 Hz, Ar), 6.60 (d, 1H, *J* = 2.5 Hz, Ar), 6.25 (dd, 1H, *J* = 8.4, 2.6 Hz, Ar) ppm. ^{13}C NMR (DMSO-d$_6$, 101 MHz): δ 162.6, 131.5, 129.4, **124.2**, 120.7, 117.9, 111.5 ppm.

Sodium 2-chloro-5-methylphenoxide * CO_2 (2j)

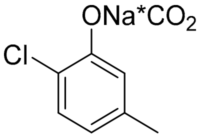

^{1}H NMR (DMSO-d$_6$, 400 MHz): δ 6.92 (d, 1H, *J* = 7.8 Hz, Ar), 6.44 (s, 1H, Ar), 5.98 (d, 1H, *J* = 7.8 Hz, Ar), 2.09 (s, 3H, Me) ppm. ^{13}C NMR (DMSO-d$_6$, 101 MHz): δ 163.7, 136.2, 127.9, **125.5**, 120.6, 119.4, 111.5, 21.1 ppm.

Sodium 4-chloro-3-ethylphenoxide* CO_2 (2k)

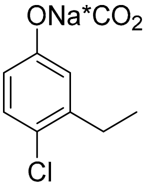

^{1}H NMR (DMSO-d$_6$, 400 MHz): δ 6.94 (d, 1H, *J* = 8.6 Hz, Ar), 6.53 (d, 1H, *J* = 2.7 Hz, Ar), 6.42 (dd, 1H, *J* = 8.6, 2.7 Hz, Ar), 2.51 (q, 2H, *J* = 7.5 Hz, CH_2), 1.11 (t, 1H, *J* = 7.5 Hz, CH_3) ppm. ^{13}C NMR (DMSO-d$_6$, 101 MHz): δ 162.9, 140.4, **139.6**, 128.8, 120.7, 118.9, 117.4, 26.5, 14.4 ppm.

Sodium 3-methylphenoxide* CO_2 (2o)

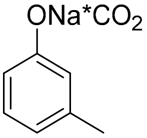

^{1}H NMR (DMSO-d$_6$, 400 MHz): δ 7.06 (t, 1H, *J* = 7.7 Hz, Ar), 6.76 (s, 1H, Ar), 6.74 (d, 1H, *J* = 8.4 Hz, Ar), 6.68 (d, 1H, *J* = 7.4 Hz, Ar), 2.24 (s, 3H, Me) ppm. ^{13}C NMR (DMSO-d$_6$, 101 MHz): δ 157.4, **149.8**, 137.7, 128.4, 122.0, 121.1, 118.4, 21.3 ppm.

NMR spectra of hydroxyaromatic carboxylic acids

4-Hydroxybenzoic acid (3a)

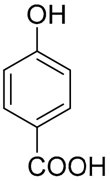

White solid. ^{1}H NMR (DMSO-d$_6$, 400 MHz): δ 12.40 (br.s, 1H, COOH), 10.21 (br.s, 1H, OH), 7.81-7.78 (m, 2H, Ar), 6.84-6.81 (m, 2H, Ar) ppm. **NMR spectrum coincides with the literature data[6].**

2-Hydroxy-3,4,6-trimethylbenzoic acid (4c)

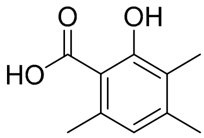

White solid. ^{1}H NMR (DMSO-d$_6$, 400 MHz): δ 13.84 (br.s, 1H, COOH), 12.25 (br.s, 1H, OH), 6.55 (s, 1H, Ar), 2.41 (s, 3H, Me), 2.18 (s, 3H, Me), 2.03 (s, 3H, Me) ppm. ^{13}C NMR (DMSO-d$_6$, 101 MHz): δ 174.1, 160.4, 142.7, 137.4, 123.9, 121.3, 109.8, 23.3, 19.9, 11.2 ppm. Anal. $M_r(C_{10}H_{12}O_3)$ = 180.079, calcd: C, 66.65; H, 6.71; found: C, 66.69; H, 6.90.

5-(*tert*-Butyl)-2-hydroxybenzoic acid (4d)

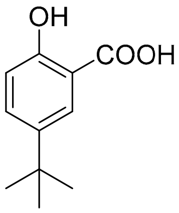

White solid. ^{1}H NMR (DMSO-d$_6$, 400 MHz): δ 13.79 (br.s, 1H, COOH), 11.18 (br.s, 1H, OH), 7.75 (s, 1H, Ar), 7.54 (d, 1H, *J* = 8.7 Hz, Ar), 6.88 (d, 1H, *J* = 8.7 Hz, Ar), 1.23 (s, 3H, *t*Bu) ppm. ^{13}C NMR (DMSO-d$_6$, 101 MHz): δ 172.2, 159.2, 141.3, 133.2, 125.9, 116.9, 112.1, 33.8, 31.1 (3C) ppm. **NMR spectra coincide with the literature data[7].**

3,5-di-(*tert*-Butyl)-2-hydroxybenzoic acid (4e)

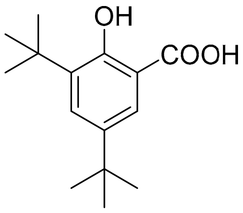

White solid. ^{1}H NMR (DMSO-d$_6$, 400 MHz): δ 13.77 (br.s, 1H, COOH), 12.08 (br.s, 1H, OH), 7.65 (s, 1H, Ar), 7.46 (s, 1H, Ar), 1.36 (s, 3H, *t*Bu), 1.24 (s, 3H, *t*Bu) ppm. ^{13}C NMR (DMSO-d$_6$, 101 MHz): δ 173.5, 158.7, 139.9, 136.6, 129.7, 123.9, 111.7, 34.7, 33.9, 31.2 (3C), 29.3 (3C) ppm. **NMR spectra coincide with the literature data[8].**

3-Allyl-4-hydroxybenzoic acid (3f)

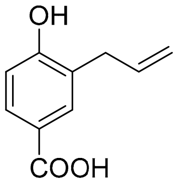

White solid. ^{1}H NMR (DMSO-d$_6$, 400 MHz): δ 12.43 (br.s, 1H, COOH), 10.24 (s, 1H, OH), 7.68-7.65 (m, 2H, Ar), 6.88-6.85 (m, 1H, Ar), 5.95 (ddt, 1H, *J* = 16.9, 10.3, 6.7 Hz, CH=), 5.08-5.02 (m, 2H, CH_2=), 3.30 (d, 2H, *J* = 6.7 Hz, CH_2) ppm. ^{13}C NMR (DMSO-d$_6$, 101 MHz): δ 167.4, 159.4, 136.6, 131.4, 129.4, 126.1,

121.3, 116.0, 114.7, 33.6 ppm. Anal. $M_r(C_{10}H_{10}O_3)$ = 178.063, calcd: C, 67.41; H, 5.66; found: C, 67.48; H, 5.70.

4-Hydroxy-3-methoxybenzoic acid (3g)

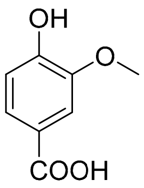

White solid. ^{1}H NMR (DMSO-d$_6$, 400 MHz): δ 10.64 (br.s, 2H, COOH and OH), 7.44-7.42 (m, 2H, Ar), 6.84-6.83 (m, 1H, Ar), 3.80 (s, 3H, OMe) ppm. **NMR spectrum coincides with the literature data[9].**

3-Chloro-4-hydroxybenzoic acid (3h)

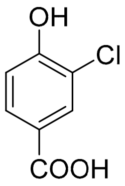

White solid. ^{1}H NMR (DMSO-d$_6$, 400 MHz): δ 12.80 (br.s, 1H, COOH), 11.09 (s, 1H, OH), 7.84 (s, 1H, Ar), 7.74 (d, 1H, J = 8.4 Hz, Ar), 7.04 (d, 1H, J = 8.5 Hz, Ar) ppm. ^{13}C NMR (DMSO-d$_6$, 101 MHz): δ 166.3, 157.3, 131.2, 129.9, 122.6, 119.7, 116.4 ppm. **NMR spectrum coincides with the literature data[10].**

5-Chloro-4-ethyl-2-hydroxybenzoic acid (4k)

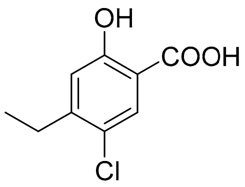

Brown solid. ^{1}H NMR (DMSO-d$_6$, 400 MHz): δ 13.73 (br.s, 1H, COOH), 11.32 (s, 1H, OH), 7.69 (s, 1H, Ar), 6.93 (s, 1H, Ar), 2.65 (q, 2H, J = 7.4 Hz, CH$_2$), 1.15 (t, 3H, J = 7.4 Hz, CH$_3$) ppm. ^{13}C NMR (DMSO-d$_6$, 101 MHz): δ 170.7, 159.9, 149.0, 129.8, 122.6, 118.2, 112.3, 26.5, 13.5 ppm. Anal. $M_r(C_9H_9ClO_3)$ = 200.024, calcd: C, 53.88; H, 4.52; found: C, 53.80; H, 4.60.

3-Chloro-6-hydroxy-2,4-dimethylbenzoic acid (4l)

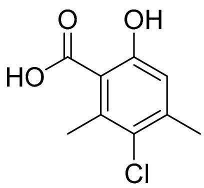

Pale beige solid. ^{1}H NMR (DMSO-d$_6$, 400 MHz): δ 11.61 (br.s, 2H, COOH and OH), 6.72 (s, 1H, Ar), 2.27 (s, 6H, 2Me) ppm. ^{13}C NMR (DMSO-d$_6$, 101 MHz): δ 169.2, 153.2, 137.8, 133.2, 123.9, 121.9, 115.9, 20.8, 17.9 ppm. Anal. $M_r(C_9H_9ClO_3)$ = 200.024, calcd: C, 53.88; H, 4.52; found: C, 53.85; H, 4.54.

4-Hydroxy-3-methylbenzoic acid (3m)

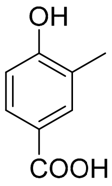

White solid. ^{1}H NMR (DMSO-d$_6$, 400 MHz): δ 12.37 (br.s, 1H, COOH), 10.15 (s, 1H, OH), 7.68 (s, 1H, Ar), 7.62 (dd, 1H, *J* = 8.4, 2.1 Hz, Ar), 6.83 (d, 1H, *J* = 8.4 Hz, Ar), 2.14 (s, 3H, Me) ppm. **NMR spectrum coincides with the literature data[7].**

4-Hydroxy-3,5-dimethoxybenzoic acid (3n)

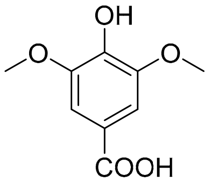

White solid. ^{1}H NMR (DMSO-d$_6$, 400 MHz): δ 12.64 (s, 1H, COOH), 9.23 (s, 1H, OH), 7.20 (s, 2H, Ar), 3.80 (s, 6H, 2OMe) ppm. **NMR spectrum coincides with the literature data[11].**

2-Hydroxy-4-methylbenzoic acid (4o)

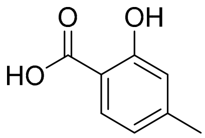

White solid. ^{1}H NMR (DMSO-d$_6$, 400 MHz): δ 11.24 (br.s, 2H, COOH and OH), 7.66 (d, 1H, *J* = 8.0 Hz, Ar), 6.76 (s, 1H, Ar), 6.72 (d, 1H, *J* = 8.1 Hz, Ar), 2.28 (s, 3H, Me) ppm. ^{13}C NMR (DMSO-d$_6$, 101 MHz): δ 172.1, 161.3, 146.6, 130.1, 120.4, 117.2, 110.3, 21.4 ppm. **NMR spectra coincide with the literature data[12].**

2-Hydroxy-1-naphthoic acid (4p)

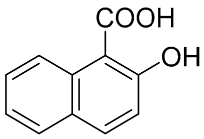

Pale beige solid. ^{1}H NMR (DMSO-d$_6$, 400 MHz): δ 12.78 (br.s, 2H, COOH and OH), 8.44 (d, 1H, *J* = 8.7 Hz, Ar), 7.99 (d, 1H, *J* = 9.0 Hz, Ar), 7.86 (d, 1H, *J* = 7.9 Hz, Ar), 7.54 (ddd, 1H, *J* = 8.4, 7.1, 1.2 Hz, Ar), 7.37 (t, 1H, *J* = 7.4 Hz, Ar), 7.19 (d, 1H, *J* = 9.0 Hz, Ar) ppm. **NMR spectrum coincides with the literature data[7].**

8-Hydroxyquinoline-7-carboxylic acid (4q)

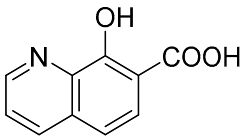

Beige solid. ^{1}H NMR (DMSO-d$_6$, 400 MHz): δ 14.76 (br.s, 2H, COOH and OH), 8.92 (d, 1H, J = 3.8 Hz, Ar), 8.61 (d, 1H, J = 8.2 Hz, Ar), 7.91 (d, 1H, J = 8.6 Hz, Ar), 7.80 (dd, 1H, J = 8.3, 4.6 Hz, Ar), 7.31 (d, 1H, J = 8.7 Hz, Ar) ppm. **NMR spectrum coincides with the literature data[13].**

Preparation of a chiral Ellman's sulfonamide with a phenolic group

***(S,E)-N*-(2-Hydroxy-3-methoxybenzylidene)-2-methylpropane-2-sulfinamide**

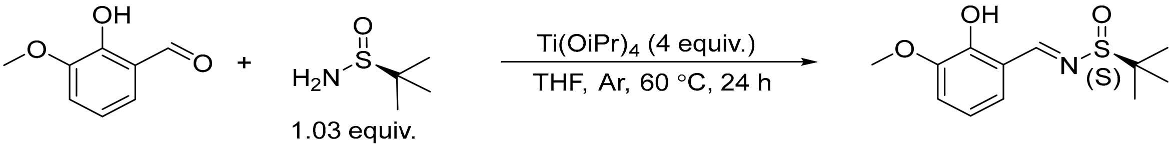

ortho-Vanillin (2.28 g, 15.0 mmol), (*S*)-2-methyl-2-propanesulfinamide (1.87 g, 15.5 mmol) and Ti(O*i*Pr)$_4$ (17.1 g, 17.8 mL, 60.0 mmol) in THF (15 mL) were placed in a 50 mL flask under Ar. The flask was stoppered, and the solution was stirred while heating on an oil bath at 60 °C for 24 h. The resulting mixture was gradually poured into a mixture of water (180 mL) and DCM (50 mL) and stirred for 3 h until a yellowish precipitate formed. The resulting suspension was filtered through a Hyflo® Super-Cell® filter pad, the filter pad was carefully washed with DCM (3 x 20 mL). The organic phase was separated, the aqueous phase was extracted with DCM (20 mL), and the combined organic extracts were dried over $MgSO_4$. The resulting solution was evaporated on a rotatory evaporator. The residual oil was recrystallized from hexane to give Ellman's imine (3.38 g, 88%) as a beige solid, m.p. 118-120 °C (hexane), R_f 0.20 (hexane/EtOAc, 4:1). ^{1}H NMR ($CDCl_3$, 400 MHz): δ 11.25 (s, 1H, OH), 8.67 (s, 1H, CH=N), 7.07 (dd, 1H, J = 7.8, 1.4 Hz, Ar), 7.02 (dd, 1H, J = 8.1, 1.0 Hz, Ar), 6.90 (t, 1H, J = 7.9 Hz, Ar), 3.89 (s, 3H, OMe), 1.22 (s, 9H, *t*Bu) ppm. ^{13}C NMR ($CDCl_3$, 101 MHz): δ 165.4, 150.2, 148.4, 124.5, 119.5, 118.4, 116.0, 57.9, 56.2, 22.2 (3C) ppm. **NMR spectra coincide with the literature data[14].**

***(S)-N-((S)*-1-(2-Hydroxy-3-methoxyphenyl)but-3-en-1-yl)-2-methylpropane-2-sulfinamide**

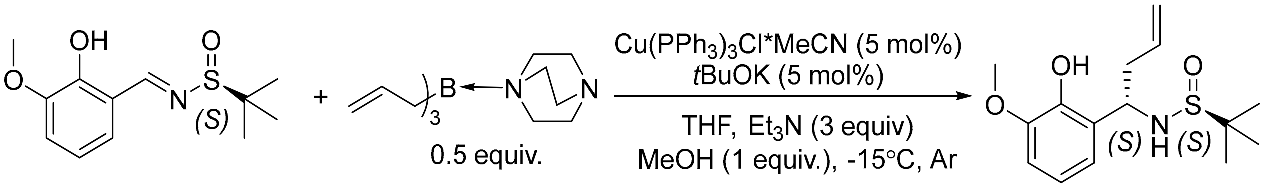

Ellman's imine (10.0 mmol) and Cu(PPh$_3$)$_3$Cl*MeCN (0.463 g, 0.5 mmol) were dissolved in THF (25 mL) under Ar. To this solution at -15 °C were added *t*BuOK solution (1.0 M in THF, 0.5 mL, 0.5 mmol), Et_3N (3.02 g, 4.18 mL, 30.0 mmol) and TABDABCO (1.23 g, 5.0 mmol, 0.5 equiv.) and the solution was stirred for 1.5 h at this temperature. A solution of MeOH (0.32 g, 0.4 mL, 10.0 mmol, 1.0 equiv.) in THF (10 mL) was added to the formed mixture via syringe pump over 1 h, after which the mixture was stirred for another 1h and quenched with AcOH (3.0 mL, 50.0 mmol) at -15 °C. The mixture was diluted with EtOAc (15 mL) and a mixture of K_2CO_3 solution and 25% aq.NH_3 (for copper extraction). The organic layer was separated and washed several times with a mixture of brine and 25% aq.NH_3 until the blue coloration disappeared. The resulting extract was dried over $MgSO_4$ and evaporated to give the crude homoallylamine as a viscous yellow oil. This product was purified by column chromatography (hexane/EtOAc 1:1) to afford the homoallylamine

(2.56 g, 86%) as a white solid, m.p. 87-89 °C (hexane/EtOAc), R_f = 0.29 (hexane/EtOAc 1:1), *de* >99% (NMR), $[\alpha]_D^{25}$ +40.3 (*c* 1.0, $CHCl_3$). ^{1}H NMR ($CDCl_3$, 400 MHz): δ 6.80-6.75 (m, 2H, Ar), 6.75-6.71 (m, 1H, Ar), 6.36 (s, 1H, OH), 5.69 (ddt, 1H, *J* = 17.2, 10.2, 7.0 Hz, CH=), 5.06-4.98 (m, 2H, CH_2=), 4.51 (q, 1H, *J* = 7.3 Hz, CH), 4.20 (d, 1H, *J* = 7.7 Hz, NH), 3.79 (s, 3H, OMe), 2.74-2.67 (m, 1H, CH_2), 2.61-2.54 (m, 1H, CH_2), 1.20 (s, 9H, *t*Bu) ppm. ^{13}C NMR ($CDCl_3$, 101 MHz): δ 146.9, 143.2, 135.0, 128.1, 120.1, 119.6, 117.4, 109.8, 57.3, 56.2, 55.9, 40.4, 22.8 (3C) ppm. Anal. $M_r(C_{15}H_{23}NO_3S)$ = 297.140, calcd: C, 60.58; H, 7.80; N, 4.71; found: C, 60.61; H, 7.92; N, 4.58.

3-((*S*)-1-(((*S*)-*tert*-Butylsulfinyl)amino)but-3-en-1-yl)-4-hydroxy-5-methoxybenzoic acid (3r)

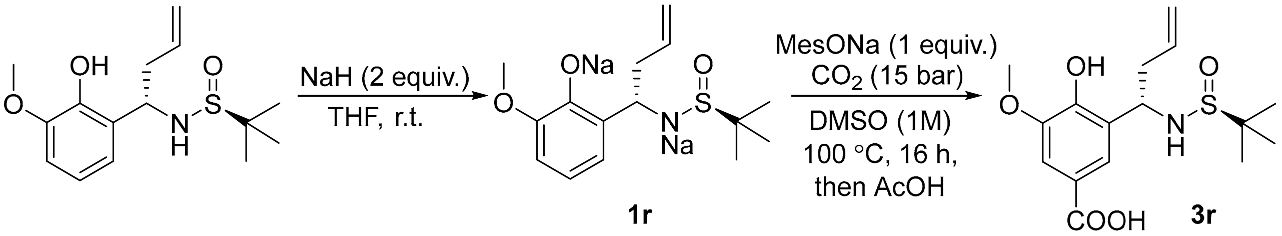

Under Ar atmosphere NaH (60% dispersion in mineral oil) (120 mg, 3.0 mmol, 2.0 equiv.) was washed with *n*-hexane (3 x 3 mL) to remove oil. To the washed NaH were added THF (5 mL) and a solution of phenol (446 mg, 1.5 mmol) in THF (5 mL). After 1 h, when H_2 evolution ceased, the solvent was evaporated under reduced pressure (15 Torr), and the disodium salt **1r** was dried under vacuum (0.1 mbar) on an oil bath (100 °C) for 3 h, yielding **1r** as a pale yellow solid. Under Ar atmosphere **1r** (341 mg, 1.0 mmol), MesONa (158 mg, 1.0 mmol, 1.0 equiv.), and DMSO (1 mL) were placed into a steel pressure reactor (20 mL) equipped with a magnetic stirring bar. The reactor was sealed under 15 bar of CO_2 and immersed into an oil bath (100 °C) with stirring for 16 h. The reaction mixture was cooled; the CO_2 pressure was slowly released (≈ 10 min). $NaHCO_3$ (sat. solution) (2 ml, 2 equiv.) was added to the reaction mixture, and organic impurities were removed by extraction with EtOAc (3 x 5 mL). The aqueous phase was acidified with AcOH (577 µL, 10 equiv.), and the product was extracted with EtOAc (3 x 5 mL). The combined organic extract was washed with NaCl (sat solution) (1 x 5 mL), dried over Na_2SO_4 and evaporated to dryness. The residue was purified by column chromatography (EtOAc) to give **3r** (252 mg, 74%) as a pale beige solid. R_f = 0.15 (EtOAc), m.p. = 108-110 °C (EtOAc), $[\alpha]_D^{25}$ +132.3 (*c* 1.0, $CHCl_3$). ^{1}H NMR (DMSO-d_6, 400 MHz): δ 12.52 (br.s, 1H, COOH), 9.57 (br.s, 1H, OH), 7.69 (d, 1H, *J* = 2.0 Hz, Ar), 7.35 (d, 1H, *J* = 1.9 Hz, Ar), 5.78-5.67 (m, 2H, CH= and NH), 5.02-4.94 (m, 2H, CH_2=), 4.63 (td, 1H, *J* = 8.6, 5.8 Hz, CH), 3.84 (s, 3H, OMe), 2.50-2.43 (m, 1H, CH_2), 2.38-2.31 (m, 1H, CH_2), 1.08 (s, 9H, *t*Bu) ppm. ^{13}C NMR (DMSO-d_6, 101 MHz): δ 167.5, 147.2, 146.6, 135.6, 130.3, 122.3, 121.0, 116.9, 110.4, 55.8, 55.7, 53.1, 40.9, 22.6 (3C) ppm. Anal. $M_r(C_{16}H_{23}NO_5S)$ = 341.130, calcd: C, 56.29; H, 6.79; N, 4.10; found: C, 56.20; H, 6.82; N, 4.08.

References

[1] Borys, A.M. An Illustrated Guide to Schlenk Line Techniques. *Organometallics* **2023**, *42*, 182–196. https://doi.org/10.1021/acs.organomet.2c00535.

[2] Merzliakov, D.A.; Alexeev, M.S.; Topchiy, M.A.; Yakhvarov, D.G.; Kuznetsov, N.Y.; Maximov, A.L.; Beletskaya, I.P. Development of Homogeneous Carboxylation of Phenolates via Kolbe–Schmitt Reaction. *Molecules* **2025**, *30*, 248. https://doi.org/10.3390/molecules30020248.

[3] Alexeev, M.S.; Strelkova, T.V.; Ilyin, M.M.; Nelyubina, Y.V.; Bespalov, I.A.; Medvedev, M.G.; Khrustalev, V.N.; Kuznetsov, N.Y. Amine Adducts of Triallylborane as Highly Reactive Allylborating Agents for Cu(I)-Catalyzed Allylation of Chiral Sulfinylimines. *Org. Biomol. Chem.* **2024**, *22*, 4680–4696. https://doi.org/10.1039/D4OB00291A.

[4] Kuznetsov, N.Y.; Tikhov, R.M.; Strelkova, T.V.; Bubnov, Y.N. Dimethylamine Adducts of Allylic Triorganoboranes as Effective Reagents for Petasis-Type Homoallylation of Primary Amines with Formaldehyde. *Org. Biomol. Chem.* **2018**, *16*, 7115–7119. https://doi.org/10.1039/C8OB02152J.

[5] Burfield, D.R.; Smithers, R.H. Desiccant Efficiency in Solvent Drying. 3. Dipolar Aprotic Solvents. *J. Org. Chem.* **1978**, *43*, 3966–3968. https://doi.org/10.1021/jo00414a038.

[6] Zhao, D.; Wu, N.; Zhang, S.; Xi, P.; Su, X.; Lan, J.; You, J. Synthesis of Phenol, Aromatic Ether, and Benzofuran Derivatives by Copper-Catalyzed Hydroxylation of Aryl Halides. *Angew. Chem. Int. Ed.* **2009**, *48*, 8729–8732. https://doi.org/10.1002/anie.200903923.

[7] Wang, Y.; Gevorgyan, V. General Method for the Synthesis of Salicylic Acids from Phenols Through Palladium-Catalyzed Silanol-Directed C—H Carboxylation. *Angew. Chem. Int. Ed.* **2015**, *54*, 2255–2259. https://doi.org/10.1002/anie.201410375.

[8] Scheurer, A.; Mosset, P.; Bauer, W.; Saalfrank, R.W. A Practical Route to Regiospecifically Substituted (R)- and (S)-Oxazolylphenols. *Eur. J. Org. Chem.* **2001**, *2001*, 3067.

[9] Bao, K.; Fan, A.; Dai, Y.; Zhang, L.; Zhang, W.; Cheng, M.; Yao, X. Selective Demethylation and Debenzylation of Aryl Ethers by Magnesium Iodide Under Solvent-Free Conditions and Its Application to the Total Synthesis of Natural Products. *Org. Biomol. Chem.* **2009**, *7*, 5084–5090. https://doi.org/10.1039/b916969e.

[10] Jacques Dit Lapierre, T.J.W.; Cruz, M.G.F.D.M.L.; Brito, N.P.F.; Resende, D.D.M.; Souza, F.D.O.; Pilau, E.J.; da Silva, M.F.B.; Neves, B.J.; Murta, S.M.F.; Rezende Júnior, C.D.O. Hit-to-Lead Optimization of a Pyrazinylpiperazine Series Against Leishmania Infantum and Leishmania Braziliensis. *Eur. J. Med. Chem.* **2023**, *256*, 115445. https://doi.org/10.1016/j.ejmech.2023.115445.

[11] Shi, S.; Zhao, Y.; Zhou, H.; Zhang, Y.; Jiang, X.; Huang, K. Identification of Antioxidants from Taraxacum Mongolicum by High-Performance Liquid Chromatography–Diode Array Detection–Radical-Scavenging Detection–Electrospray Ionization Mass Spectrometry and Nuclear Magnetic Resonance Experiments. *J. Chromatogr. A* **2008**, *1209*, 145–152. https://doi.org/10.1016/j.chroma.2008.09.004.

[12] Luo, J.; Preciado, S.; Xie, P.; Larrosa, I. Carboxylation of Phenols with CO_2 at Atmospheric Pressure. *Chem. A Eur. J.* **2016**, *22*, 6798–6802. https://doi.org/10.1002/chem.201601114.

[13] Takayama, T.; Kitamura, M.; Kobayashi, Y.; Arakawa, Y.; Kudo, K. Synthesis of Alq3-Pendent Soluble Polymers and Their Application to Organic Light Emitting Diode. *Kobunshi Ronbunshu* **2006**, *63*, 696–703. https://doi.org/10.1295/koron.63.696.

[14] Zhao, Y.; Ren, X.; Liu, H.; Tang, Z. Study on Synthesis of Ortho-Hydroxyl Aromatic N-Tert-Butylsulfinyl Imines Under Microwave Irradiation. *Chin. J. Org. Chem.* **2014**, *34*, 1218. https://doi.org/10.6023/cjoc201401019.

Copies of NMR spectra

^{1}H NMR (400 MHz, DMSO-d$_6$) **2a**

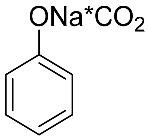

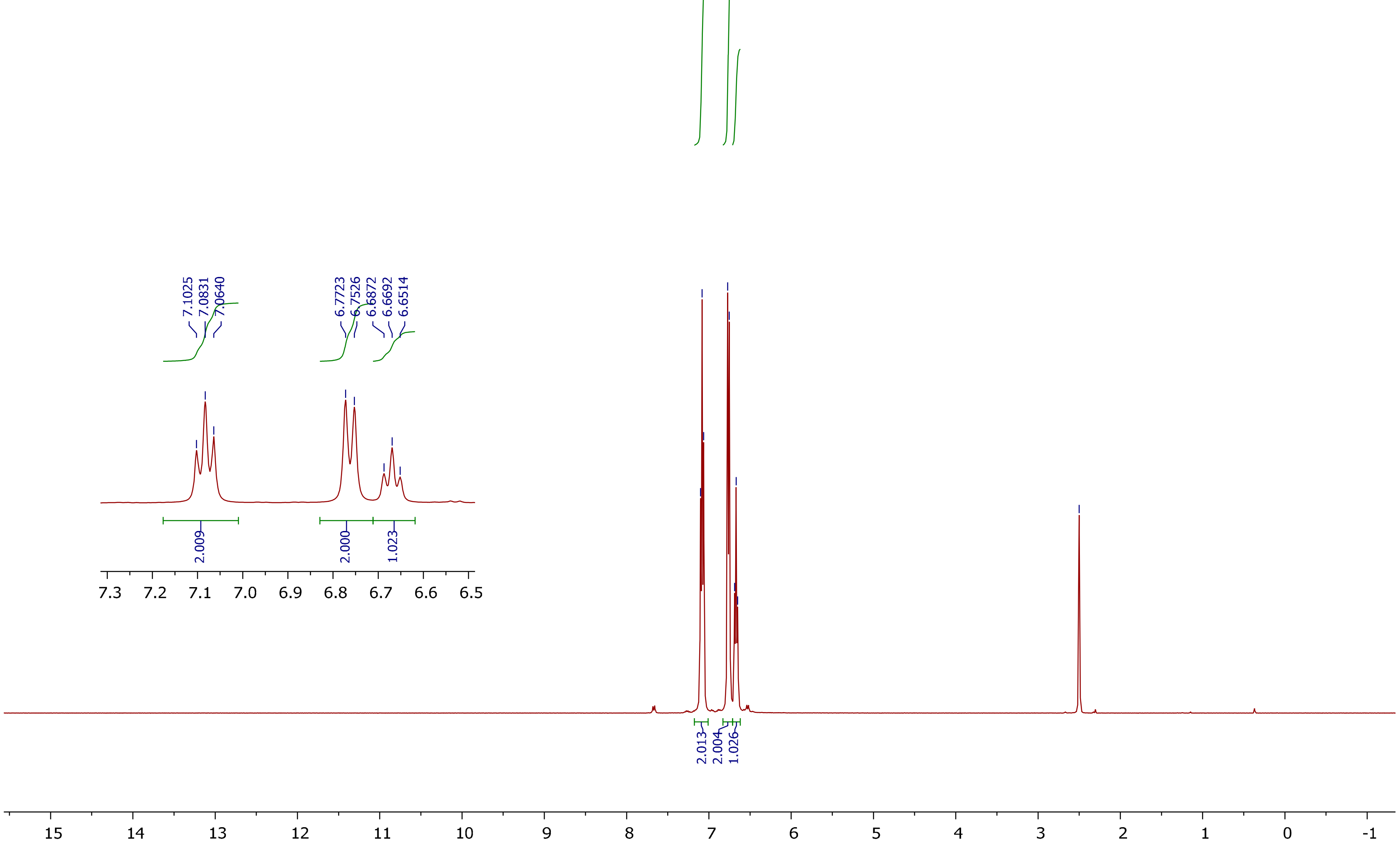

^{13}C NMR (101 MHz, DMSO-d$_6$) **2a**

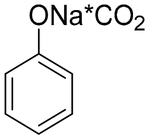

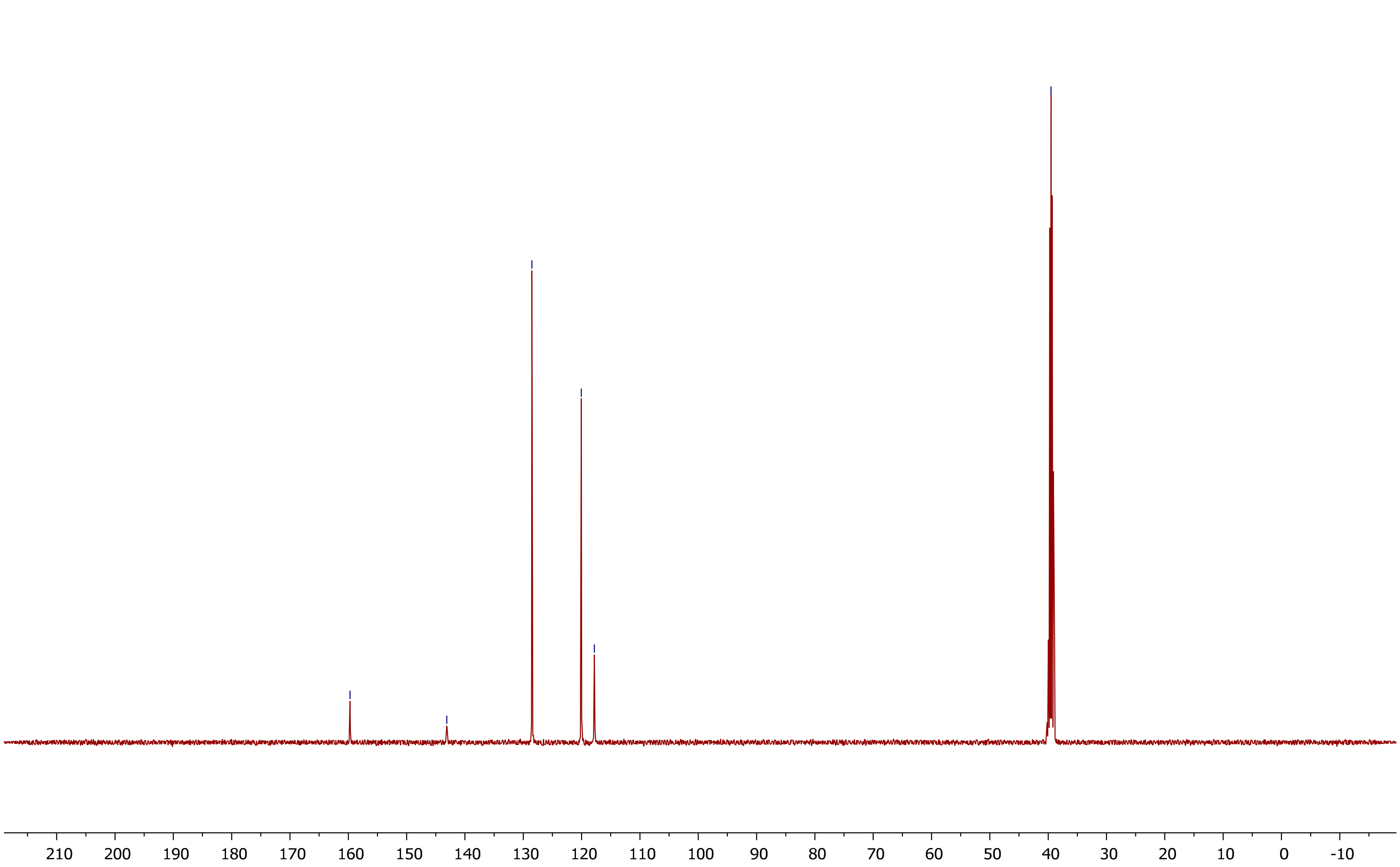

^{1}H NMR (400 MHz, DMSO-d6) **2b**

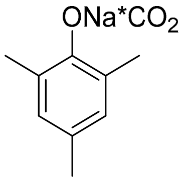

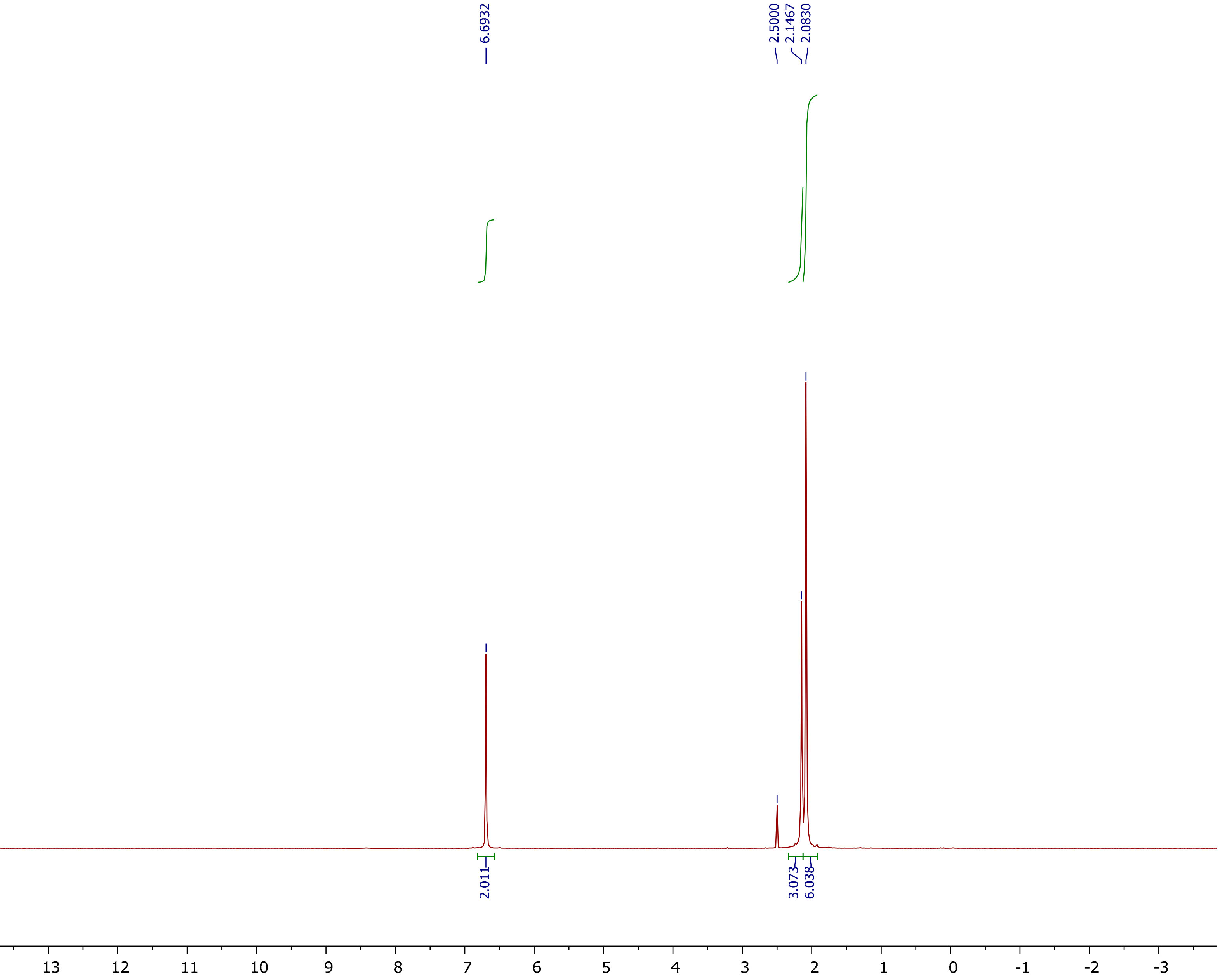

^{13}C NMR (101 MHz, DMSO-d6) **2b**

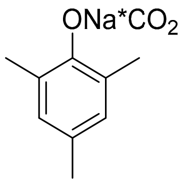

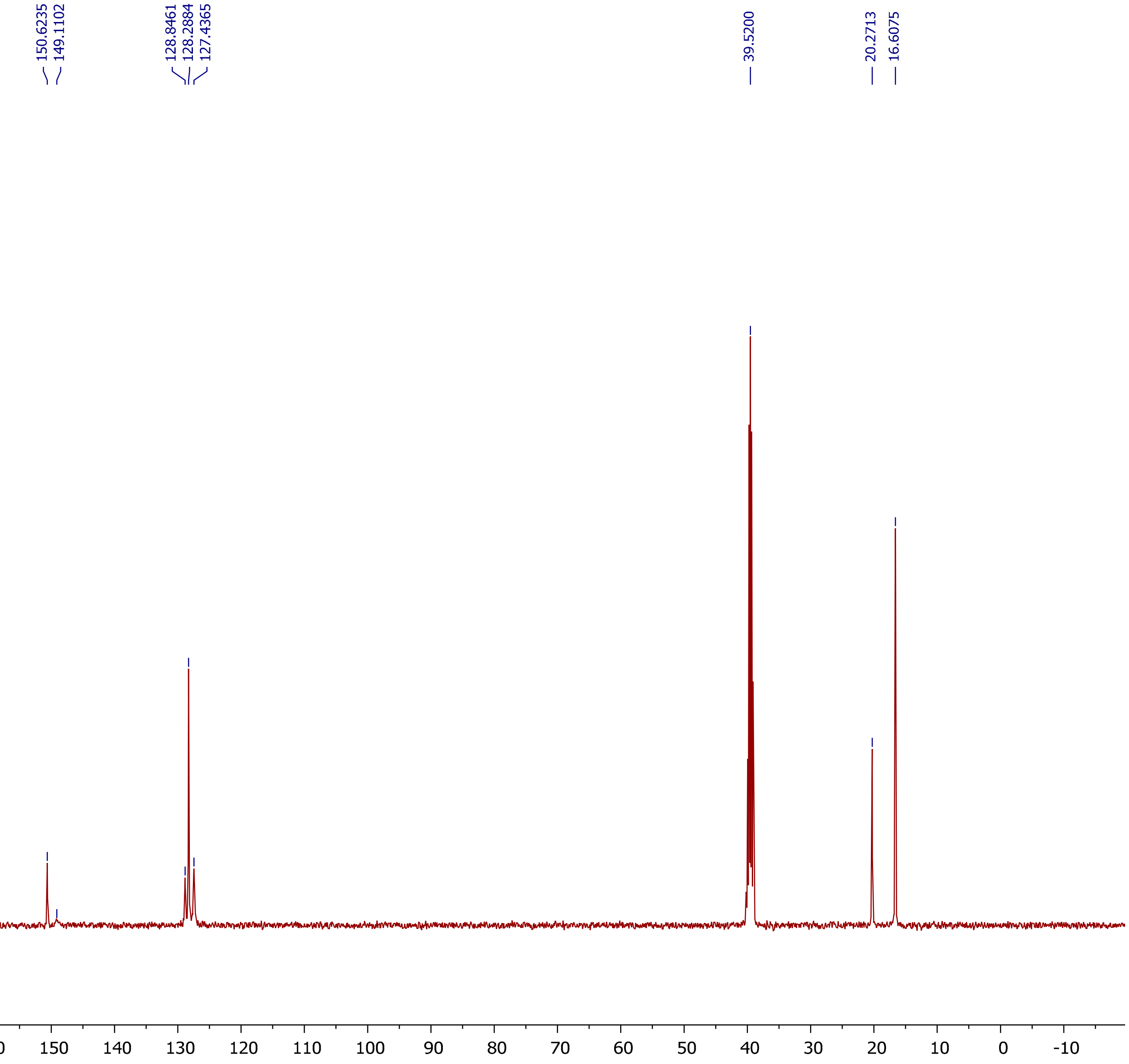

^{1}H NMR (400 MHz, DMSO-d$_6$) **2c**

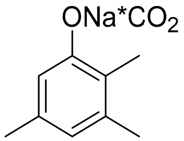

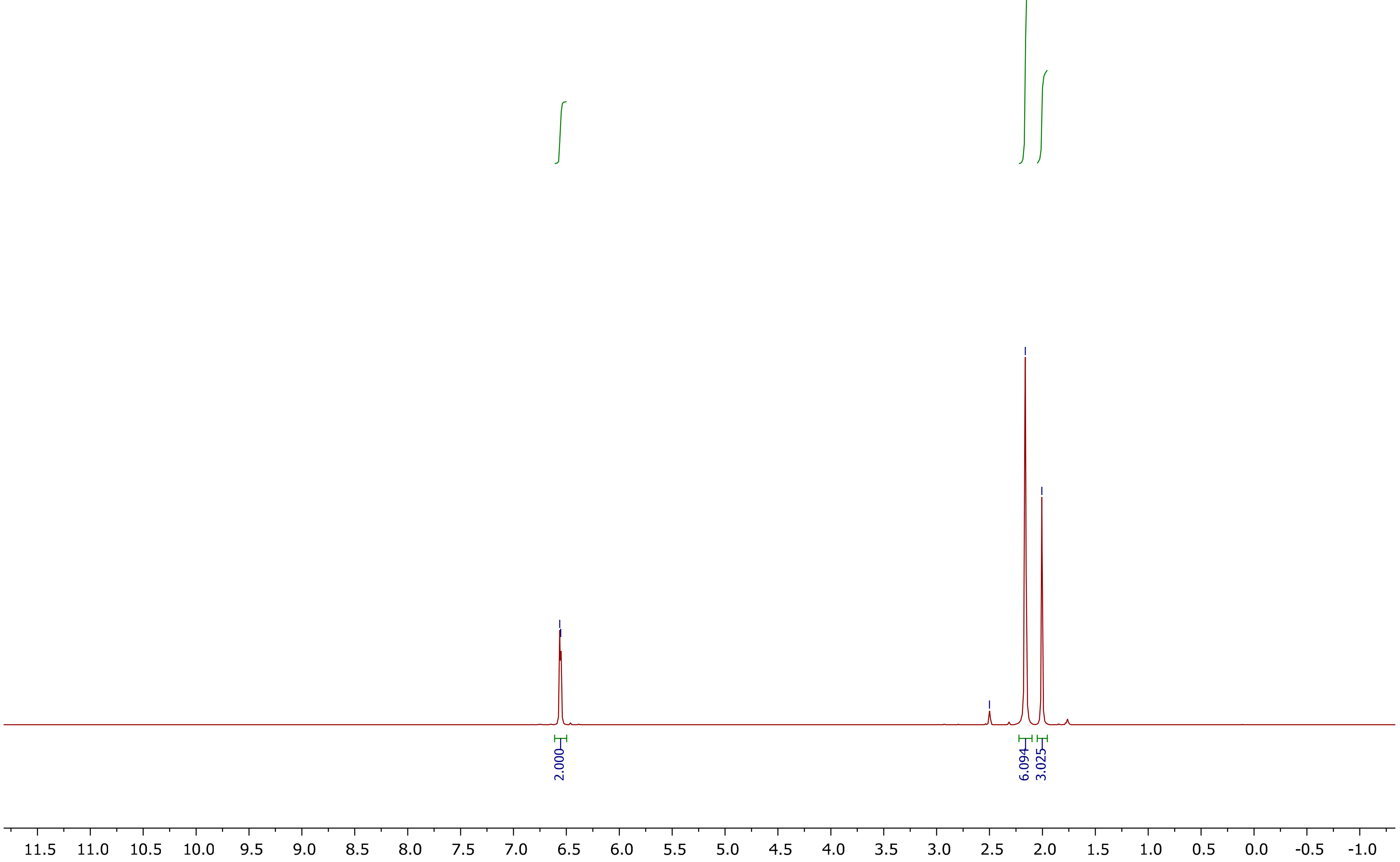

^{13}C NMR (101 MHz, DMSO-d$_6$) **2c**

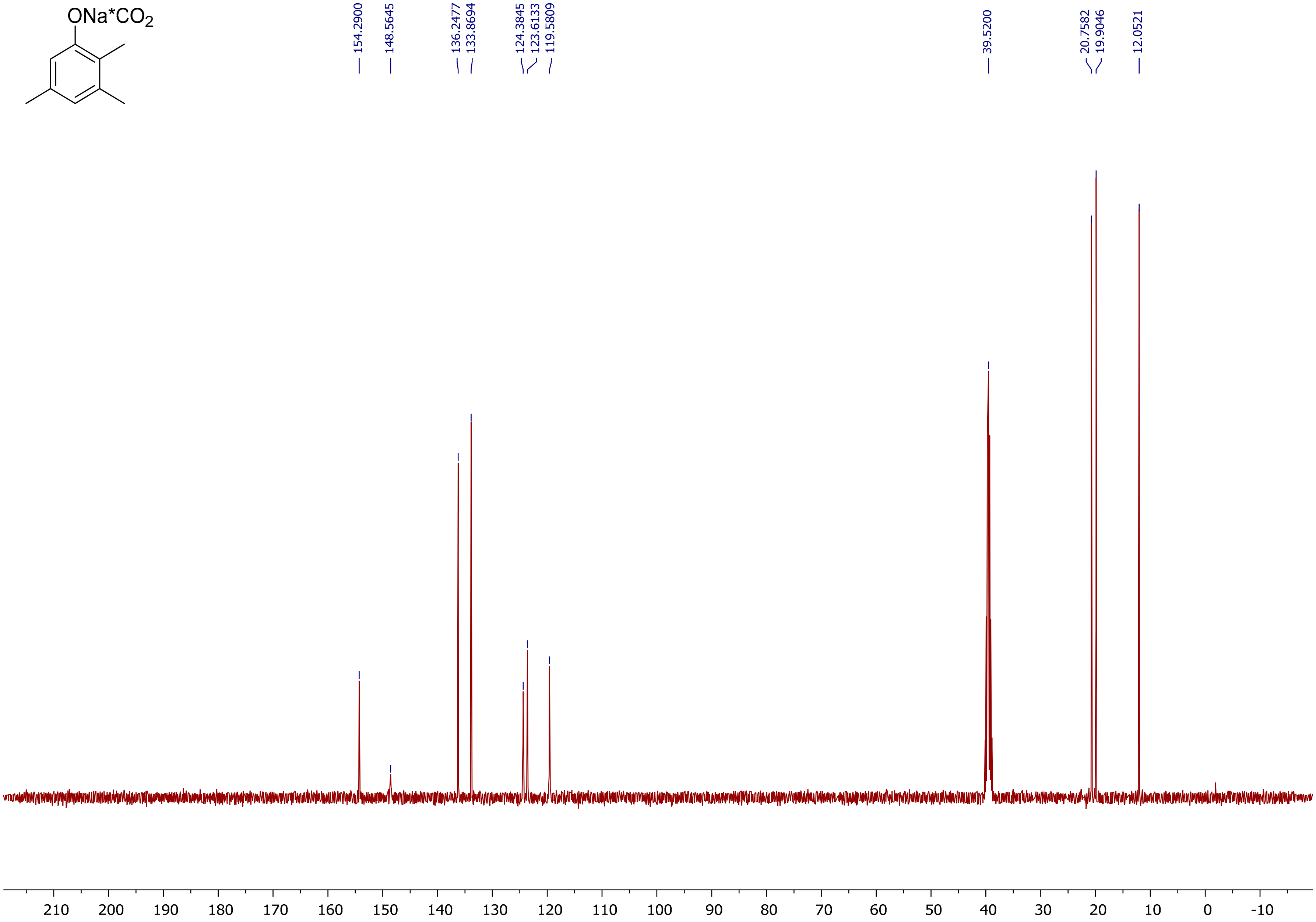

^{1}H NMR (400 MHz, DMSO-d6) **2d**

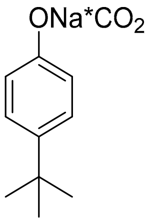

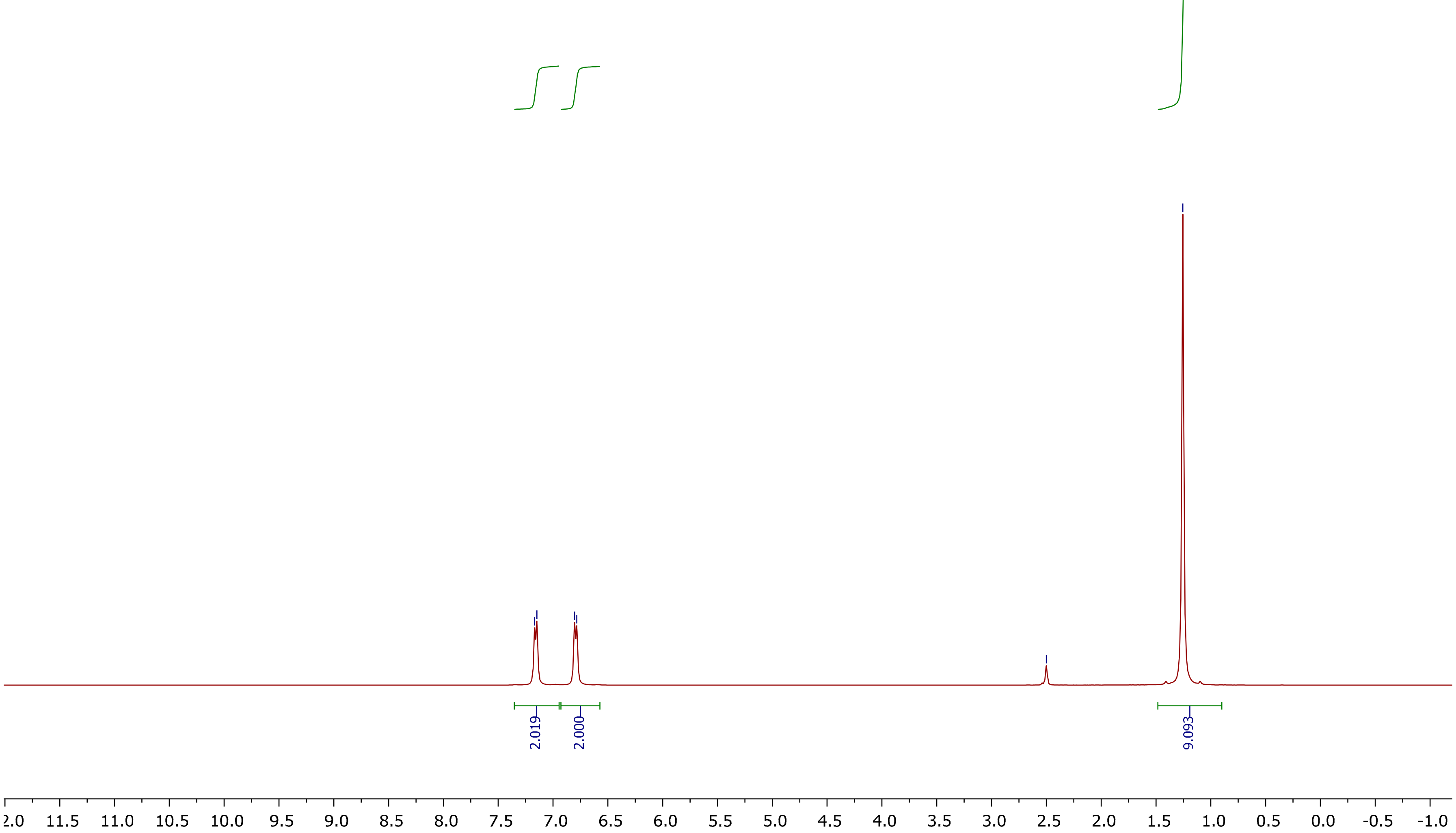

^{13}C NMR (101 MHz, DMSO-d6) **2d**

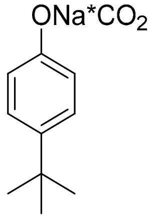

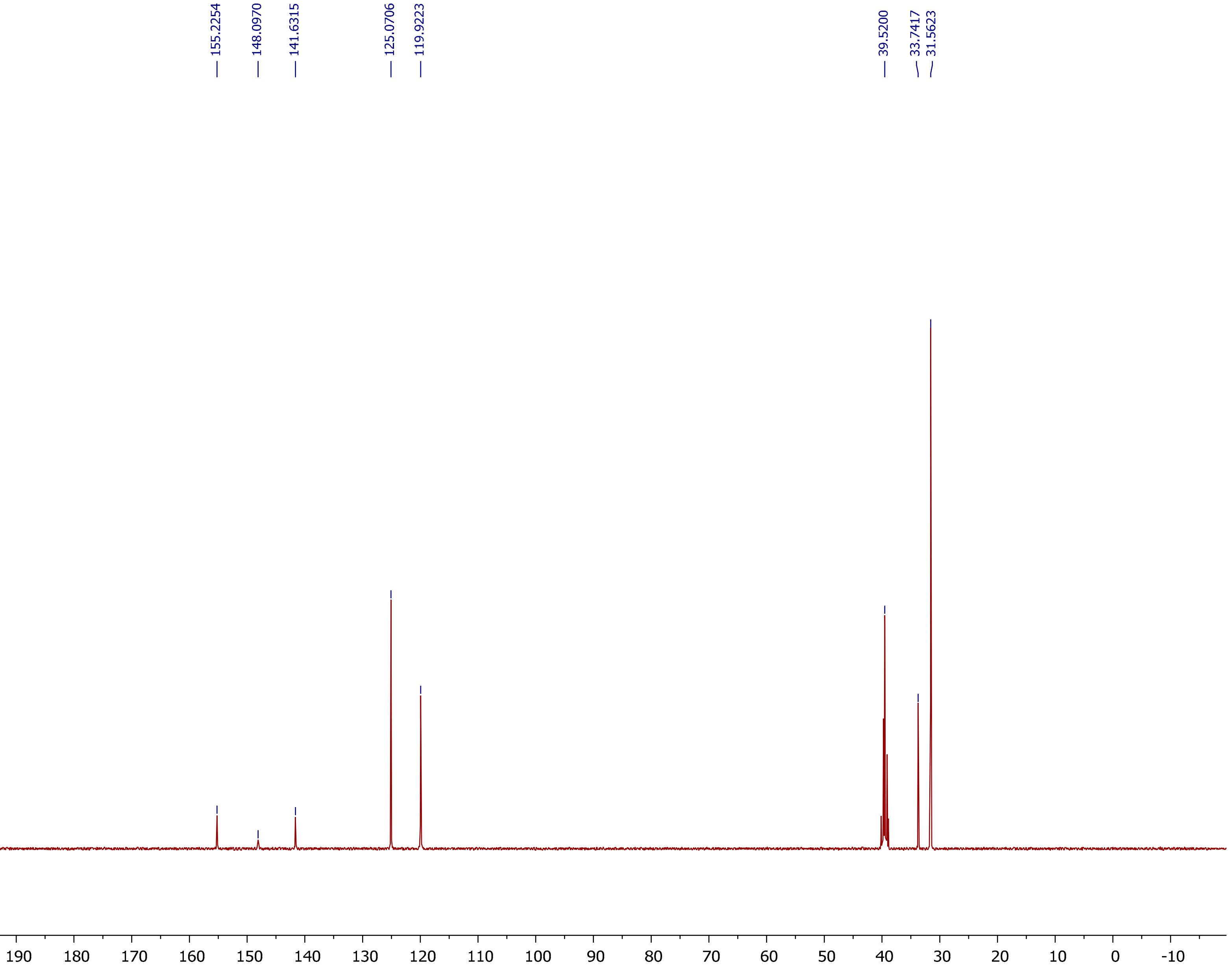

^{1}H NMR (400 MHz, DMSO-d$_6$) **2e**

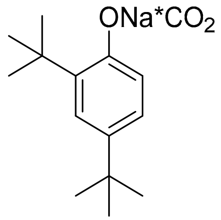

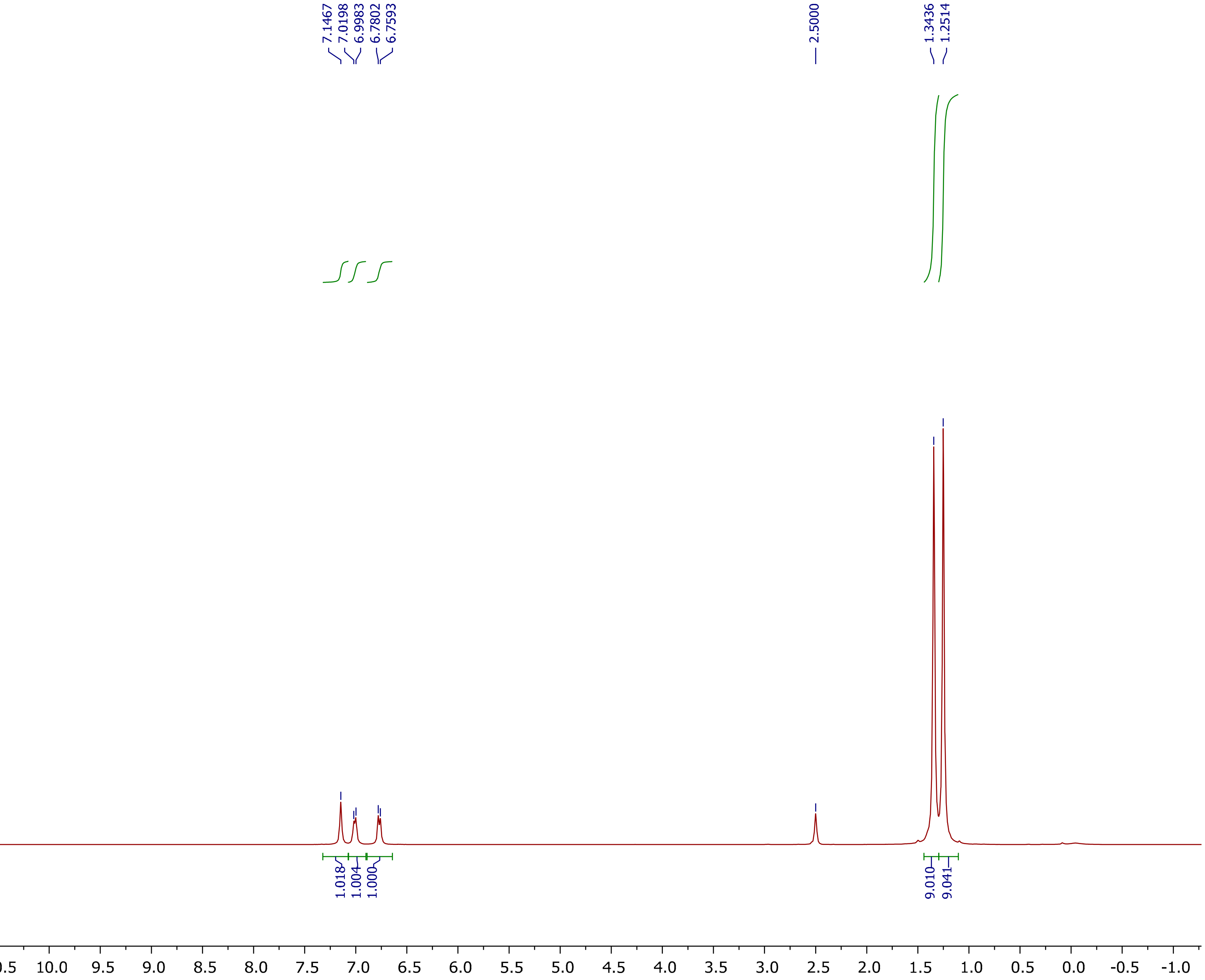

^{13}C NMR (101 MHz, DMSO-d$_6$) **2e**

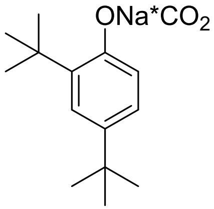

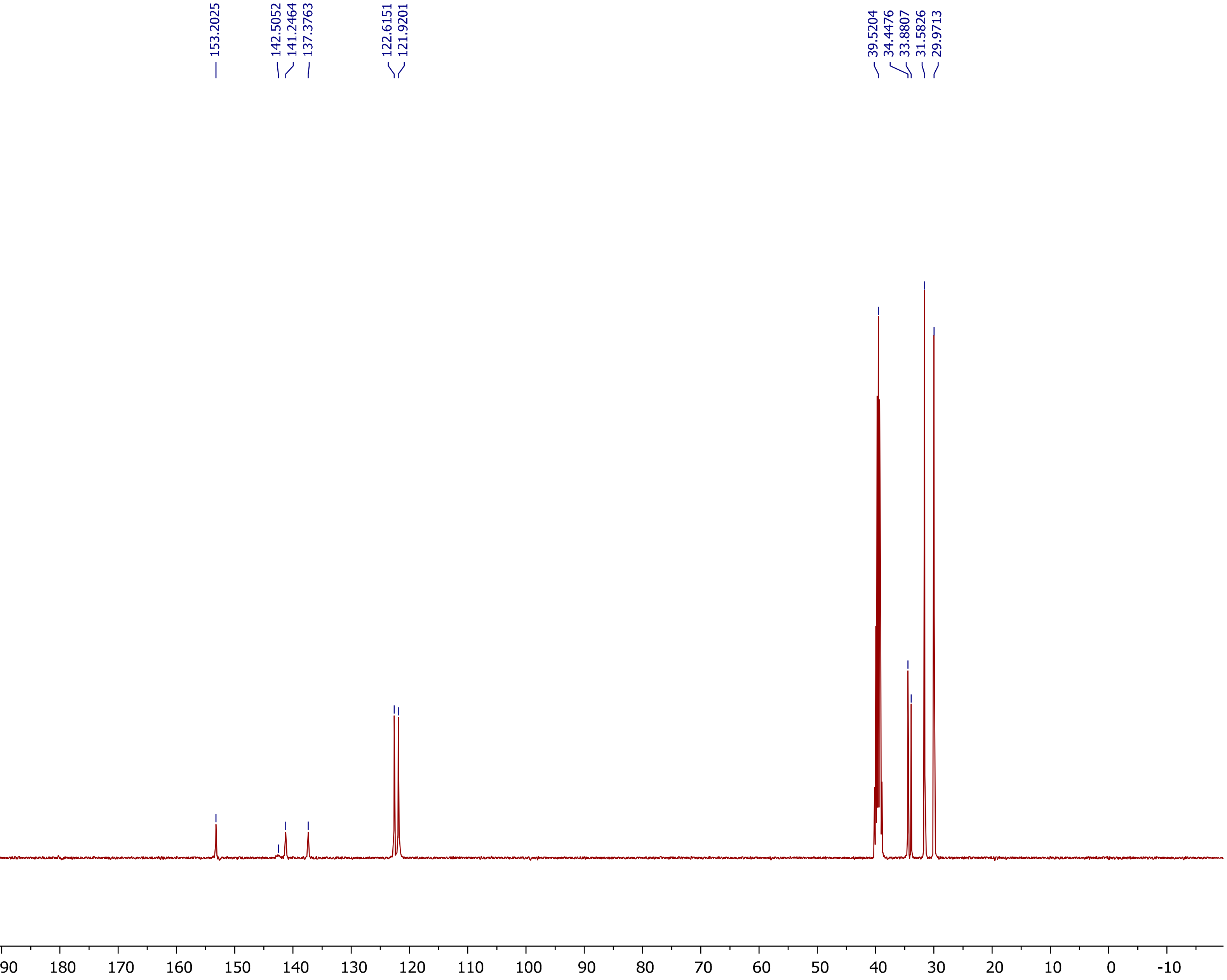

^{1}H NMR (400 MHz, DMSO-d$_6$) **2f**

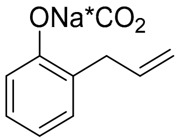

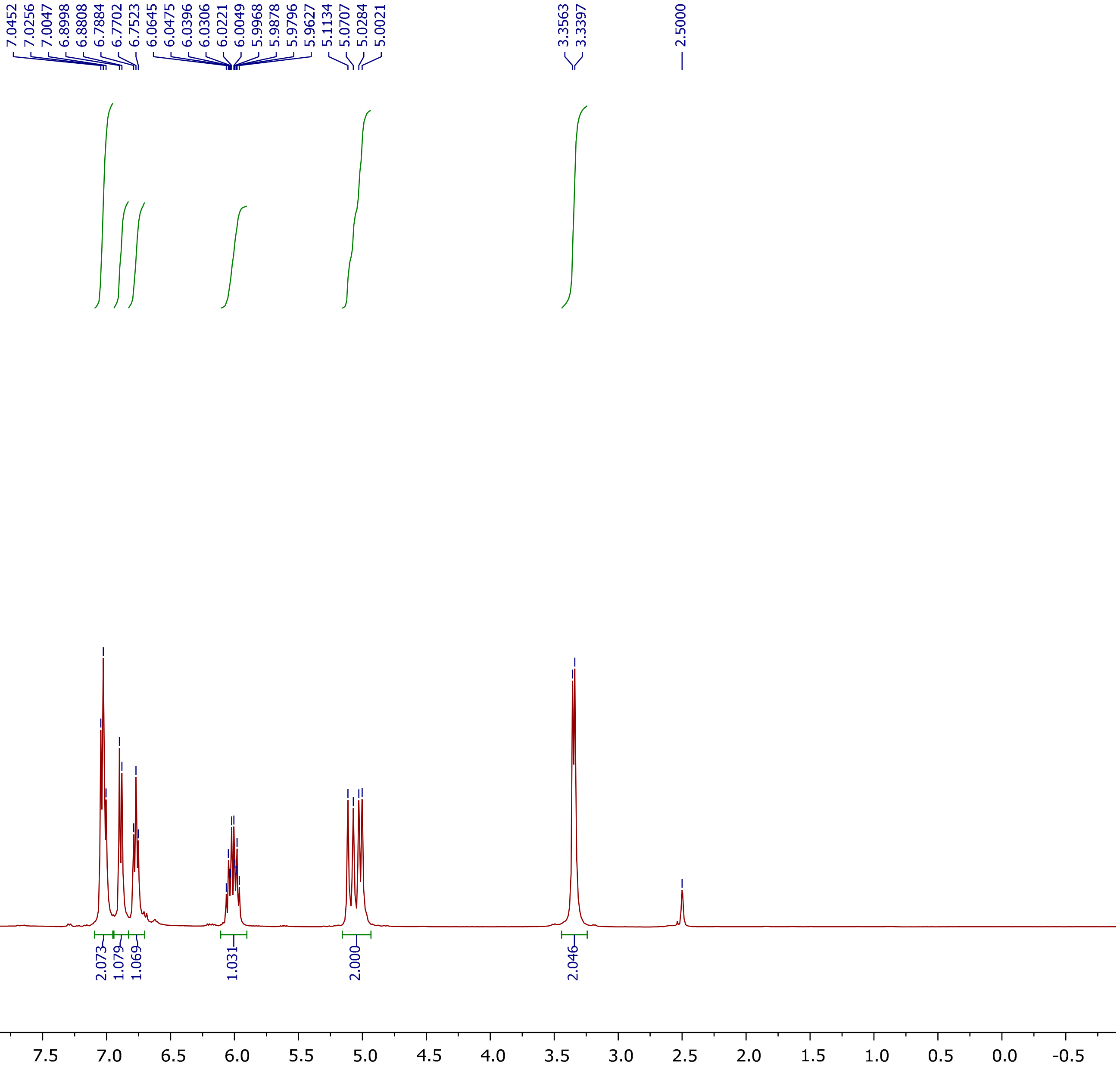

^{13}C NMR (101 MHz, DMSO-d$_6$) **2f**

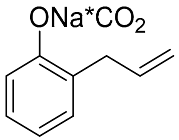

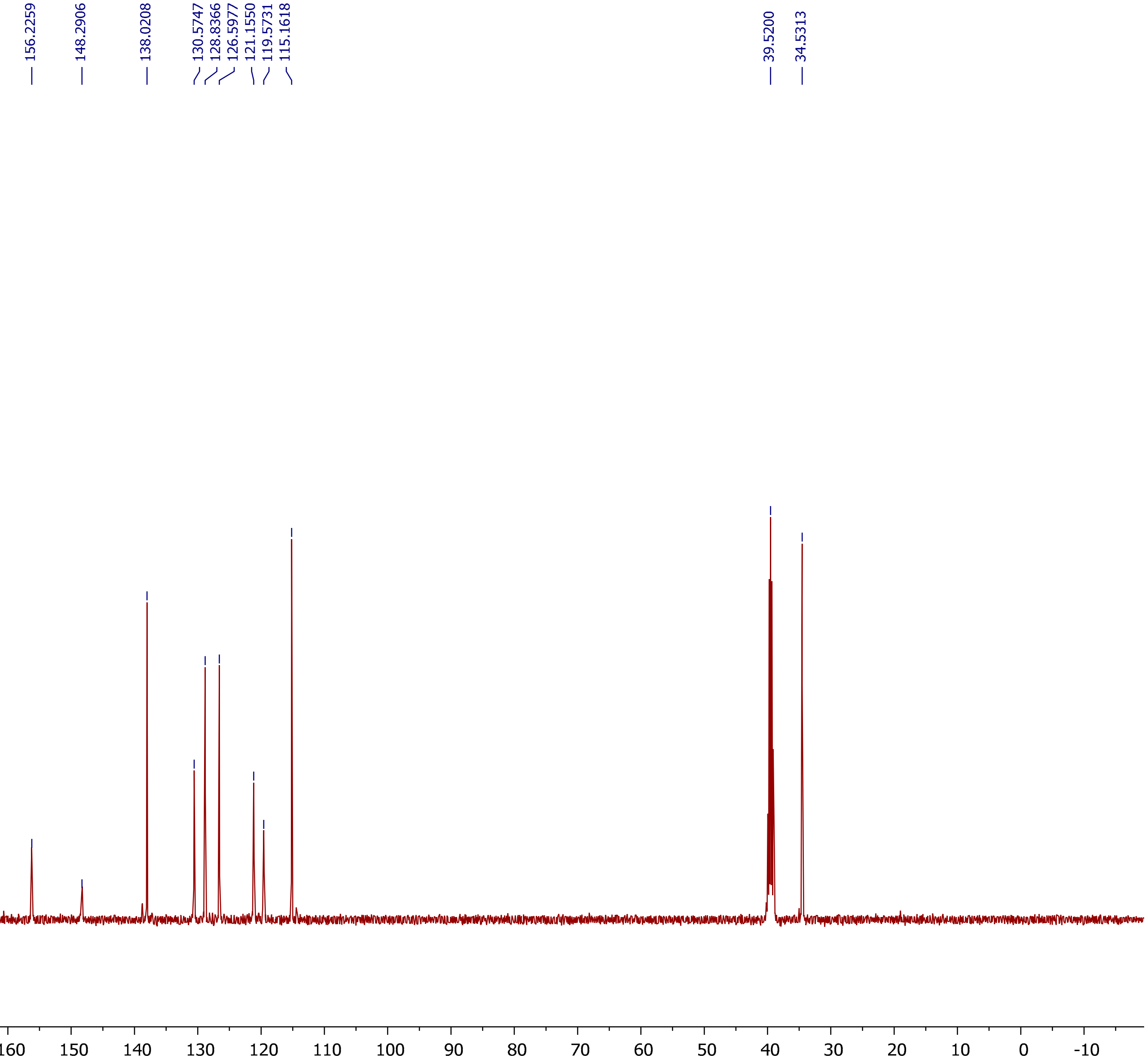

^{1}H NMR (400 MHz, DMSO-d6) **2g**

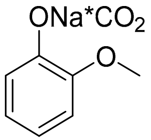

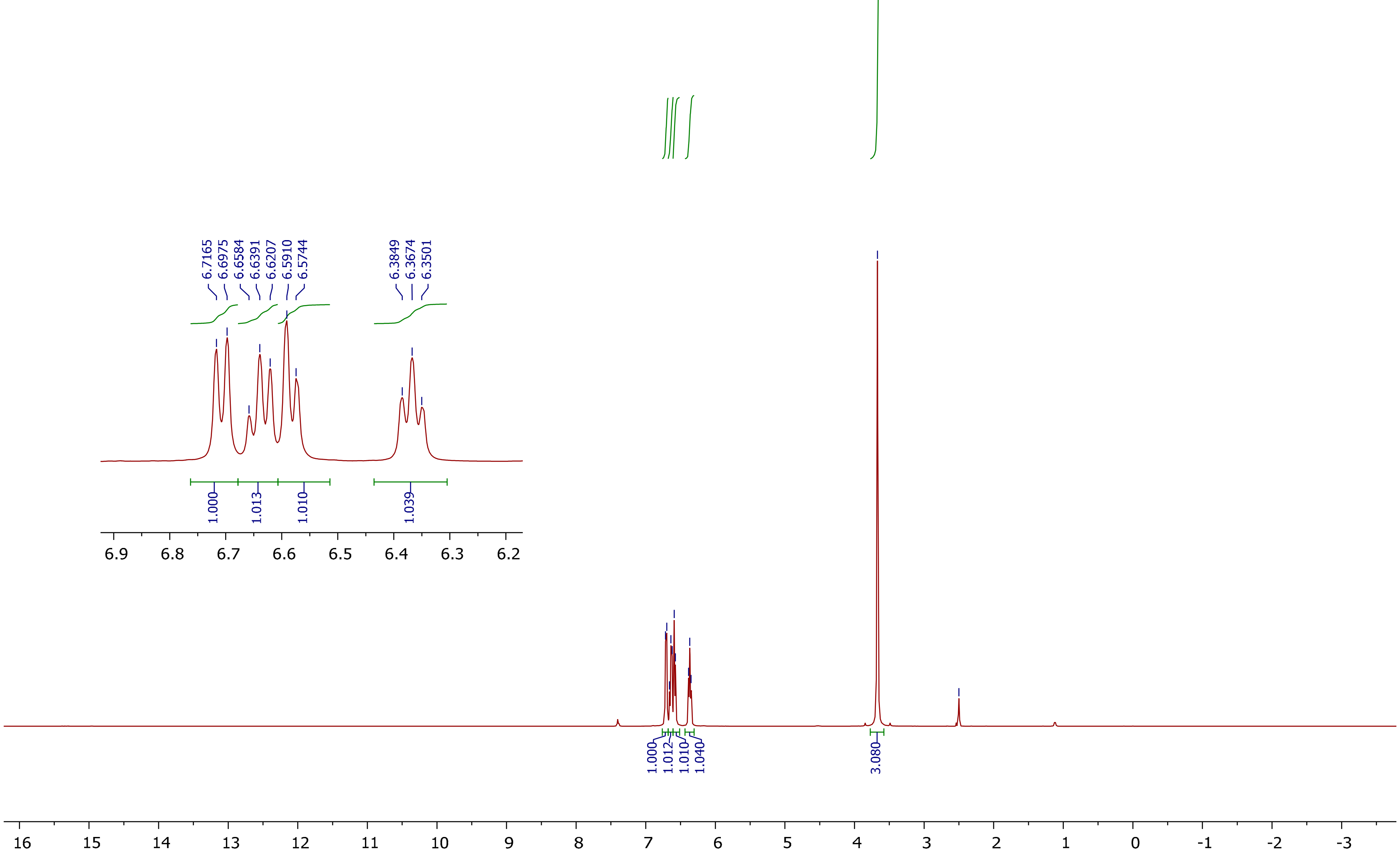

^{13}C NMR (101 MHz, DMSO-d6) **2g**

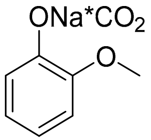

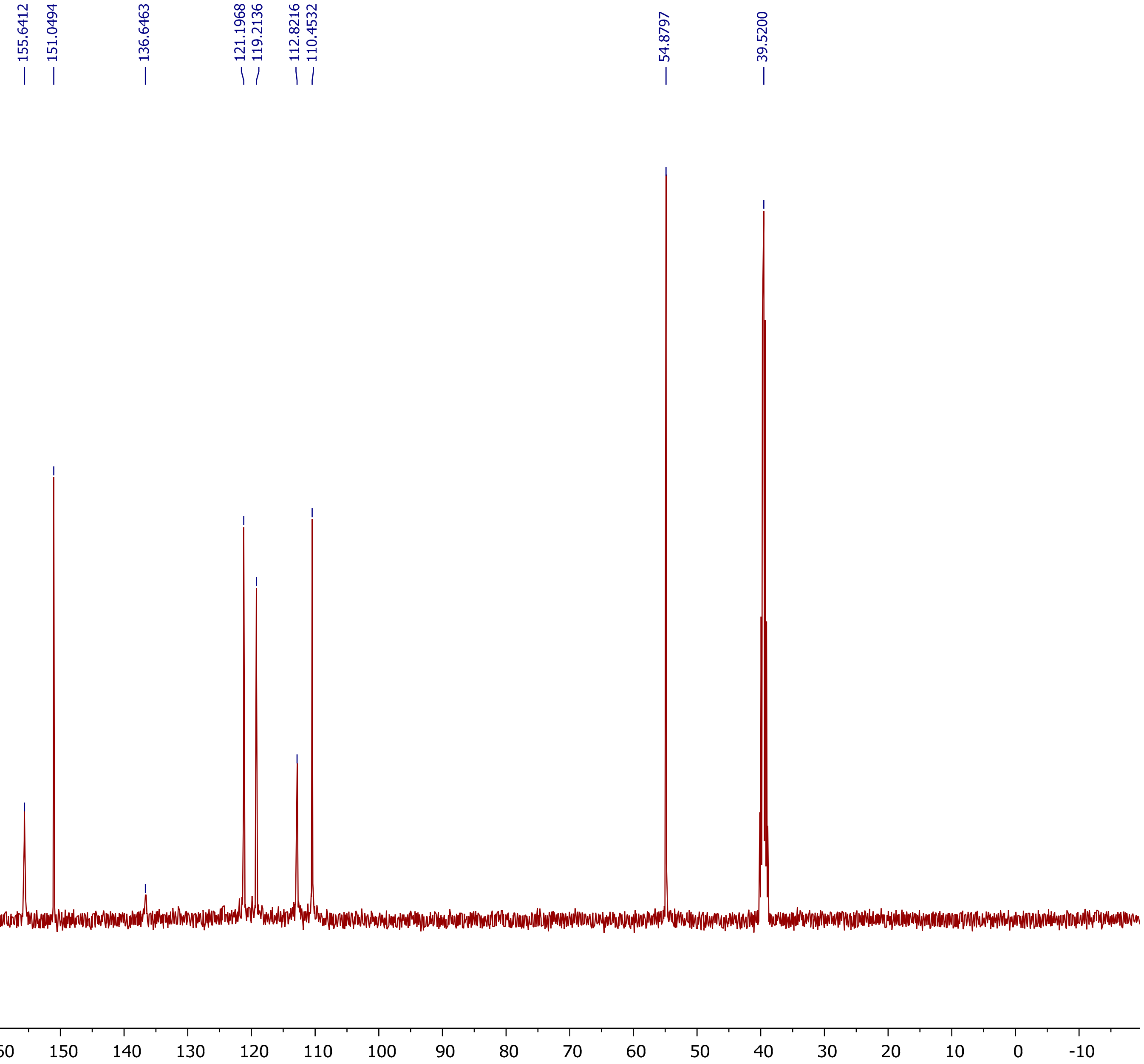

^{1}H NMR (400 MHz, DMSO-d6) **2h**

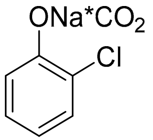

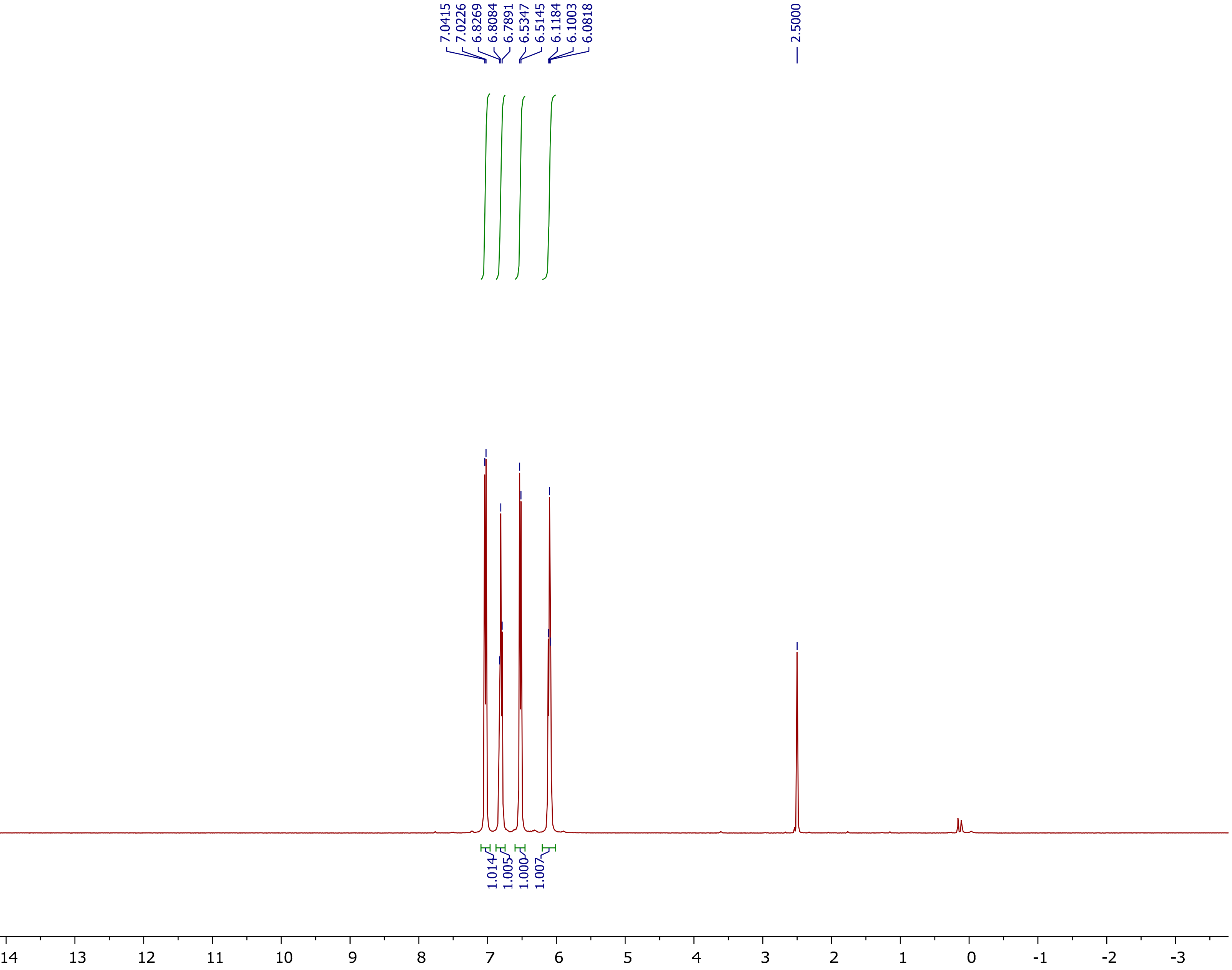

^{13}C NMR (101 MHz, DMSO-d$_6$) **2h**

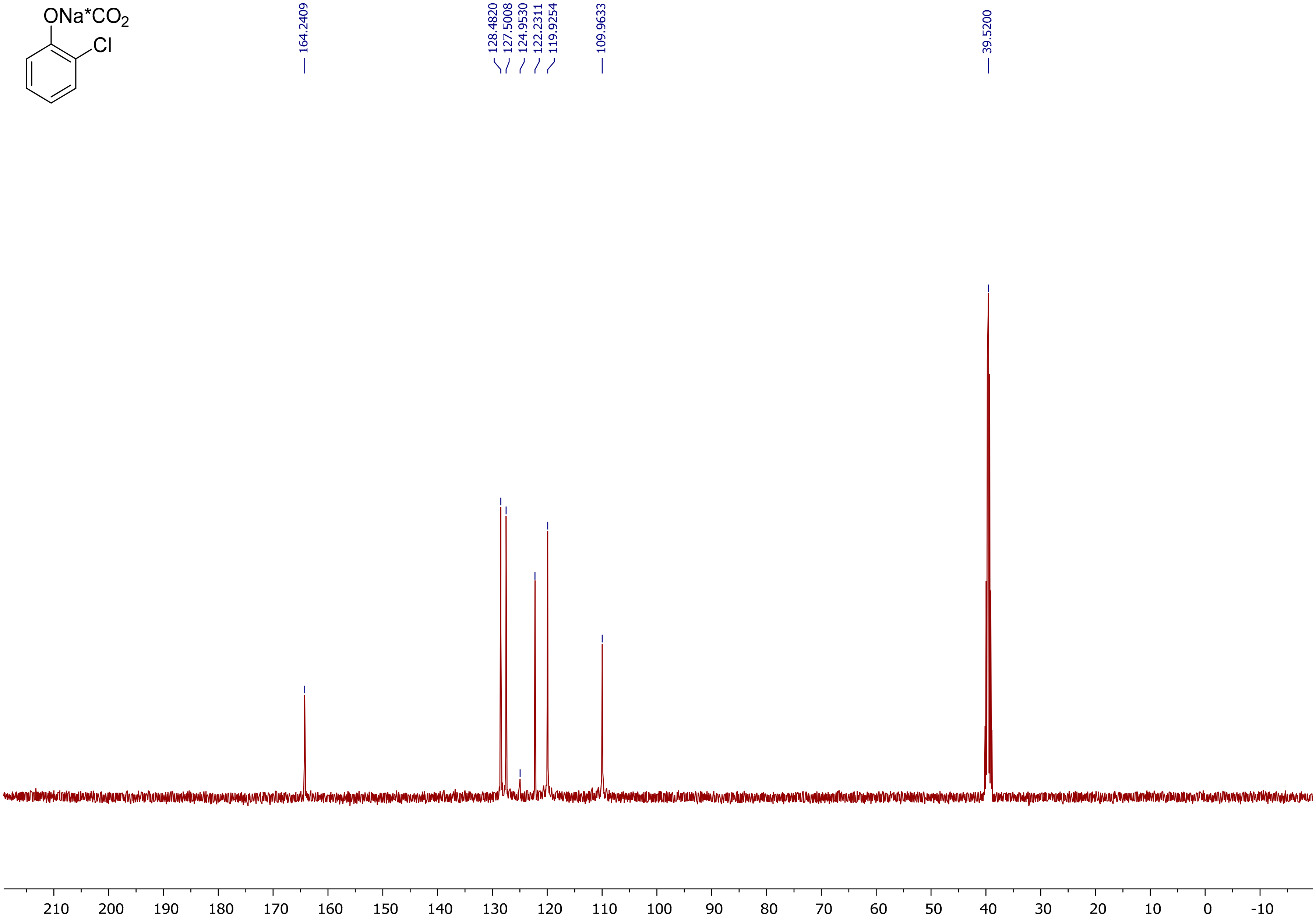

^{1}H NMR (400 MHz, DMSO-d6) **2i**

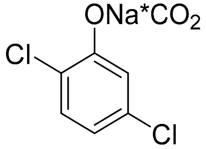

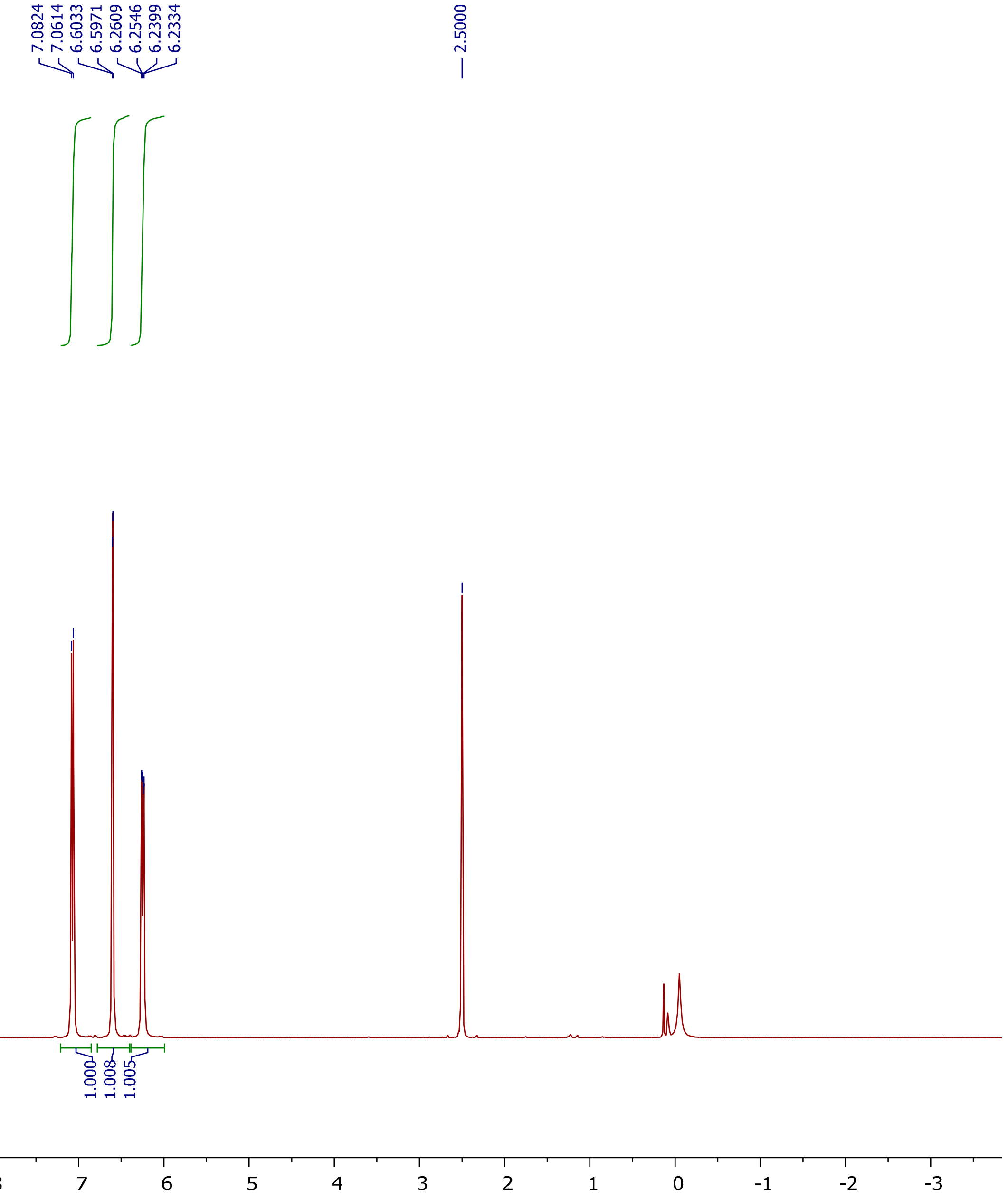

^{13}C NMR (101 MHz, DMSO-d$_6$) **2i**

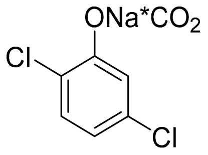

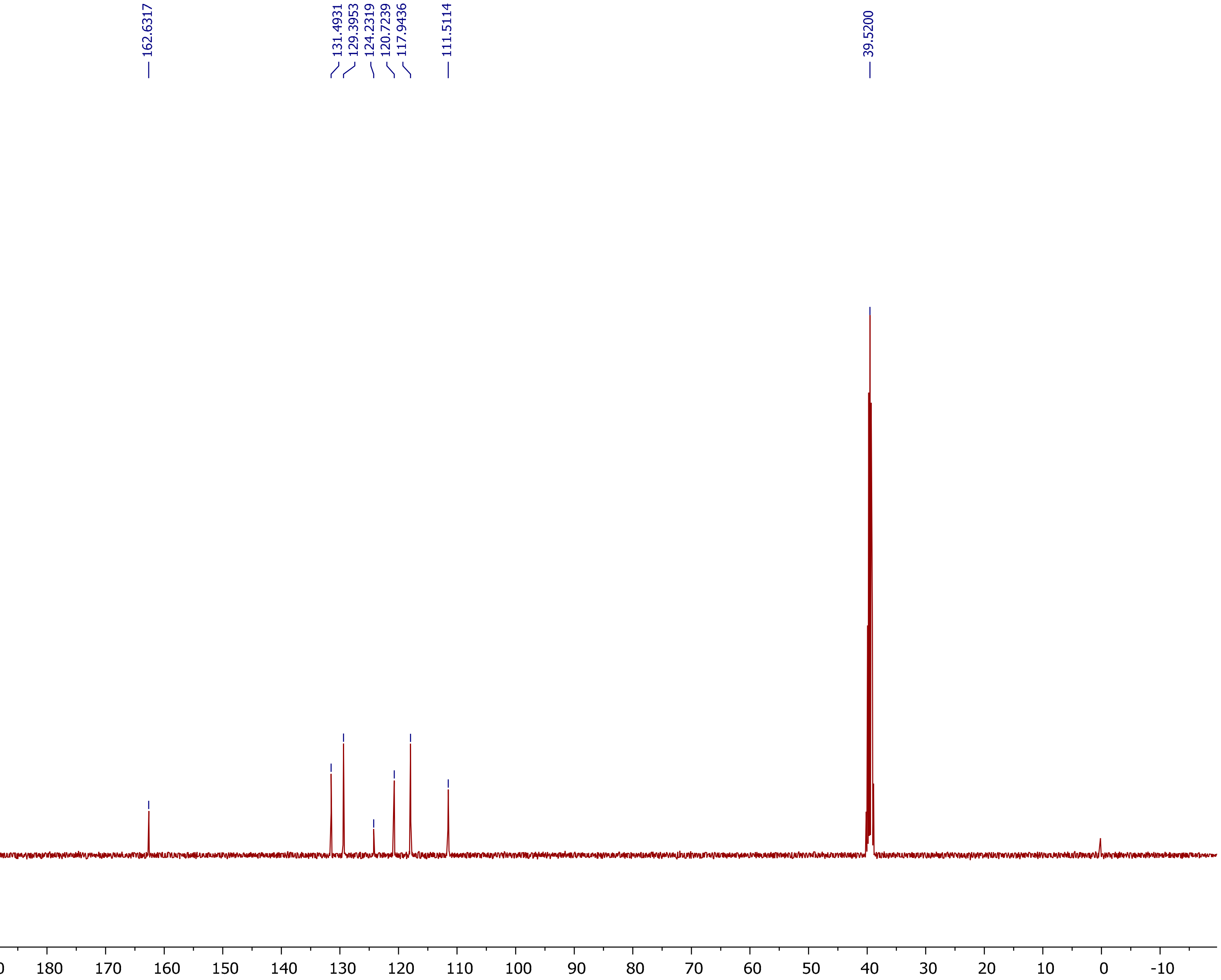

^{1}H NMR (400 MHz, DMSO-d6) **2j**

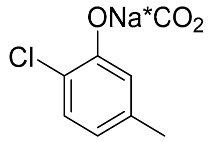

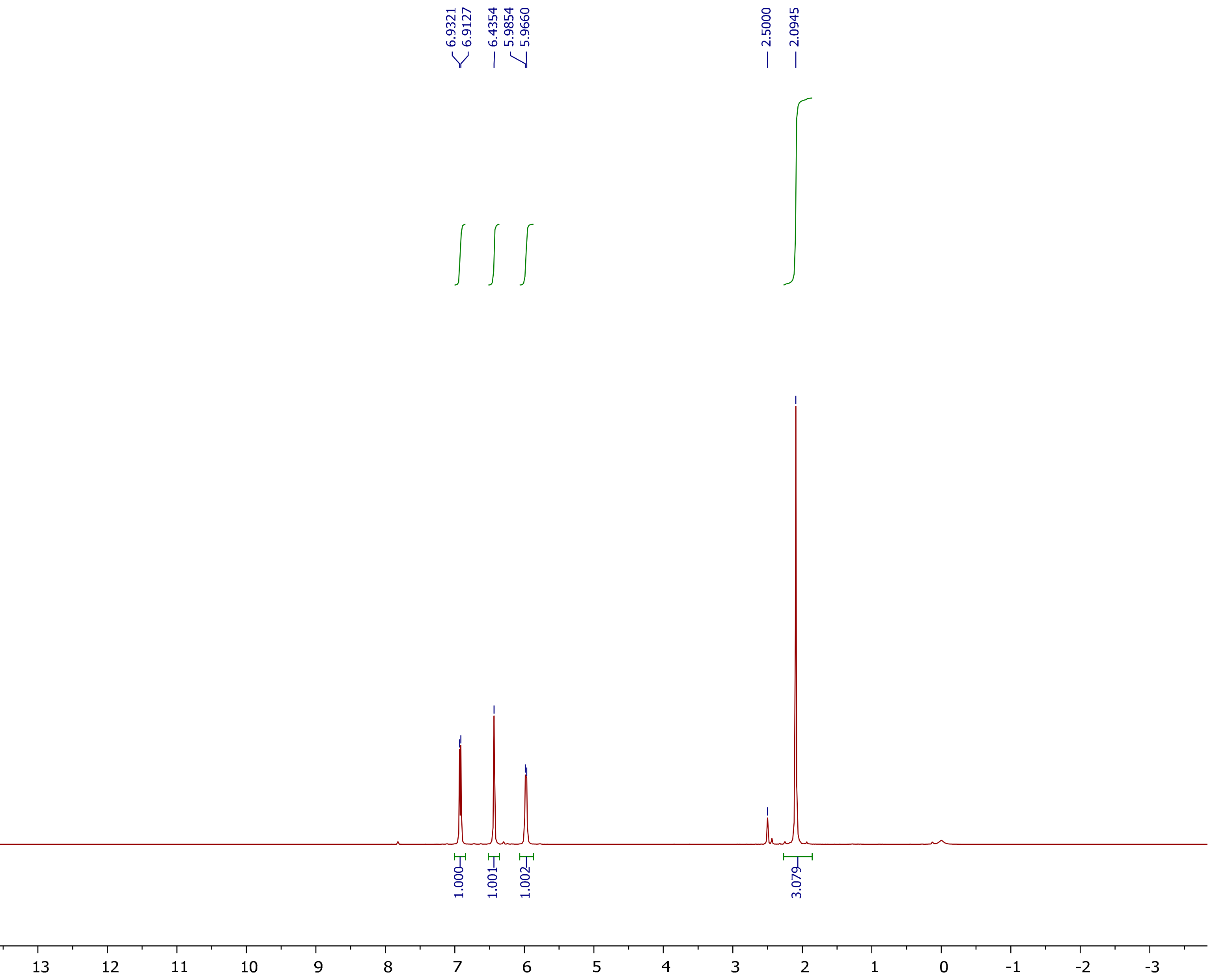

^{13}C NMR (101 MHz, DMSO-d6) **2j**

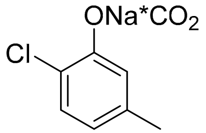

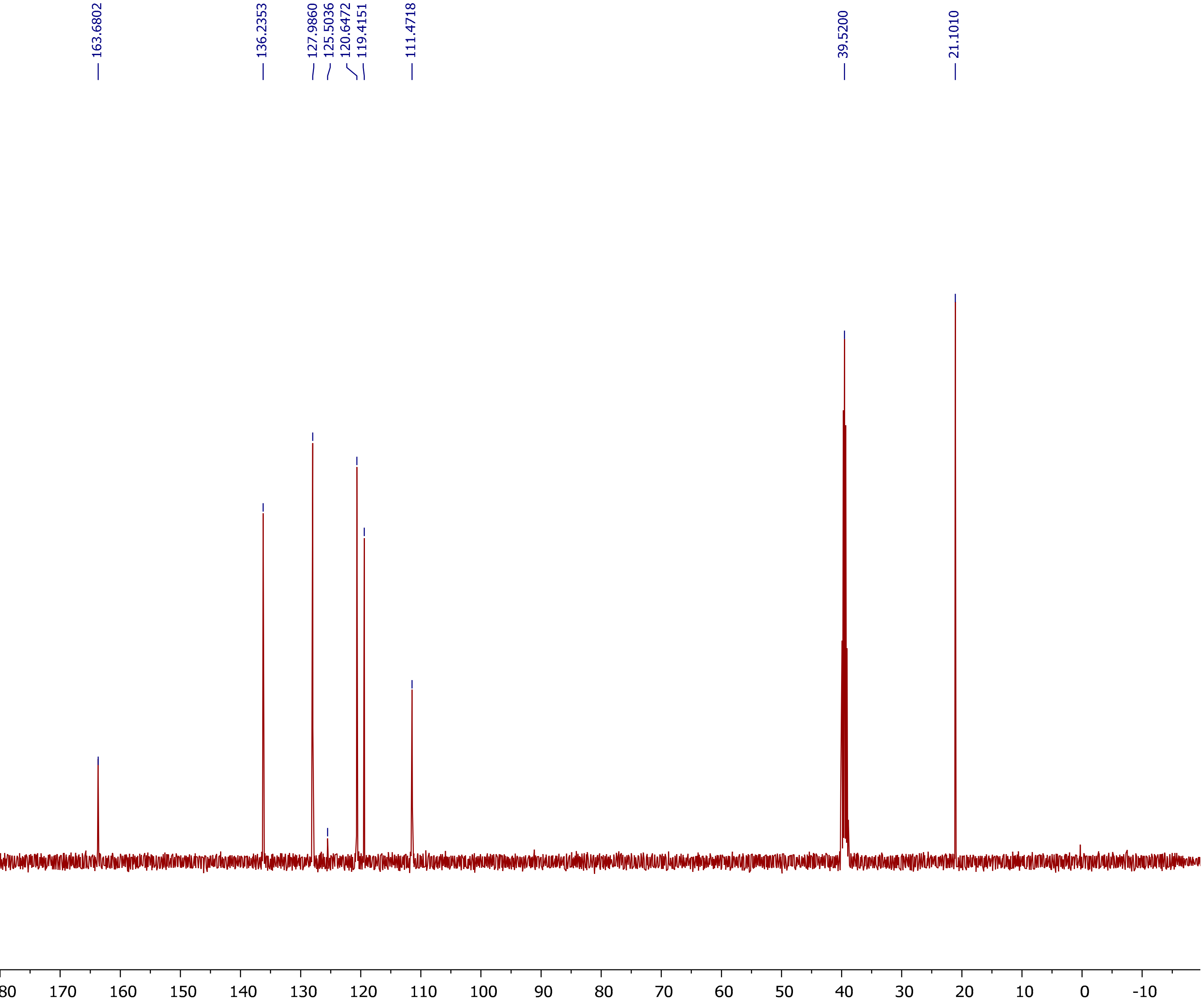

^{1}H NMR (400 MHz, DMSO-d6) **2k**

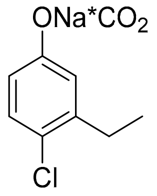

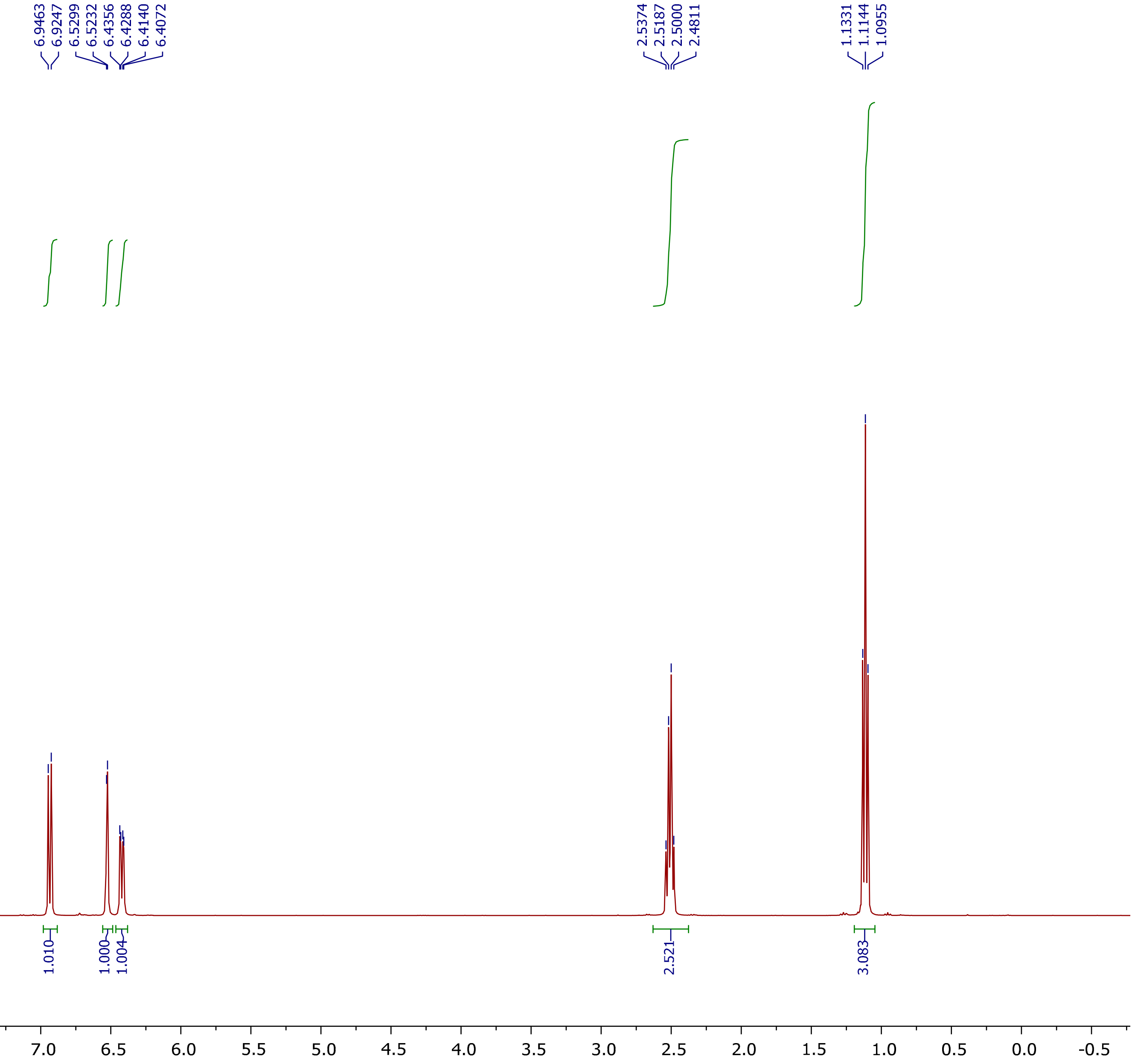

^{13}C NMR (101 MHz, DMSO-d$_6$) **2k**

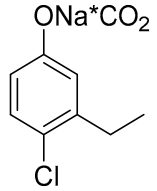

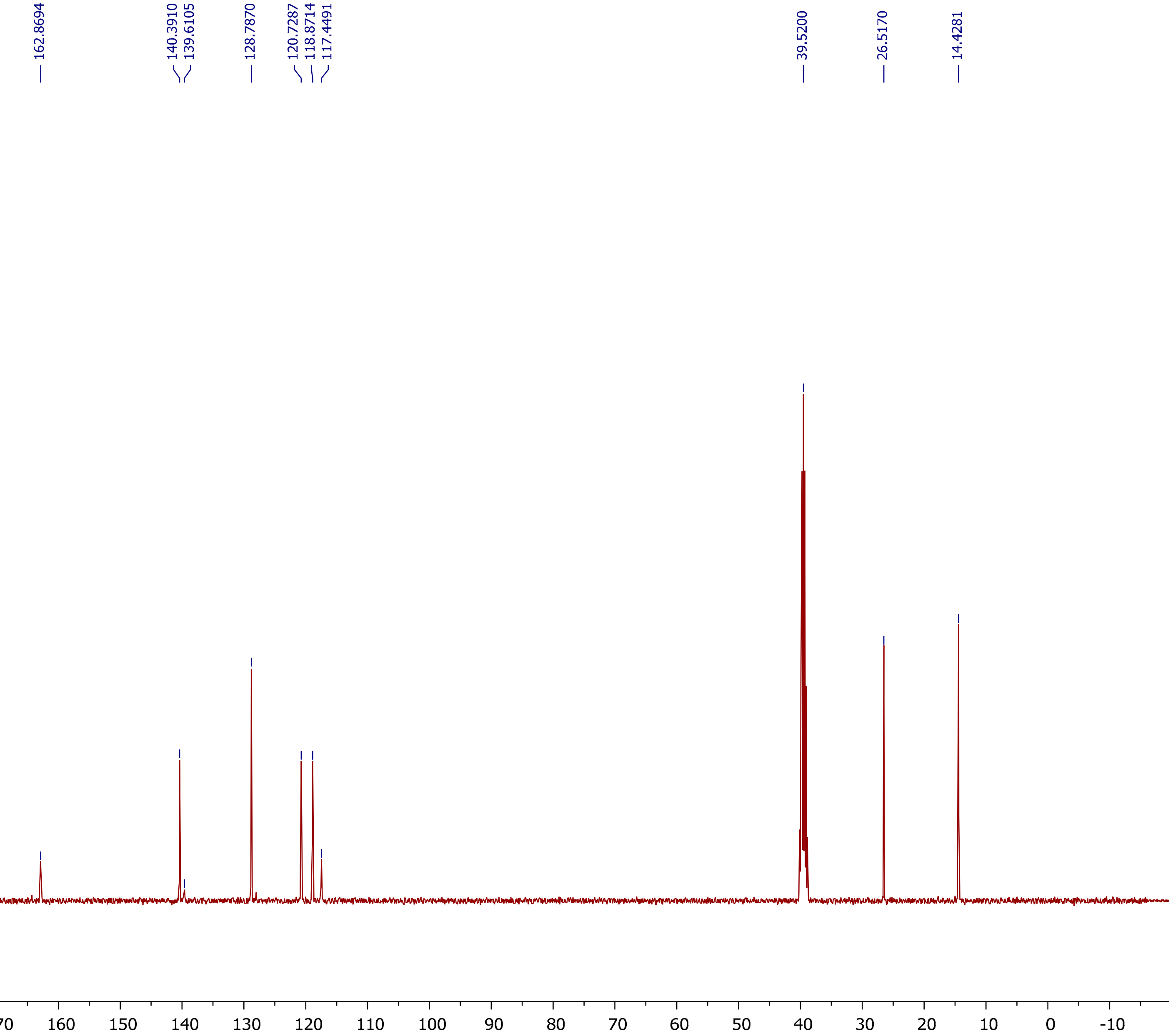

^{1}H NMR (400 MHz, DMSO-d6) **2o**

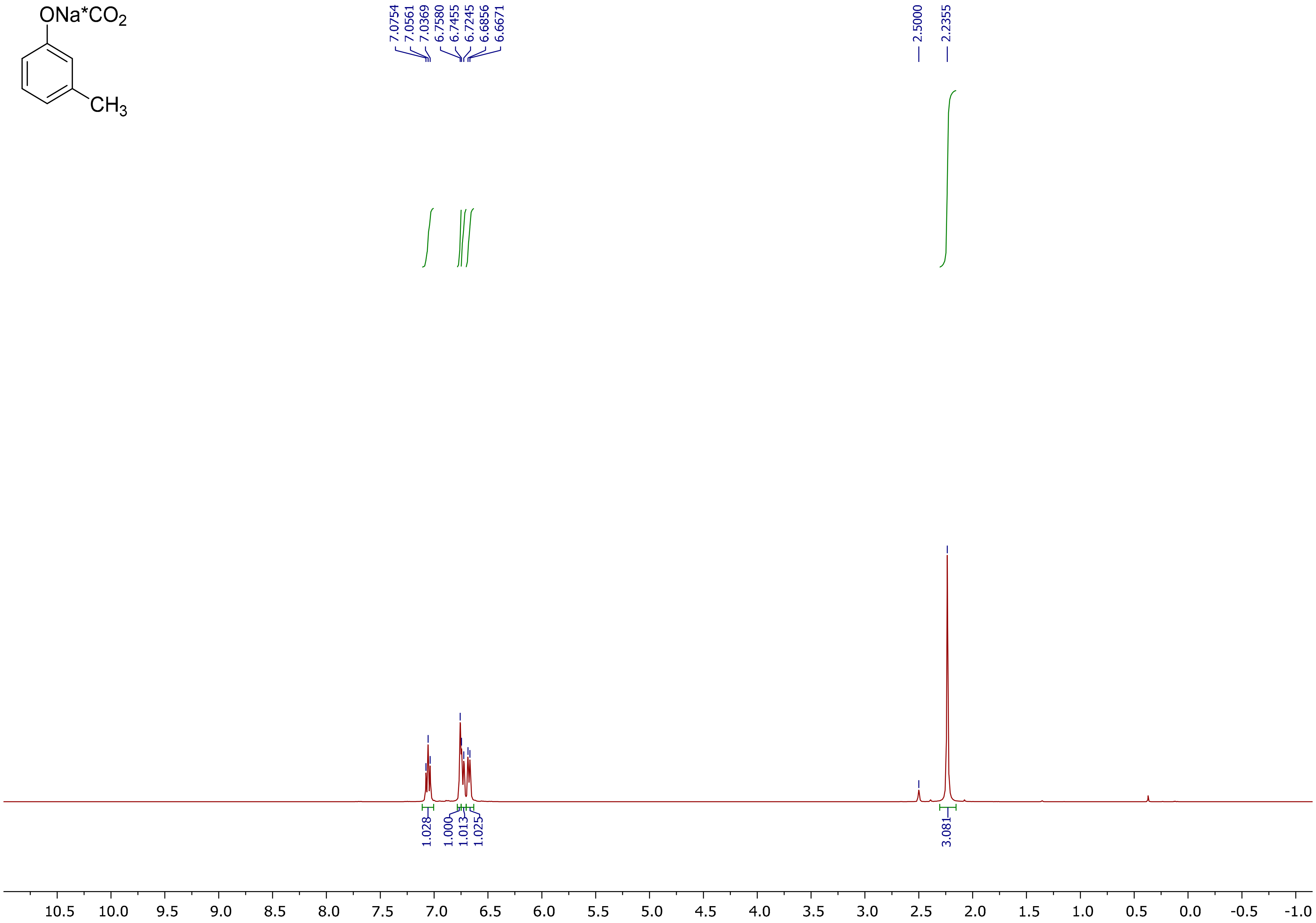

^{13}C NMR (101 MHz, DMSO-d6) **2o**

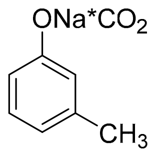

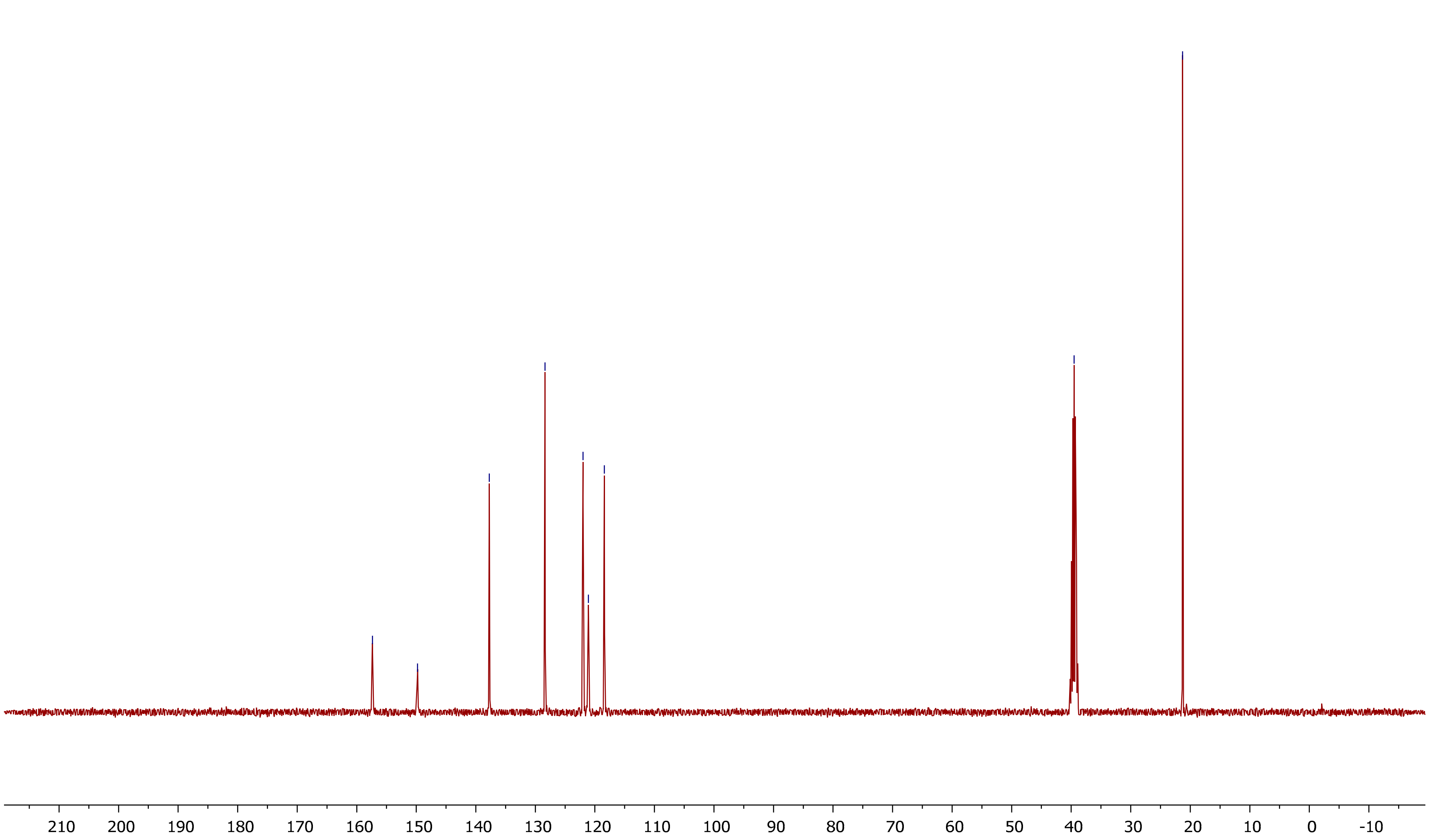

^{1}H NMR (400 MHz, DMSO-d6) **3a**

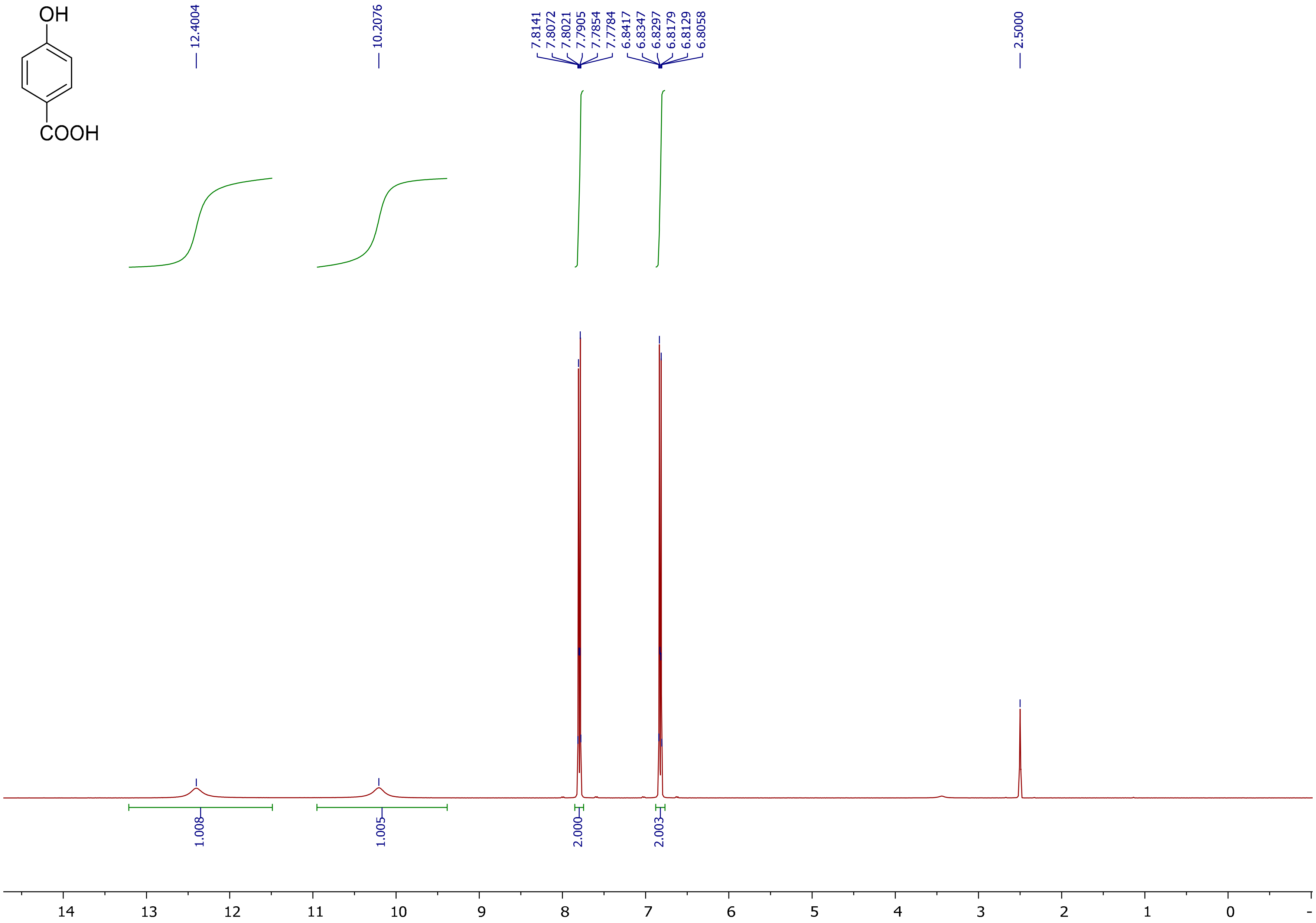

^{1}H NMR (400 MHz, DMSO-d6) **4c**

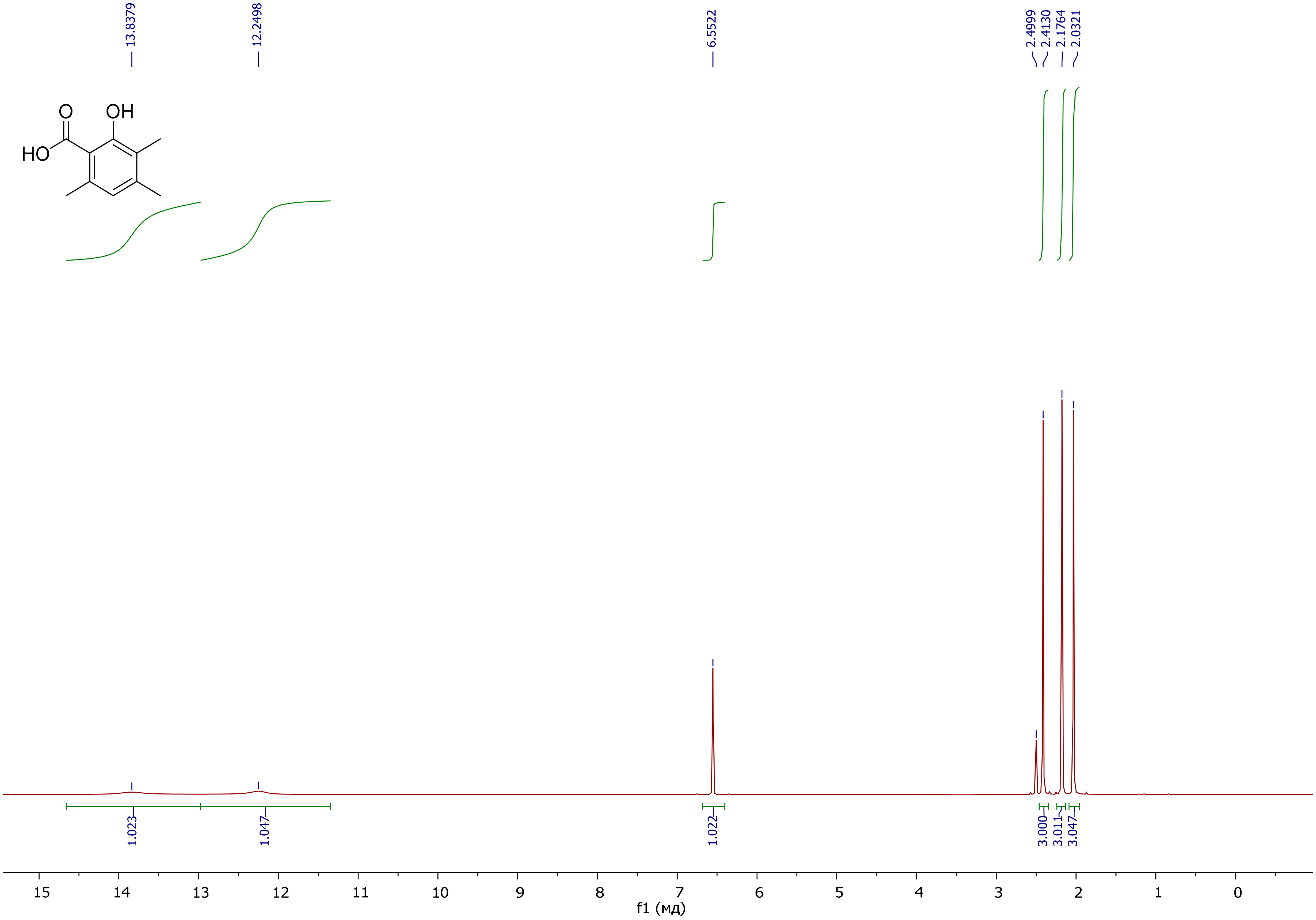

^{13}C NMR (101 MHz, DMSO-d$_6$) **4c**

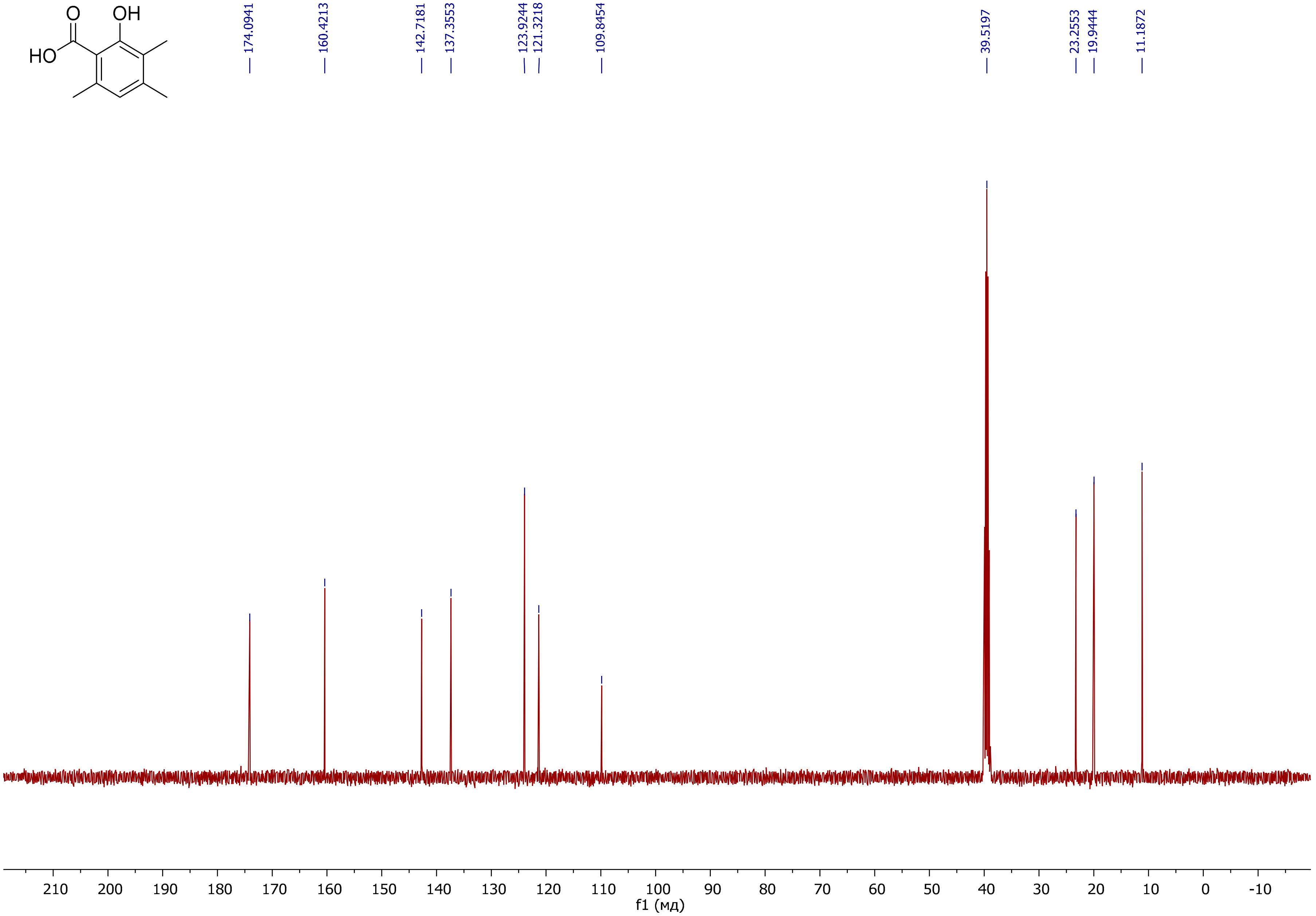

^{1}H NMR (400 MHz, DMSO-d6) **4d**

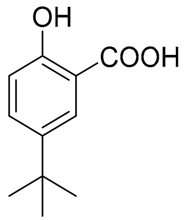

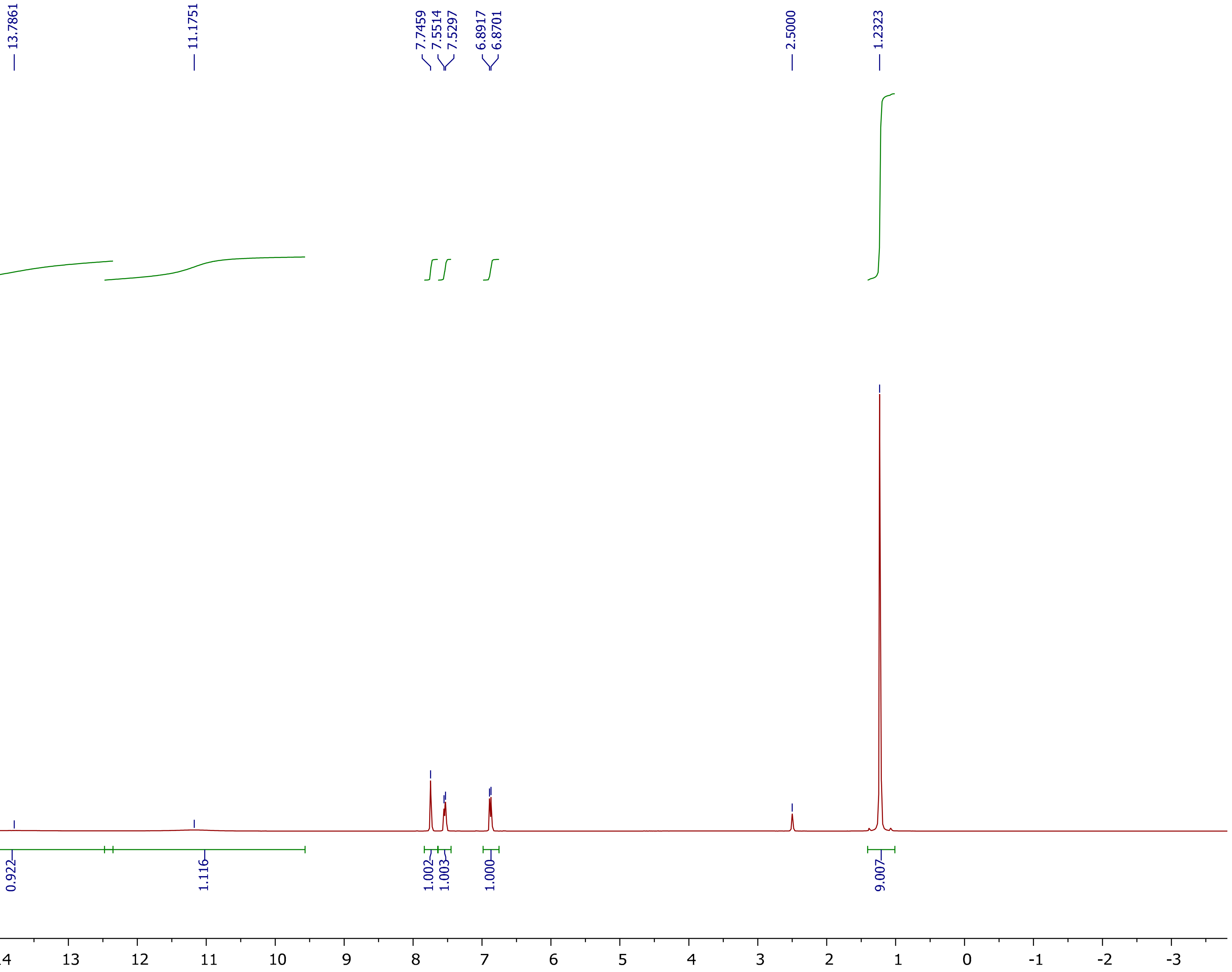

^{13}C NMR (101 MHz, DMSO-d$_6$) **4d**

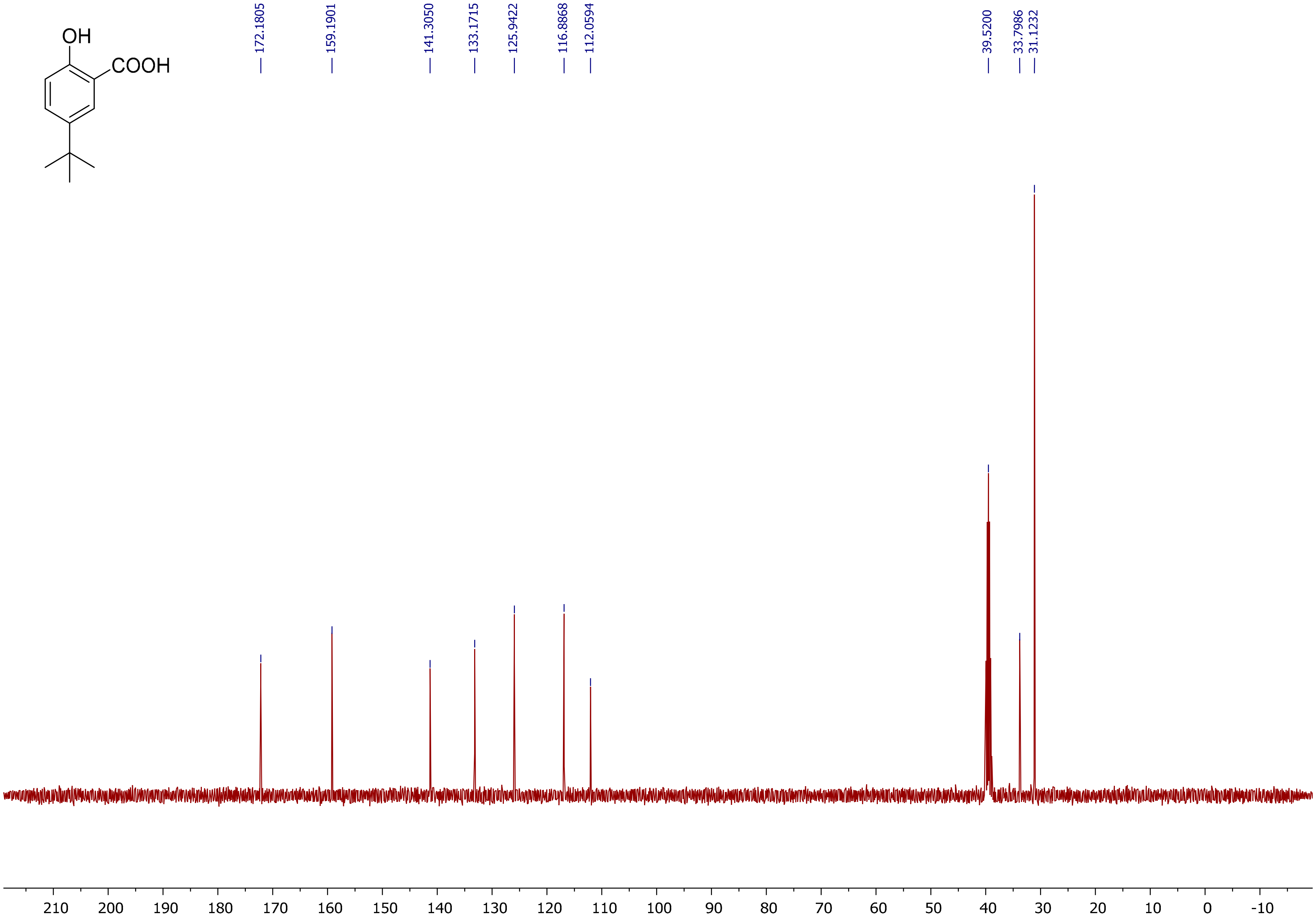

^{1}H NMR (400 MHz, DMSO-d_6) **4e**

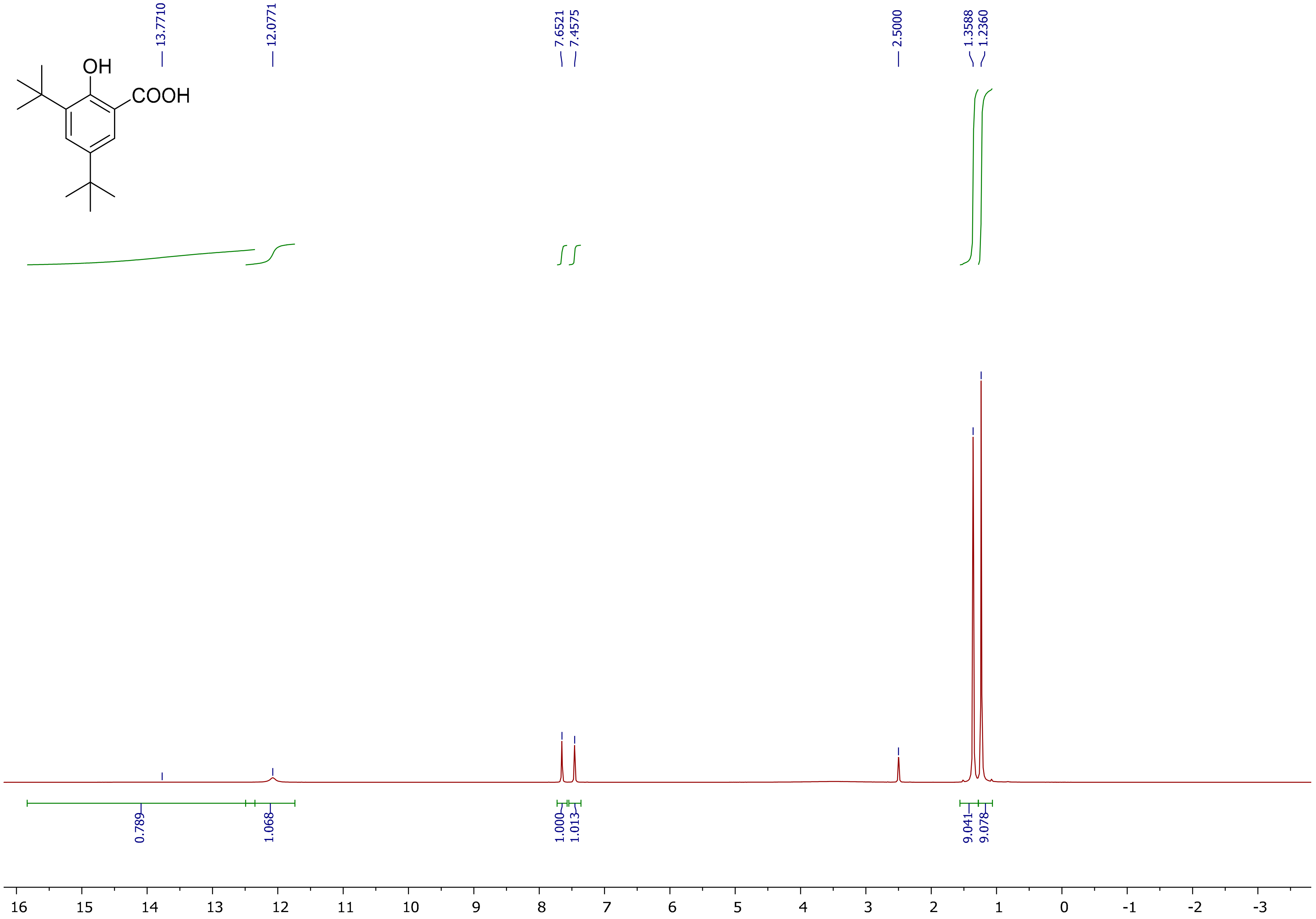

^{13}C NMR (101 MHz, DMSO-d$_6$) **4e**

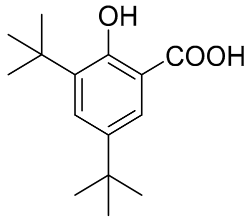

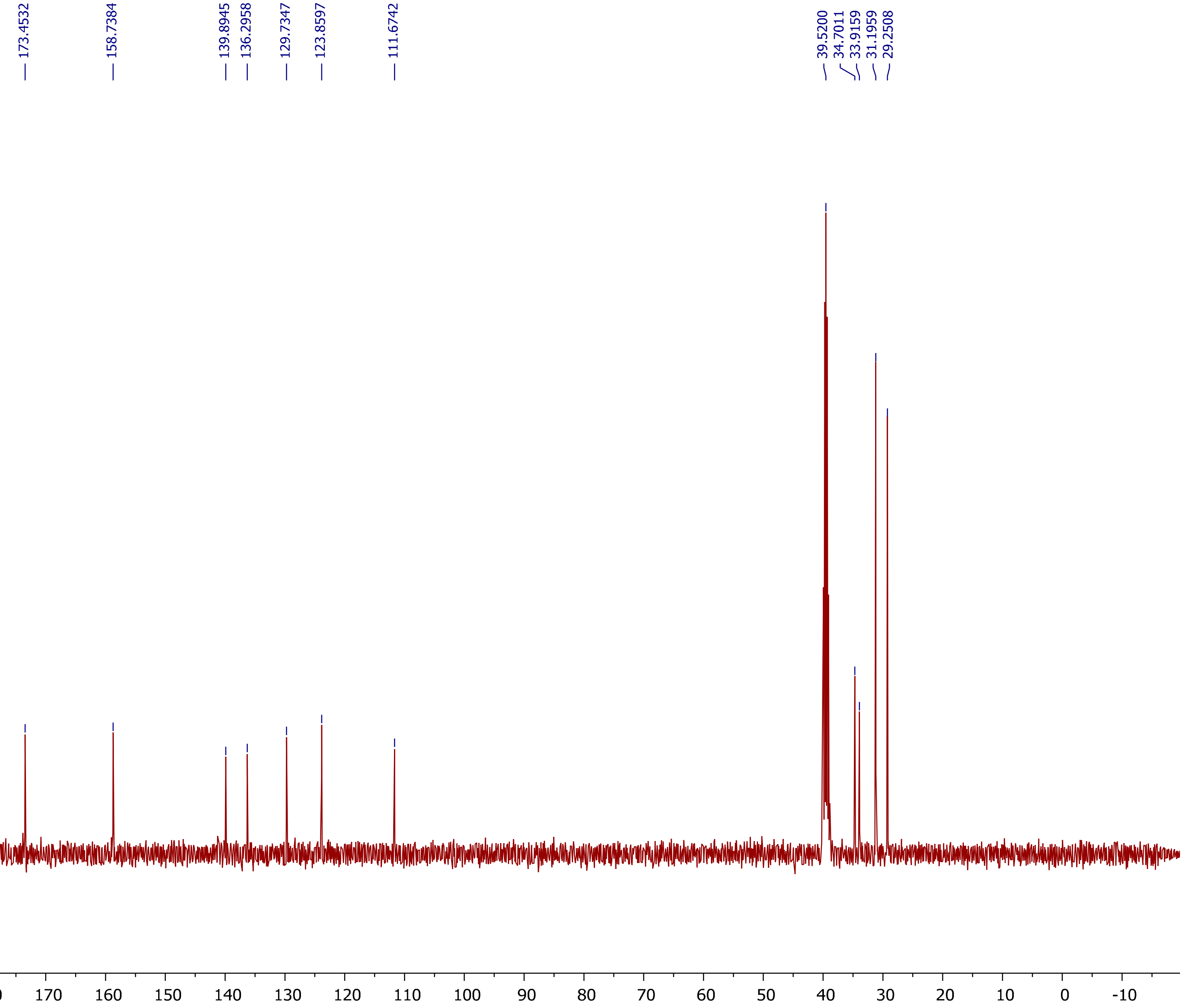

^1H NMR (400 MHz, DMSO-d_6) **3f**

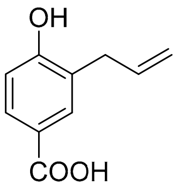

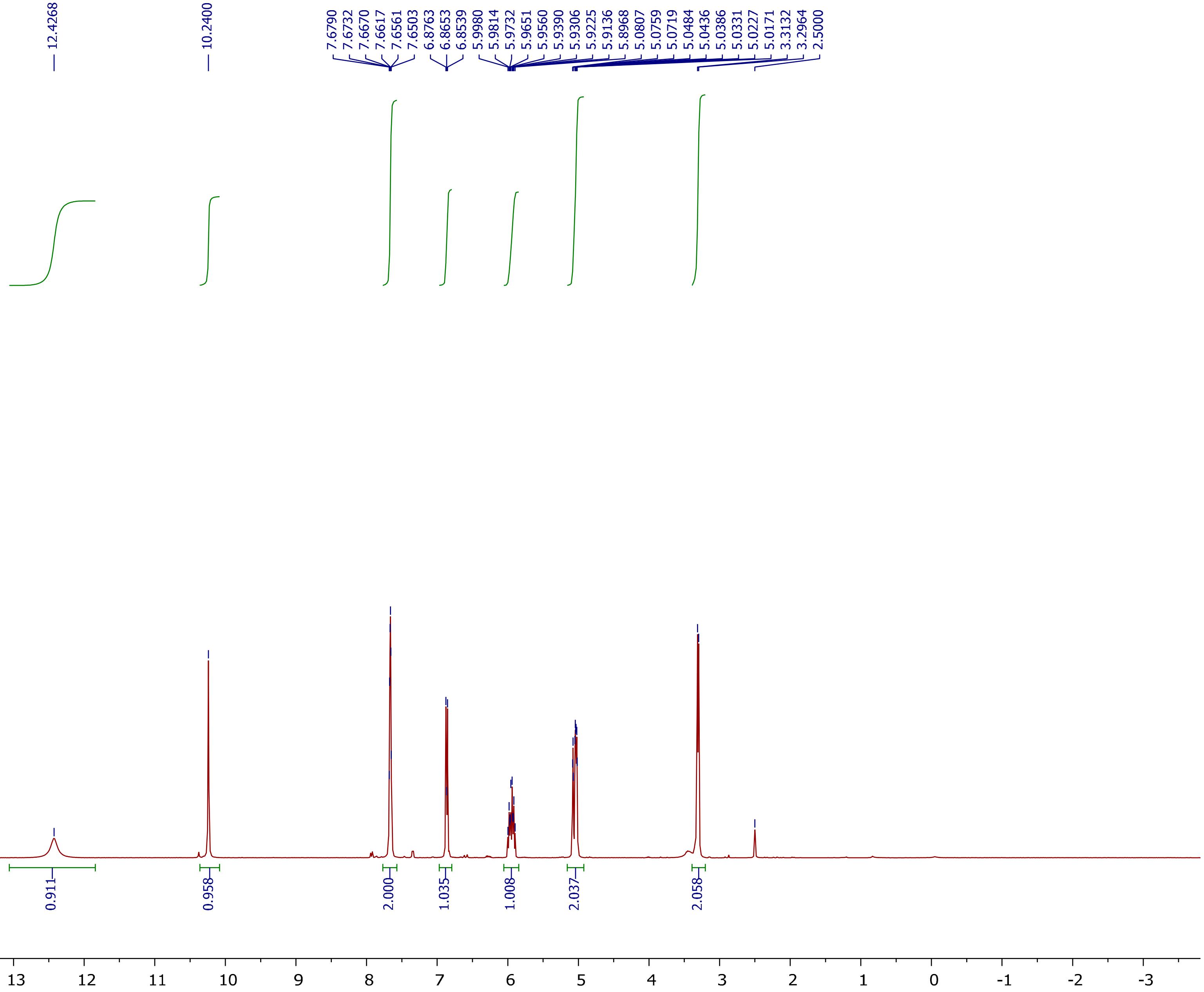

^{13}C NMR (101 MHz, DMSO-d$_6$) **3f**

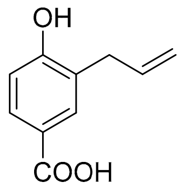

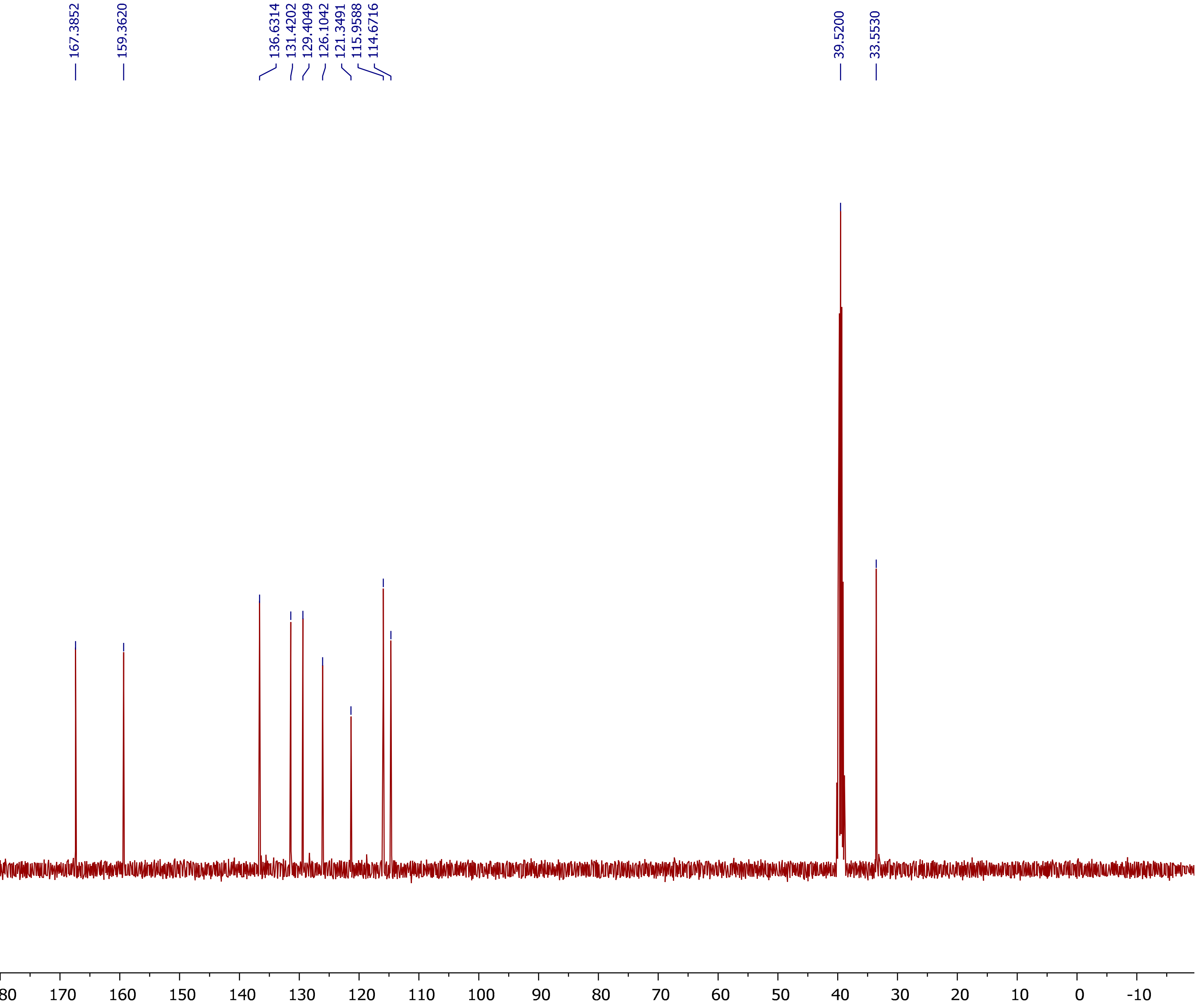

^{1}H NMR (400 MHz, DMSO-d6) **3g**

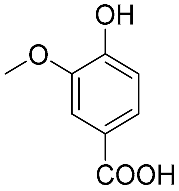

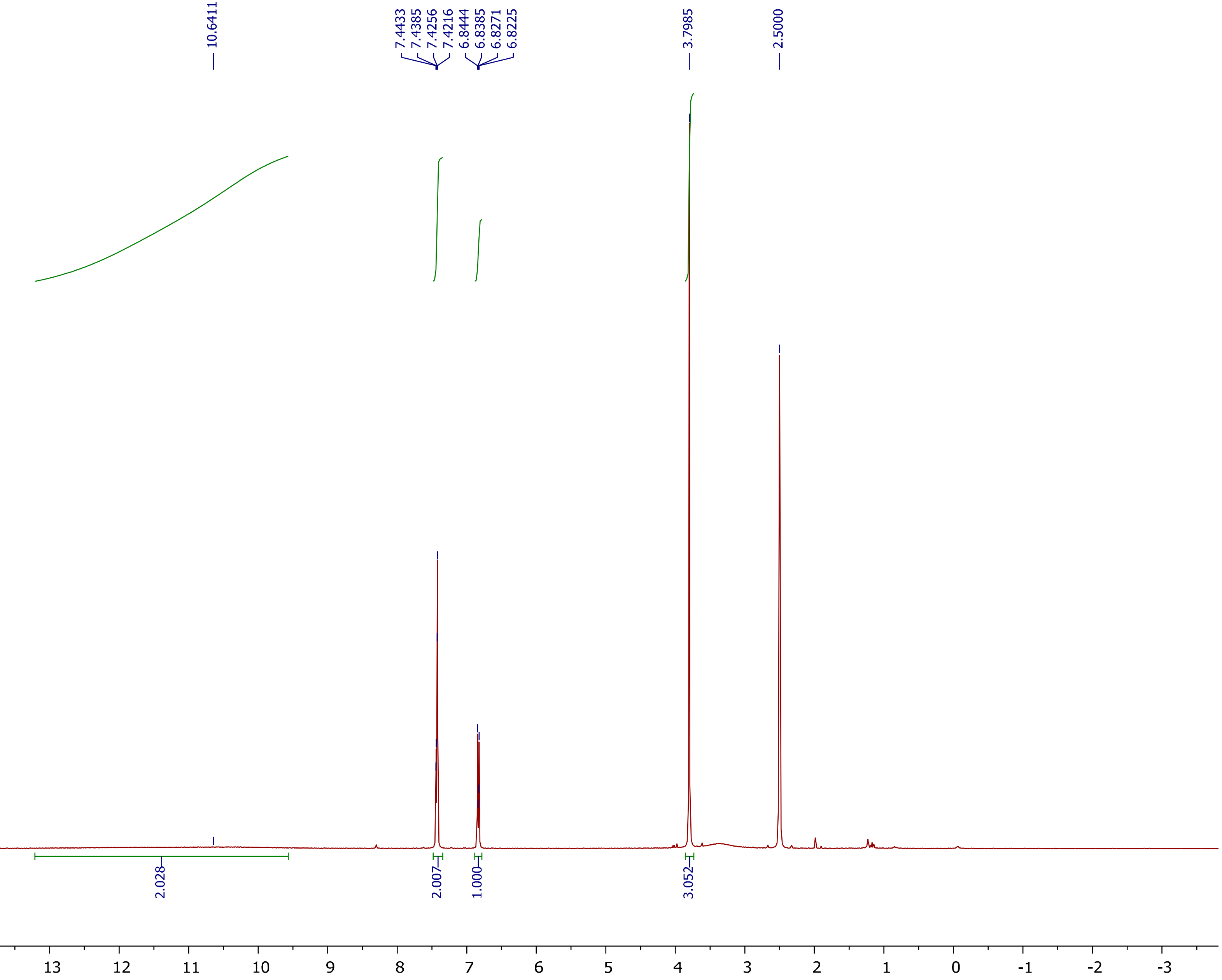

^1H NMR (400 MHz, DMSO-d_6) **3h**

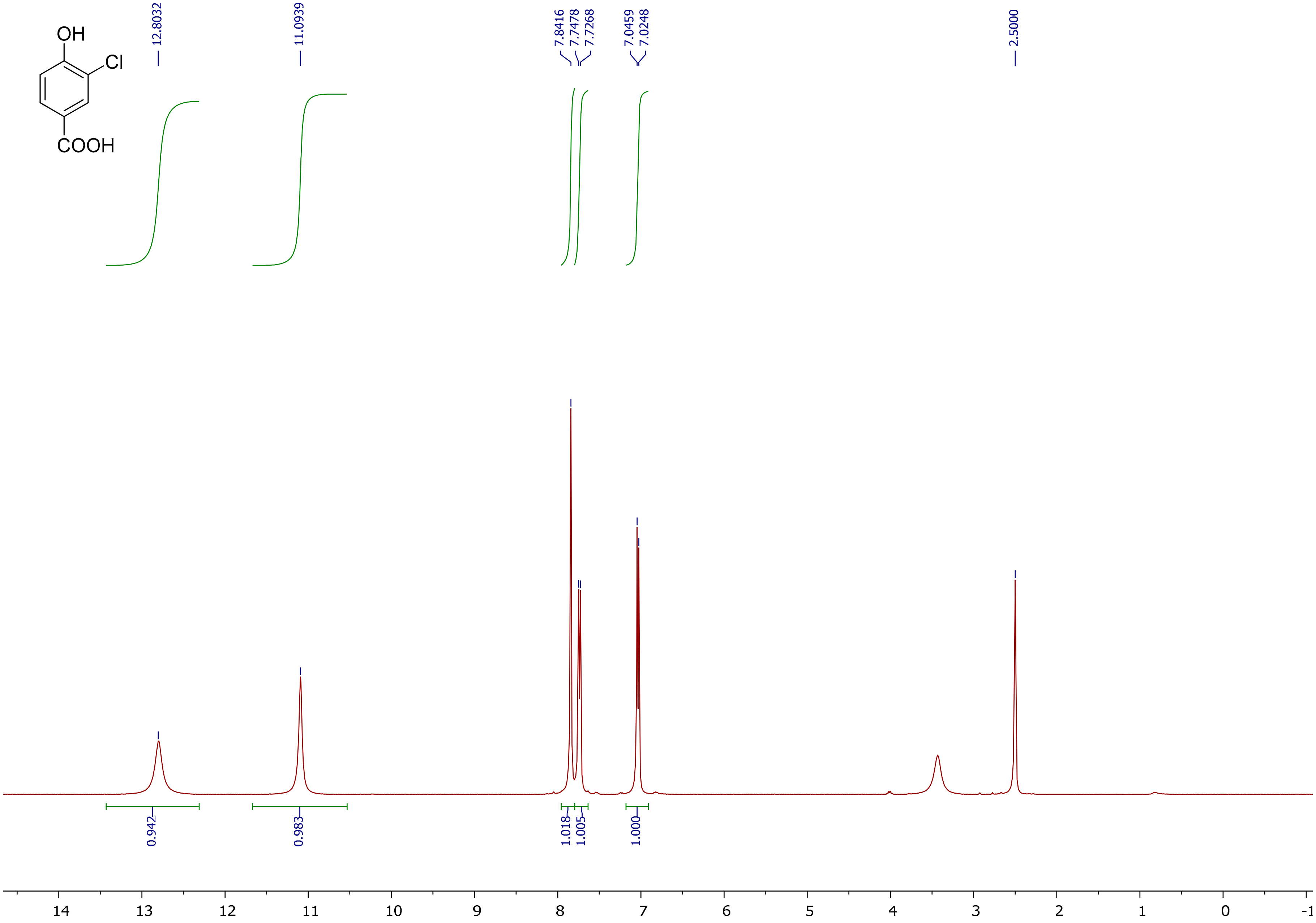

^{13}C NMR (101 MHz, DMSO-d_6) **3h**

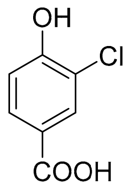

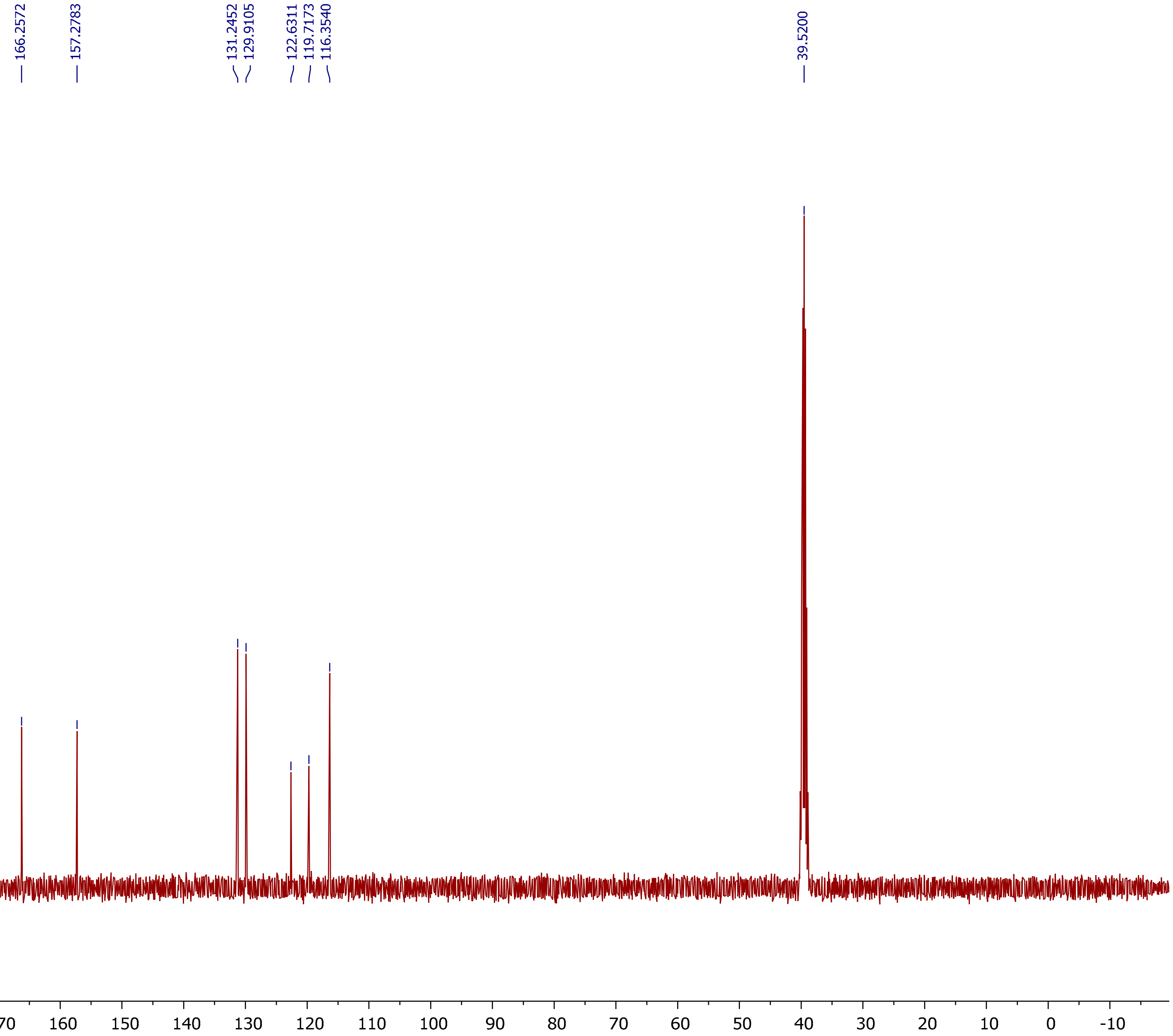

^{1}H NMR (400 MHz, DMSO-d6) **4k**

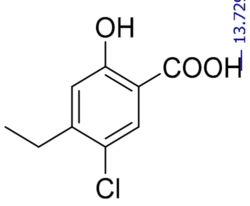

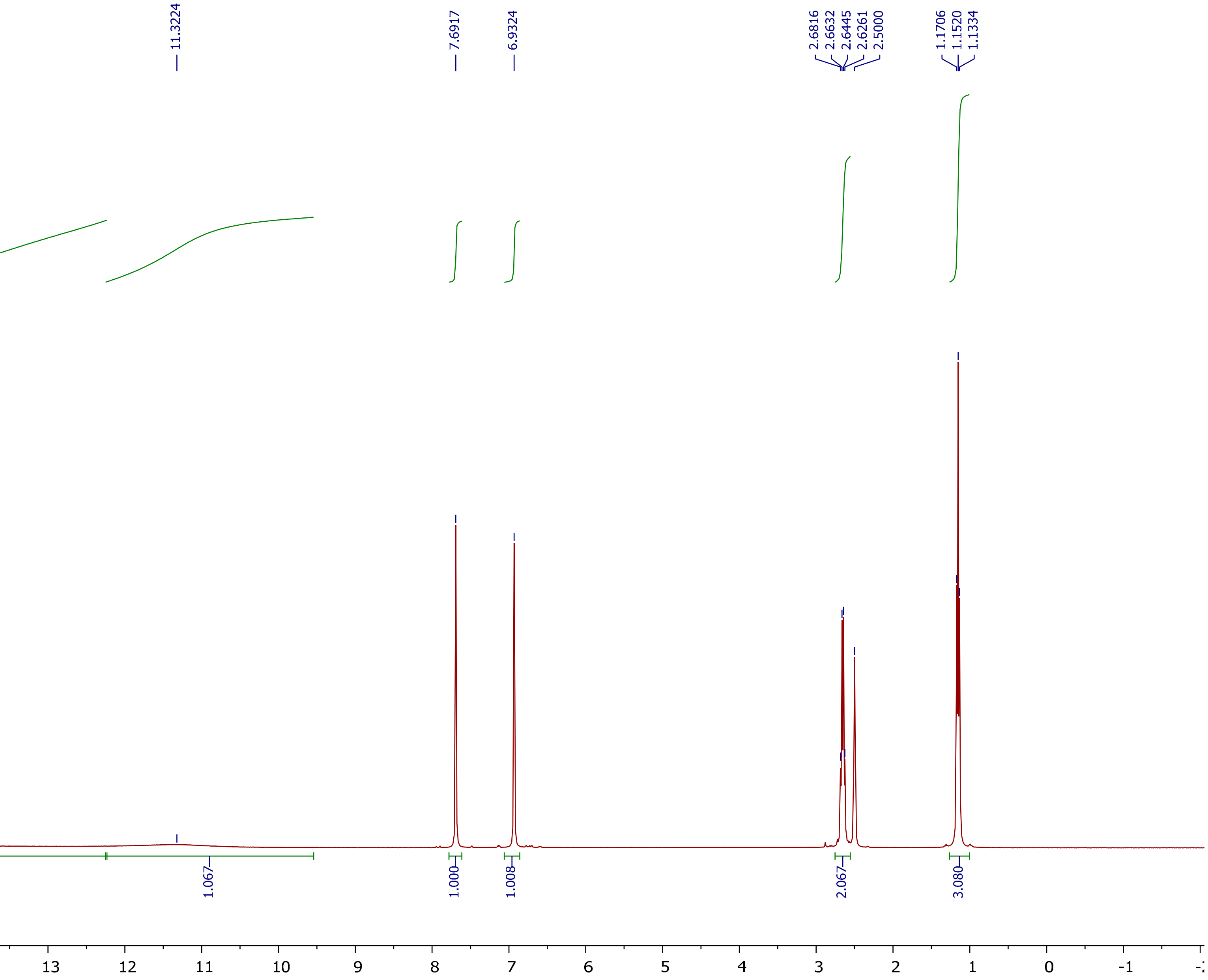

^{13}C NMR (101 MHz, DMSO-d_6) **4k**

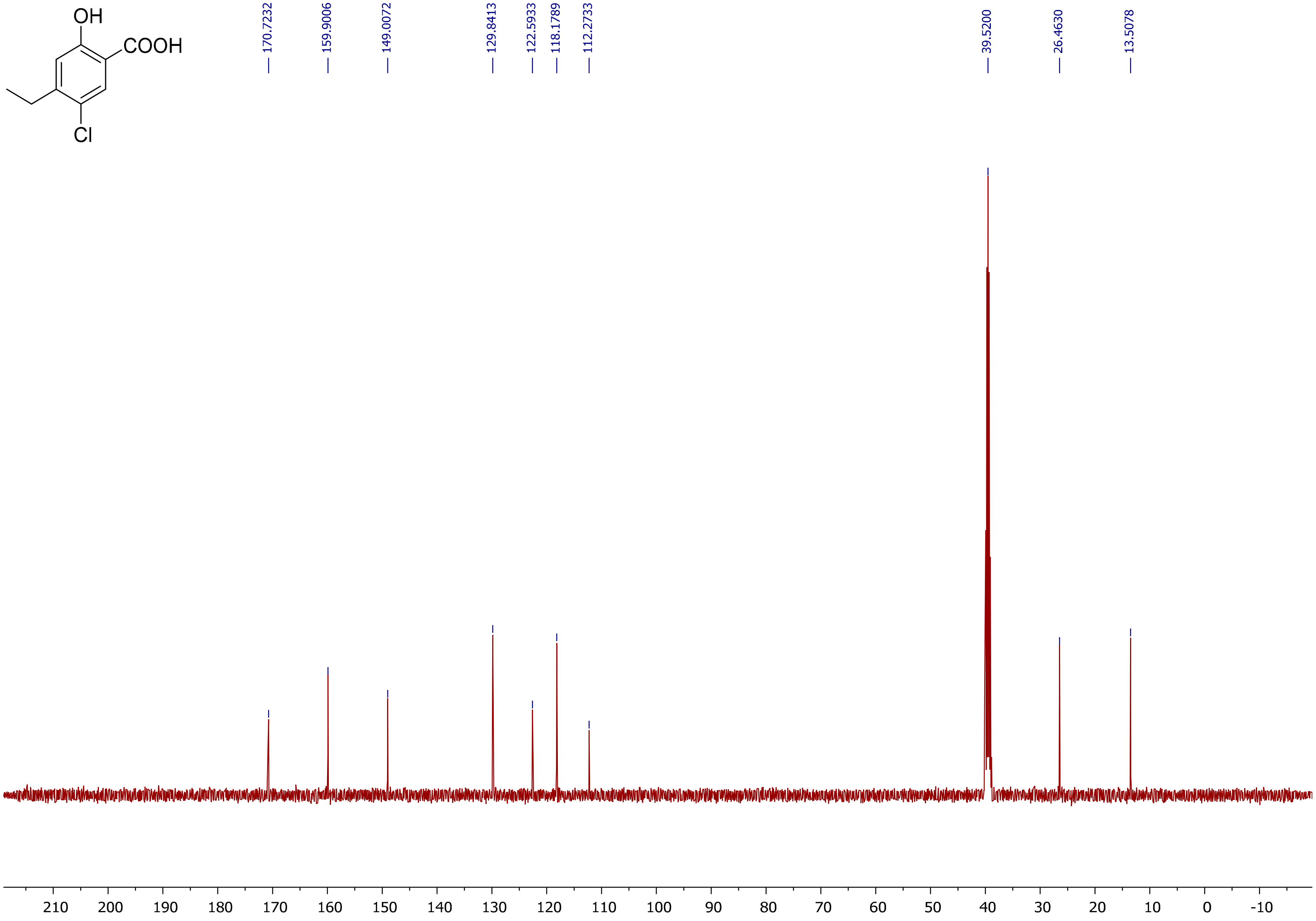

^{1}H NMR (400 MHz, DMSO-d6) **4l**

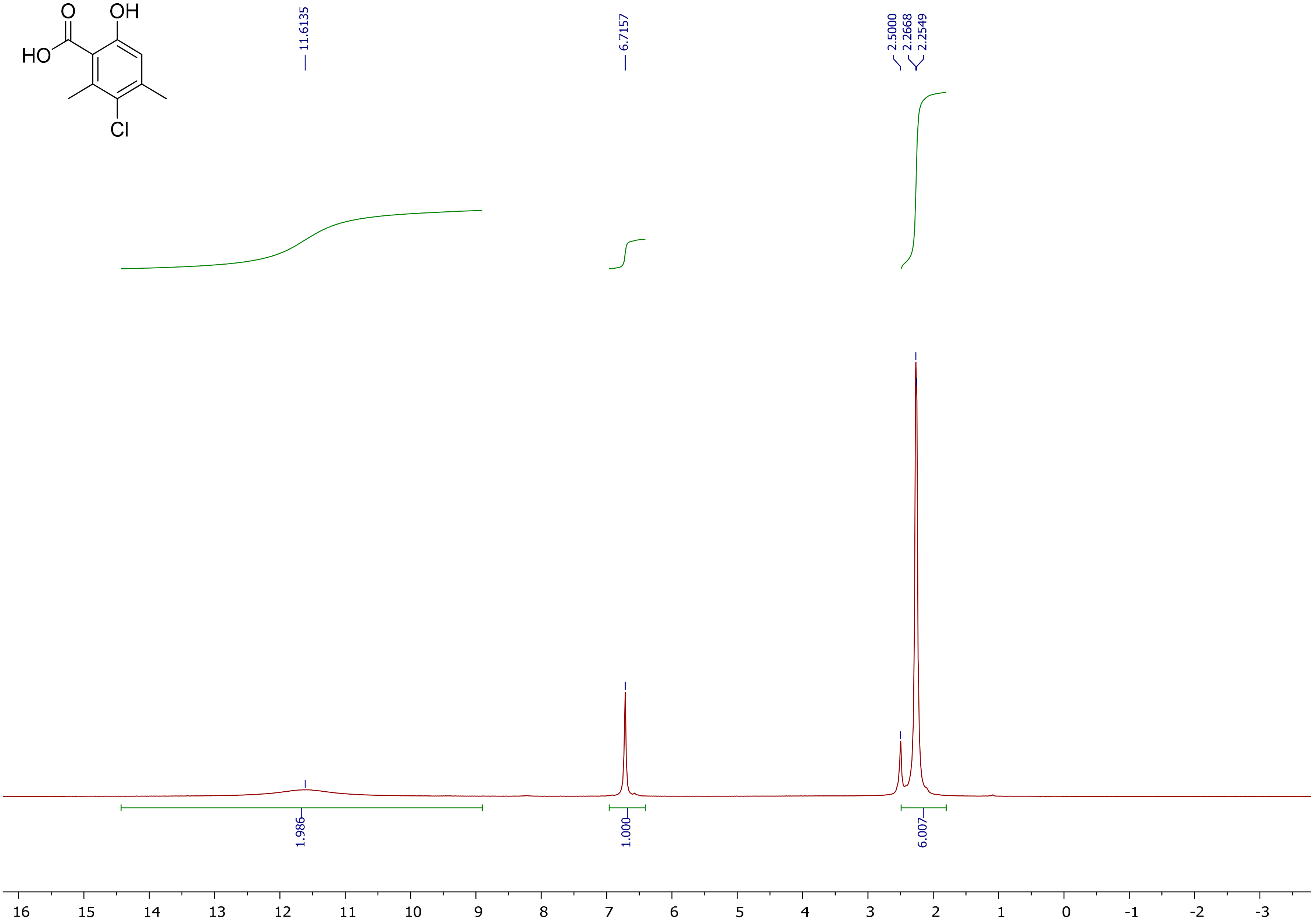

^{13}C NMR (101 MHz, DMSO-d$_6$) **4l**

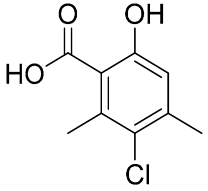

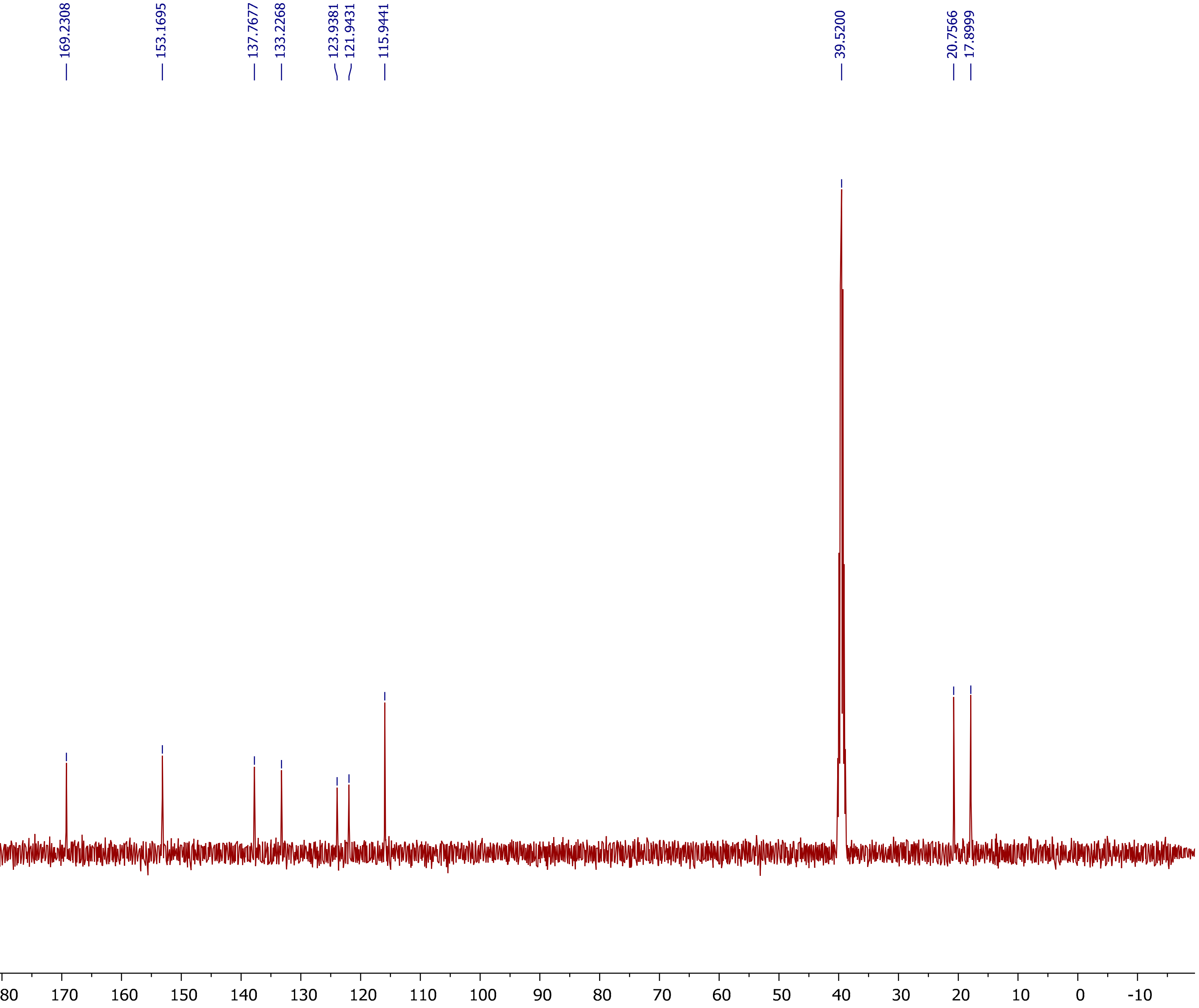

^{1}H NMR (400 MHz, DMSO-d_6) **3m**

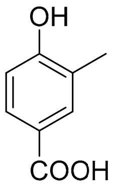

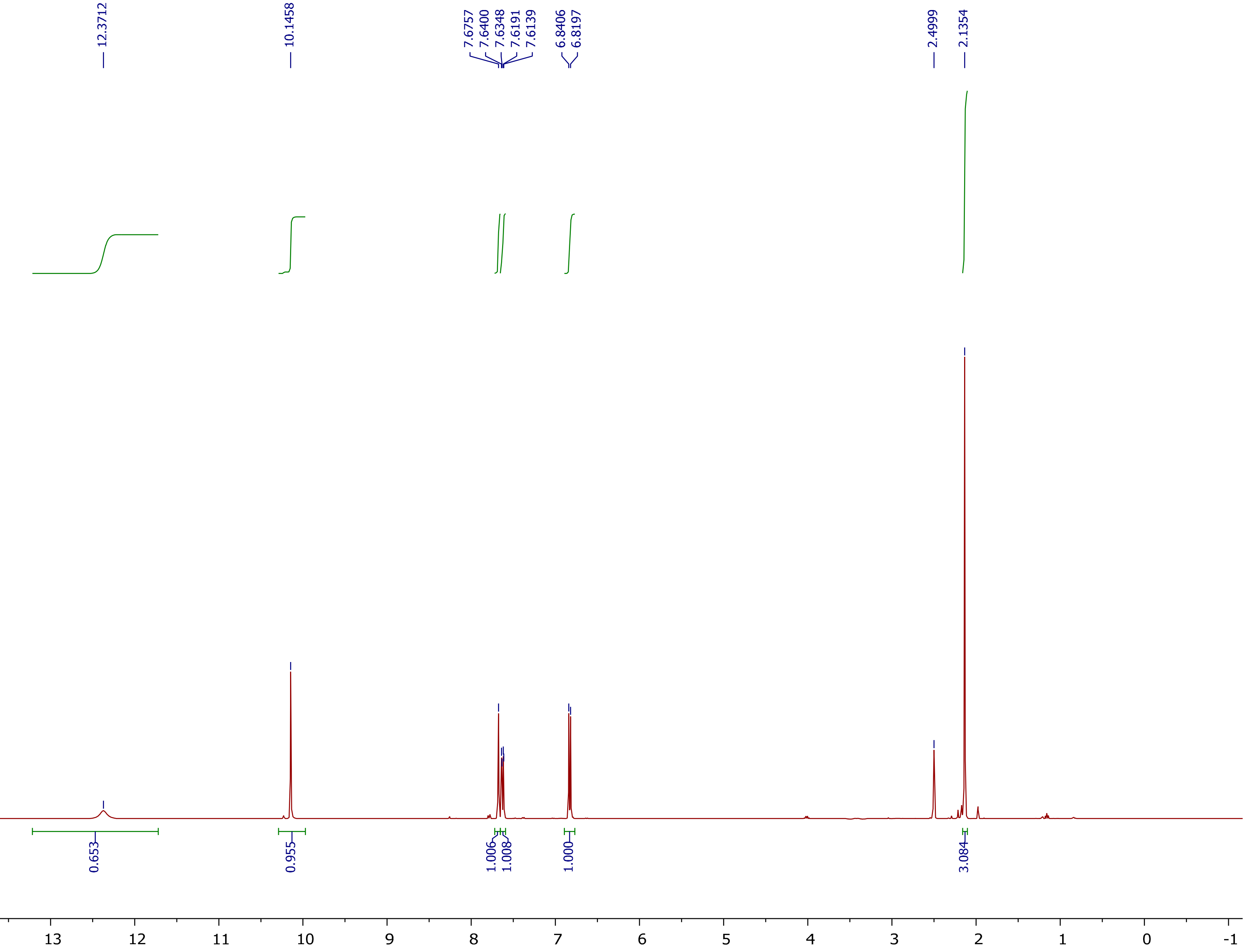

^{1}H NMR (400 MHz, DMSO-d6) **3n**

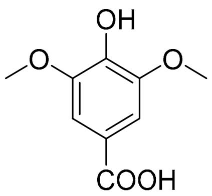

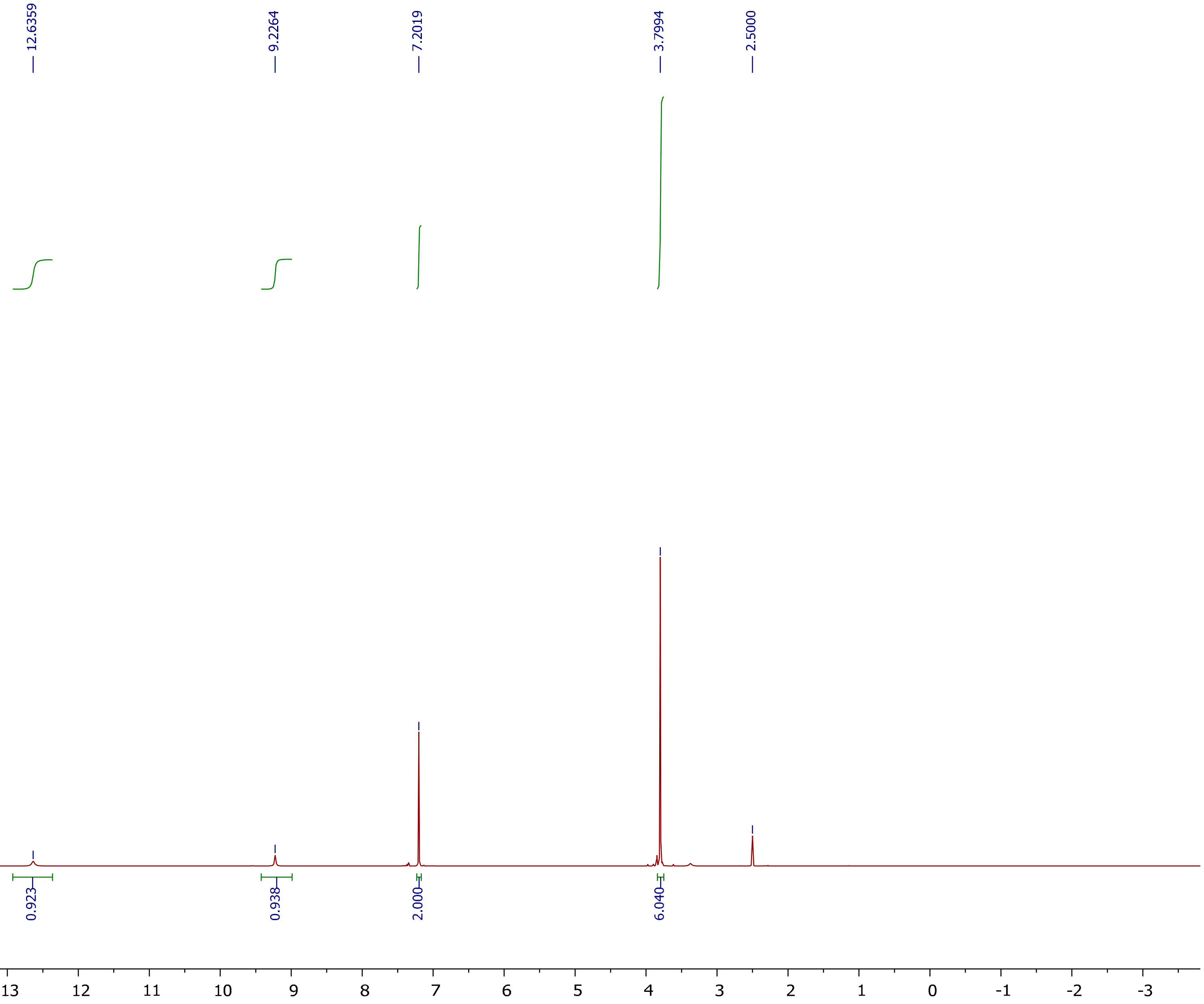

^{1}H NMR (400 MHz, DMSO-d6) **4o**

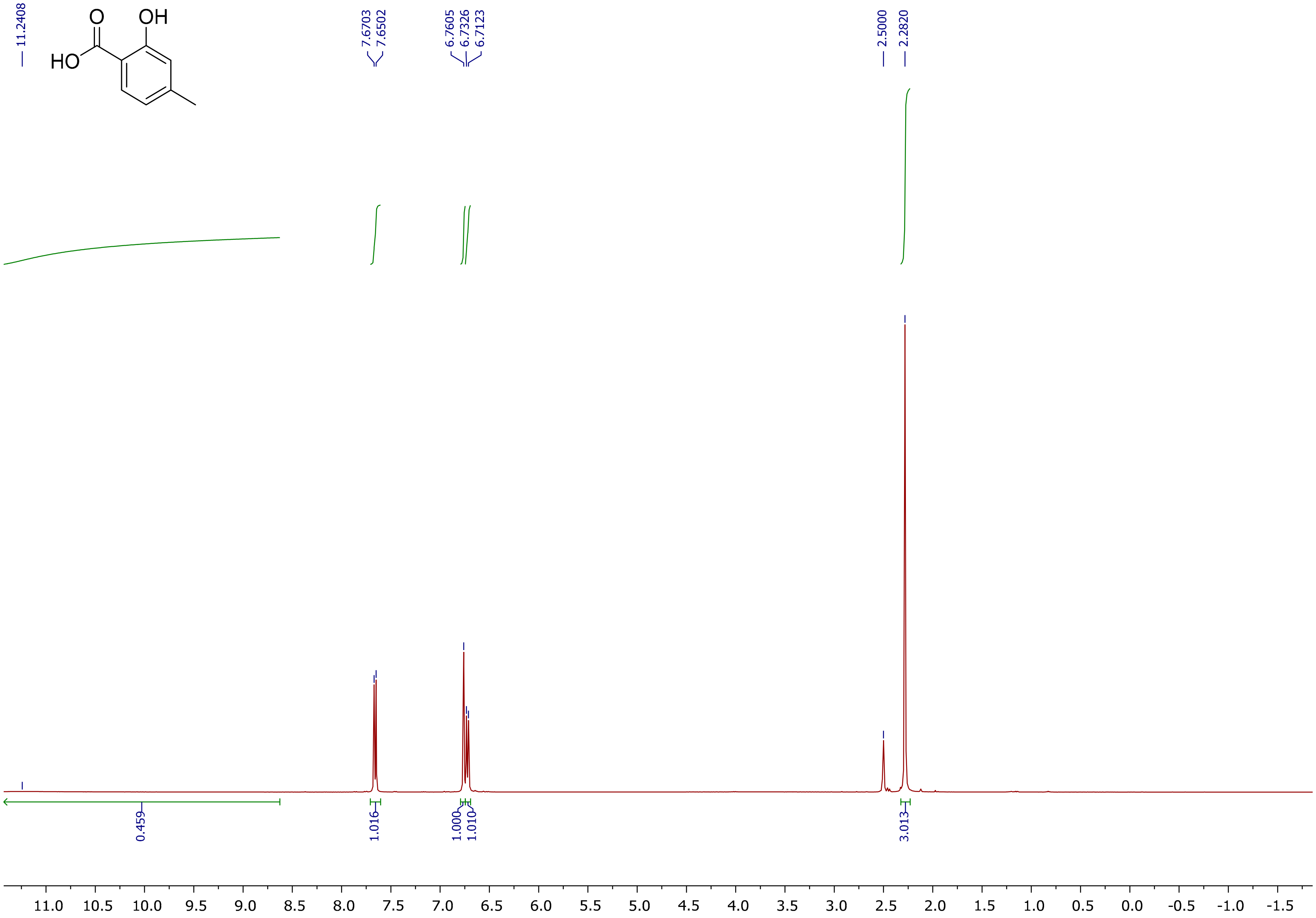

^{13}C NMR (101 MHz, DMSO-d_6) **4o**

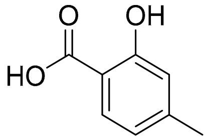

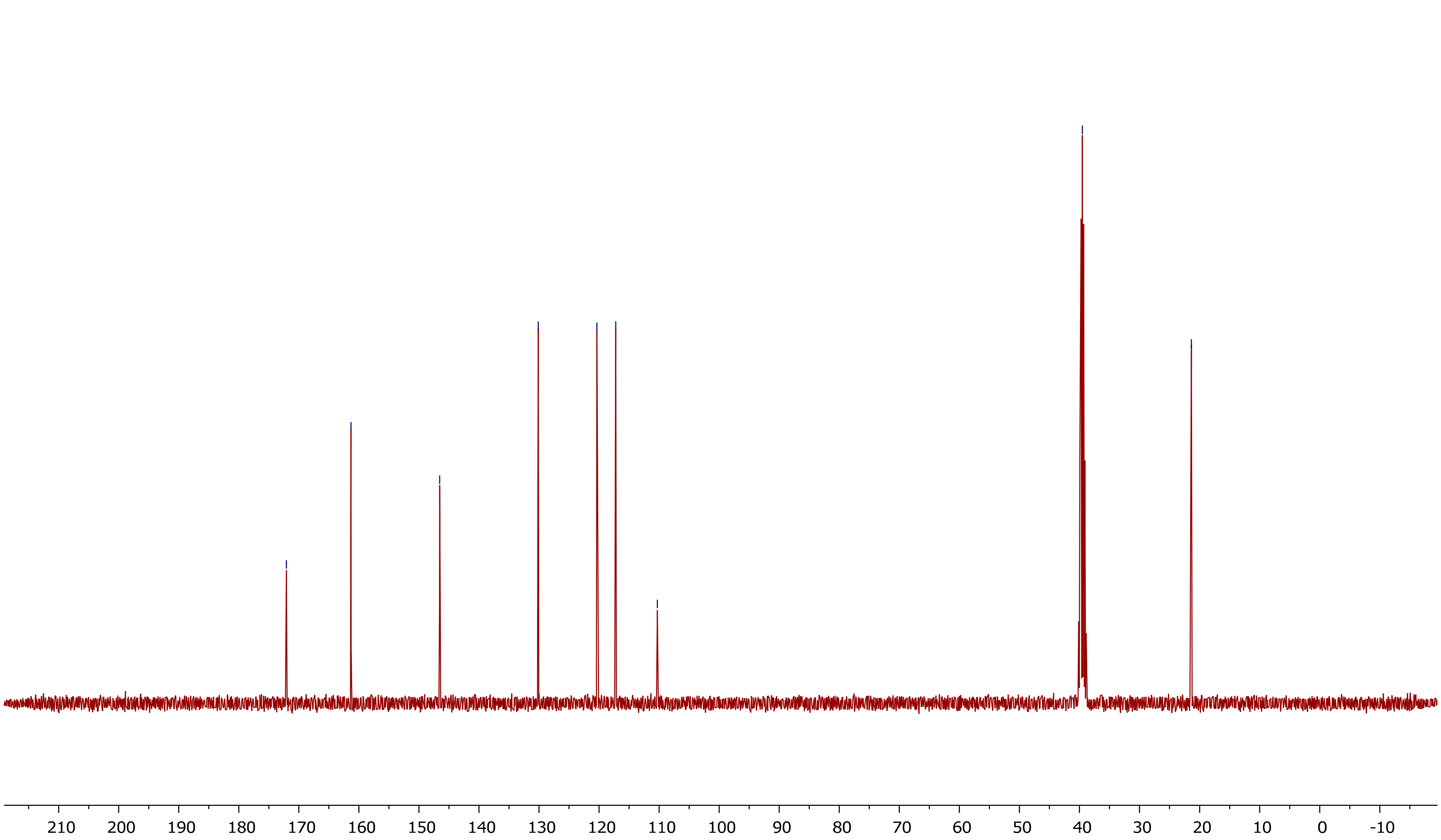

^{1}H NMR (400 MHz, DMSO-d6) **4p**

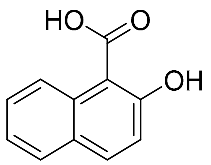

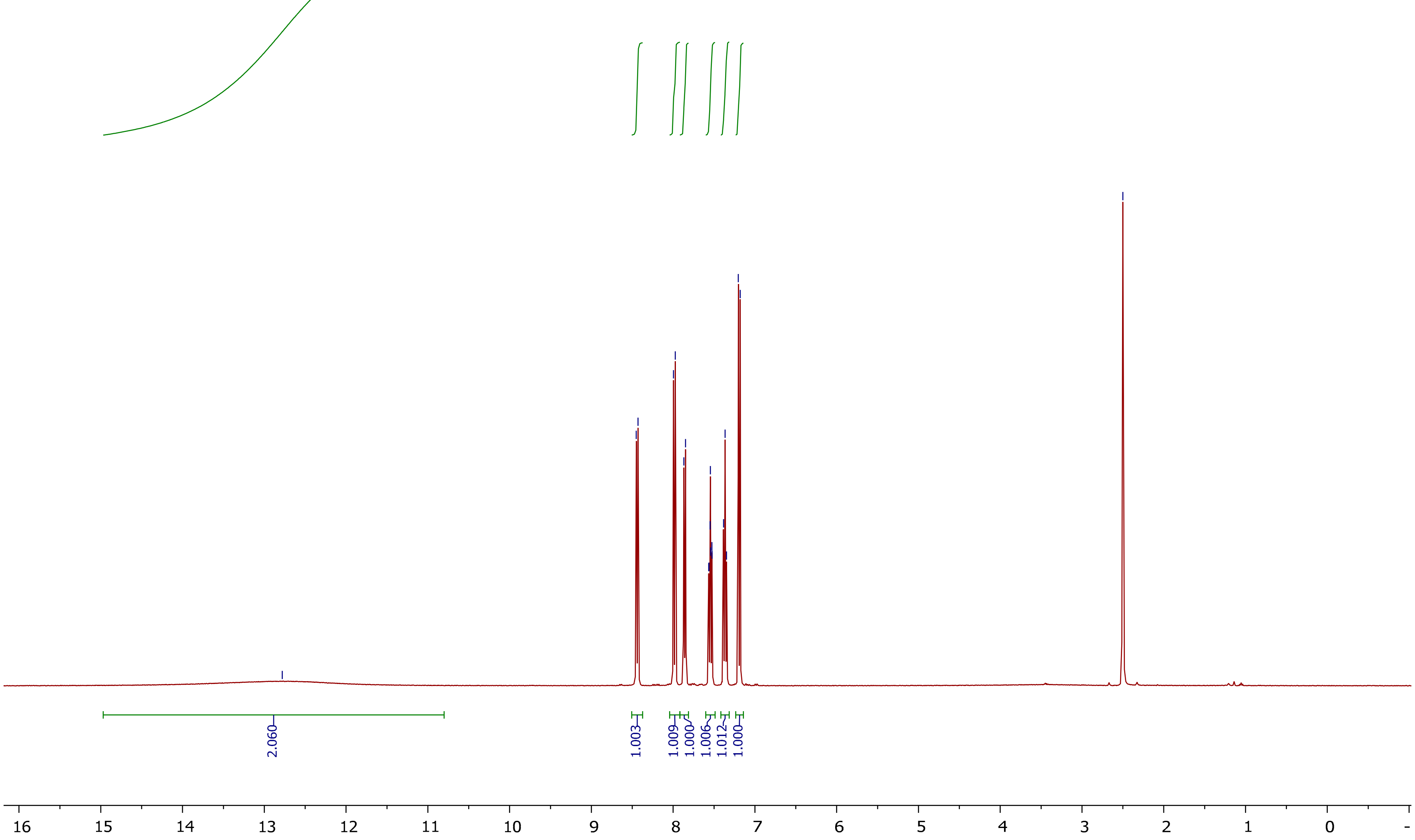

^{1}H NMR (400 MHz, DMSO-d6) **4q**

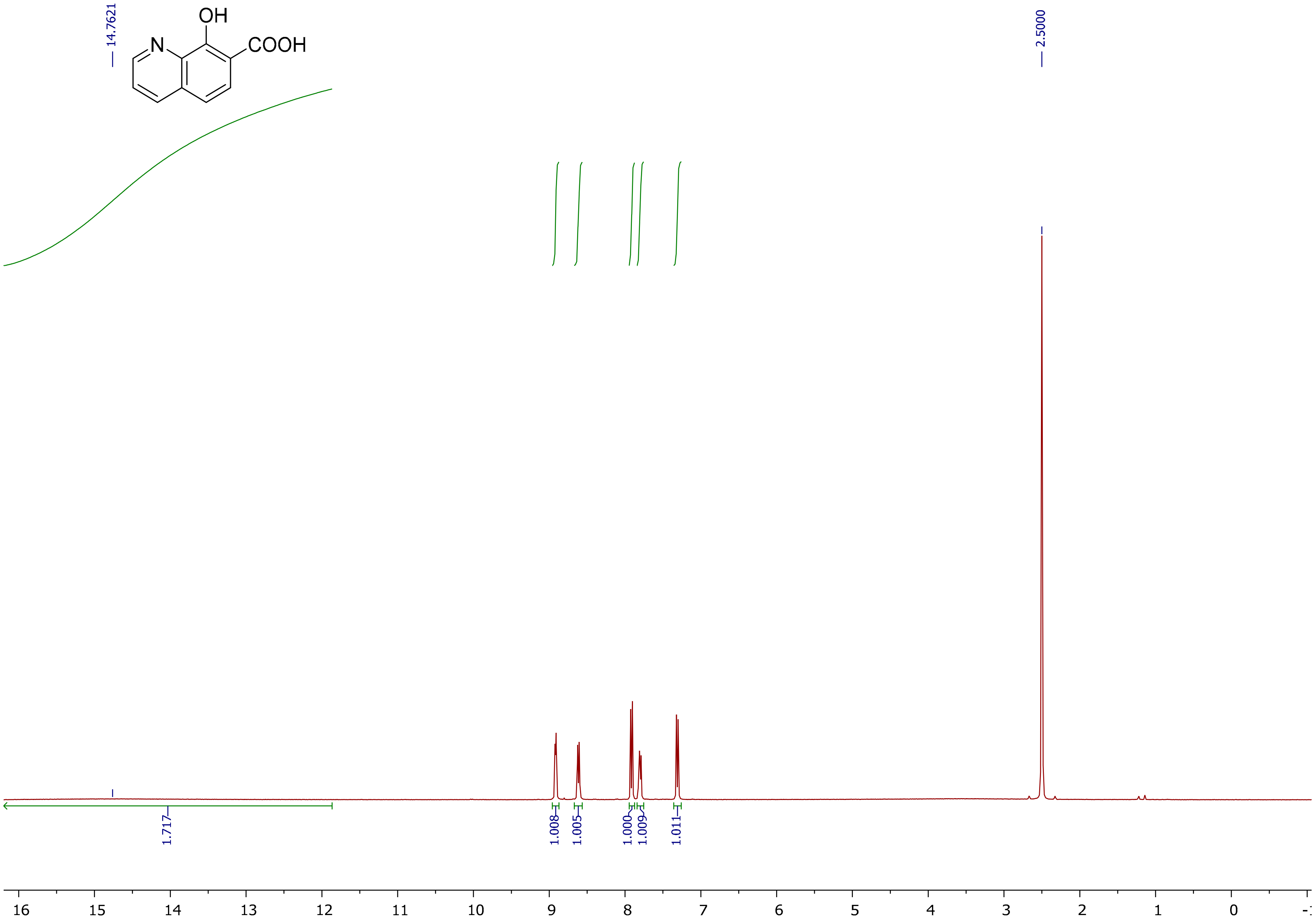

^{1}H NMR (400 MHz, $CDCl_3$)

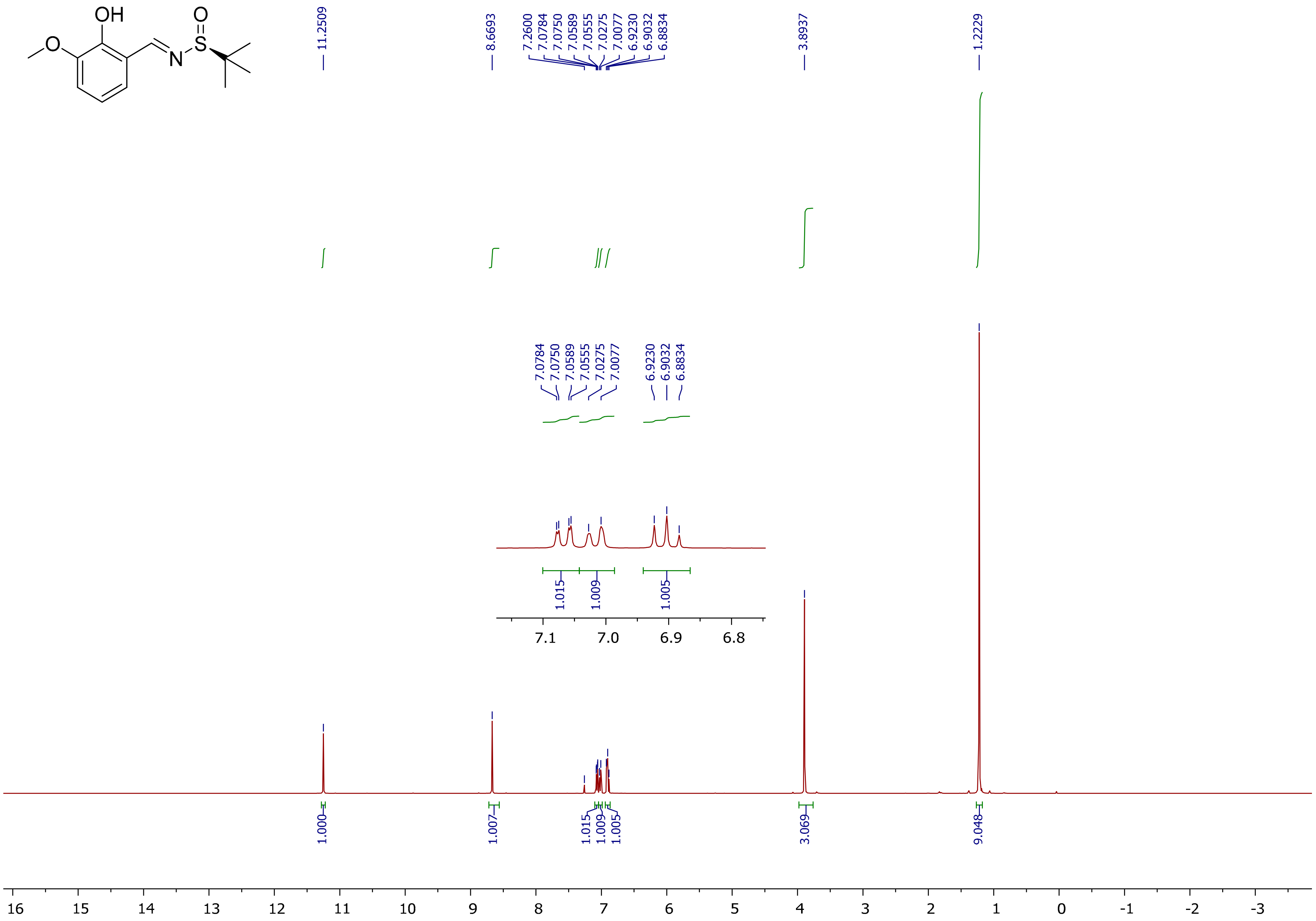

^{13}C NMR (101 MHz, $CDCl_3$)

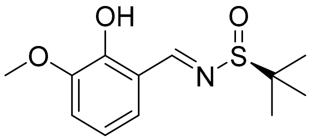

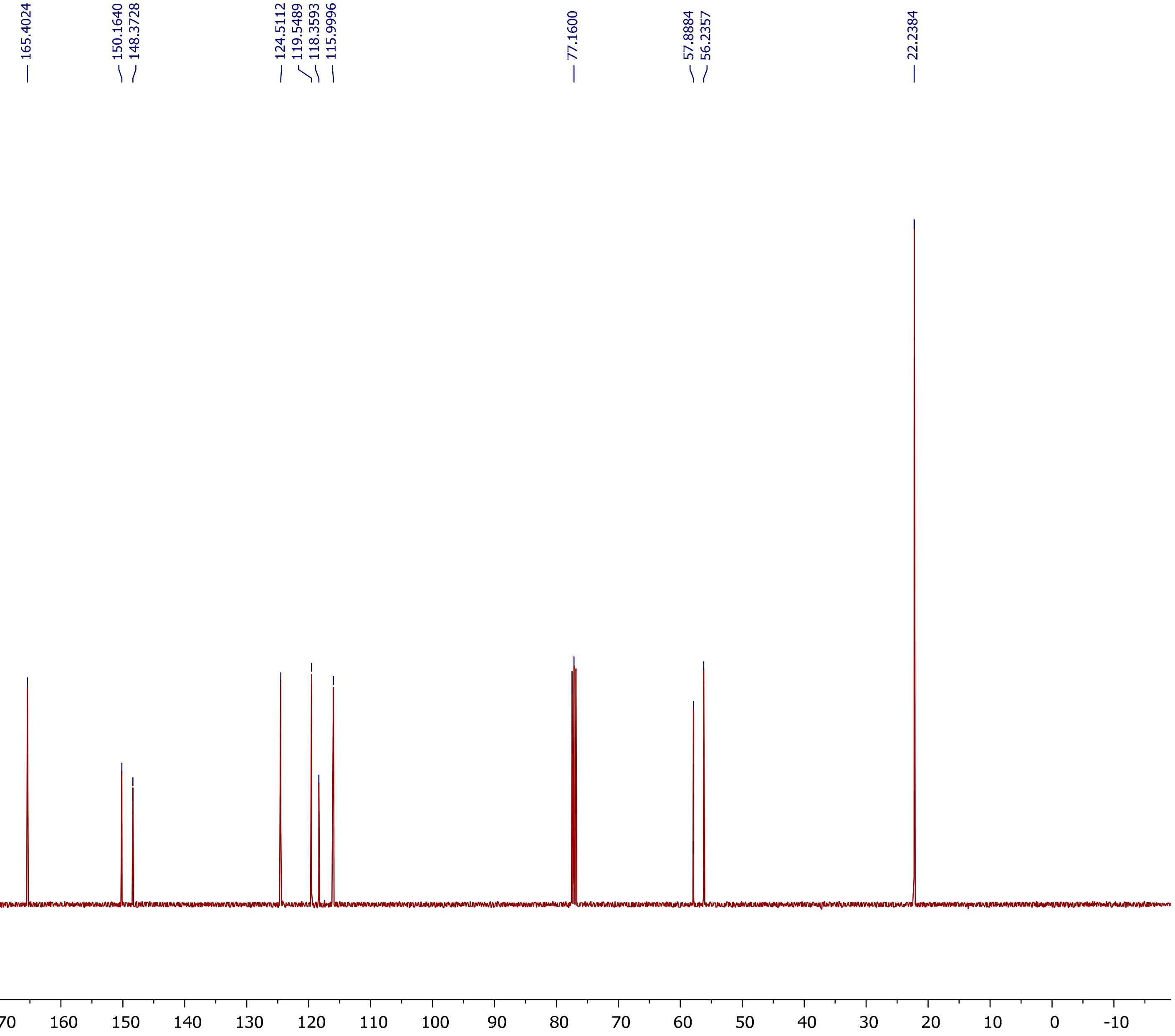

^{1}H NMR ($CDCl_3$, 400 MHz)

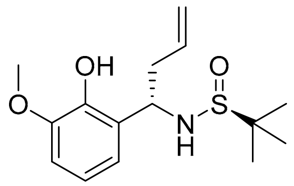

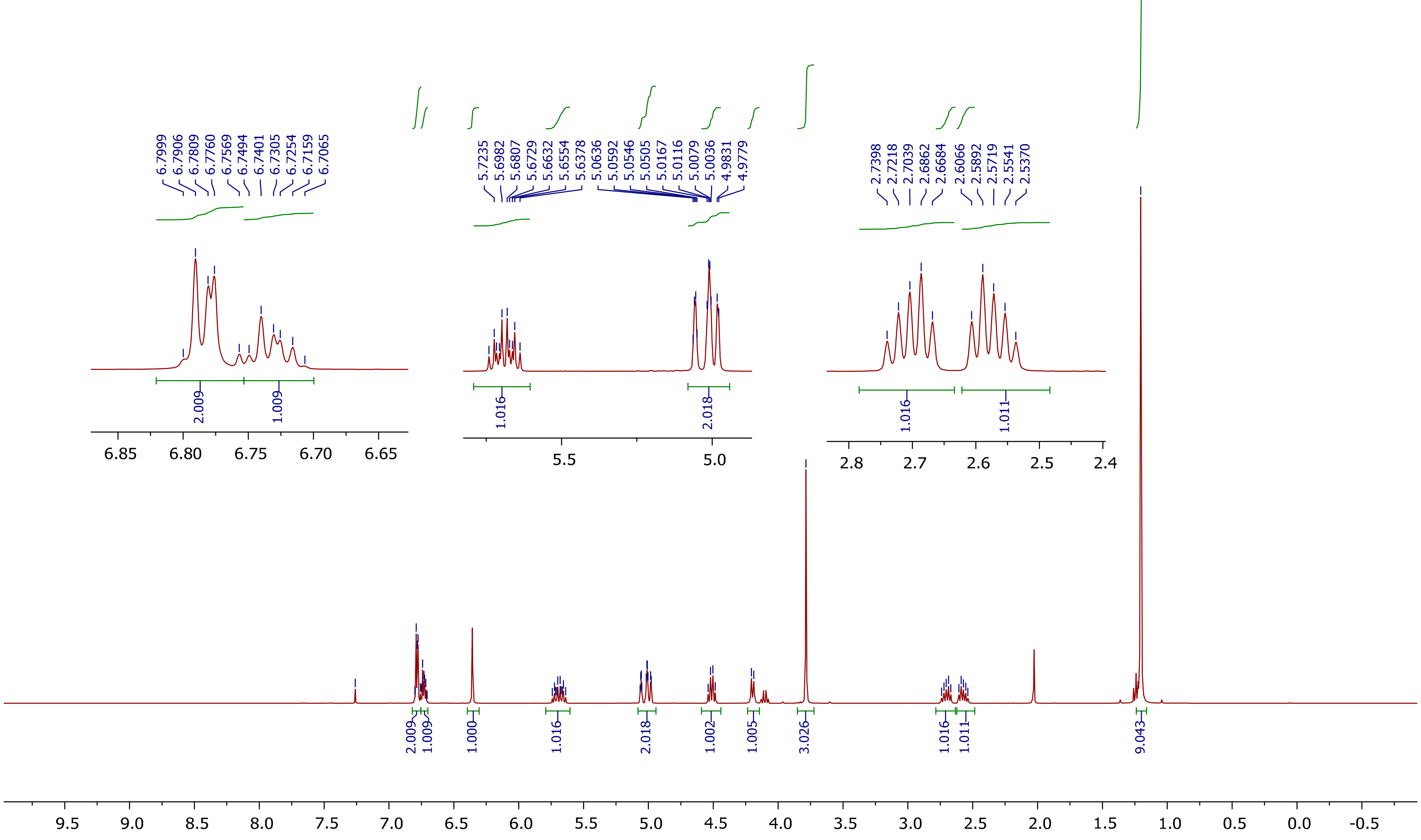

^{13}C NMR ($CDCl_3$, 101 MHz)

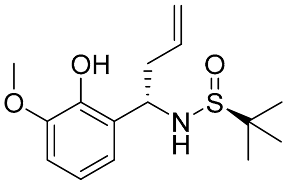

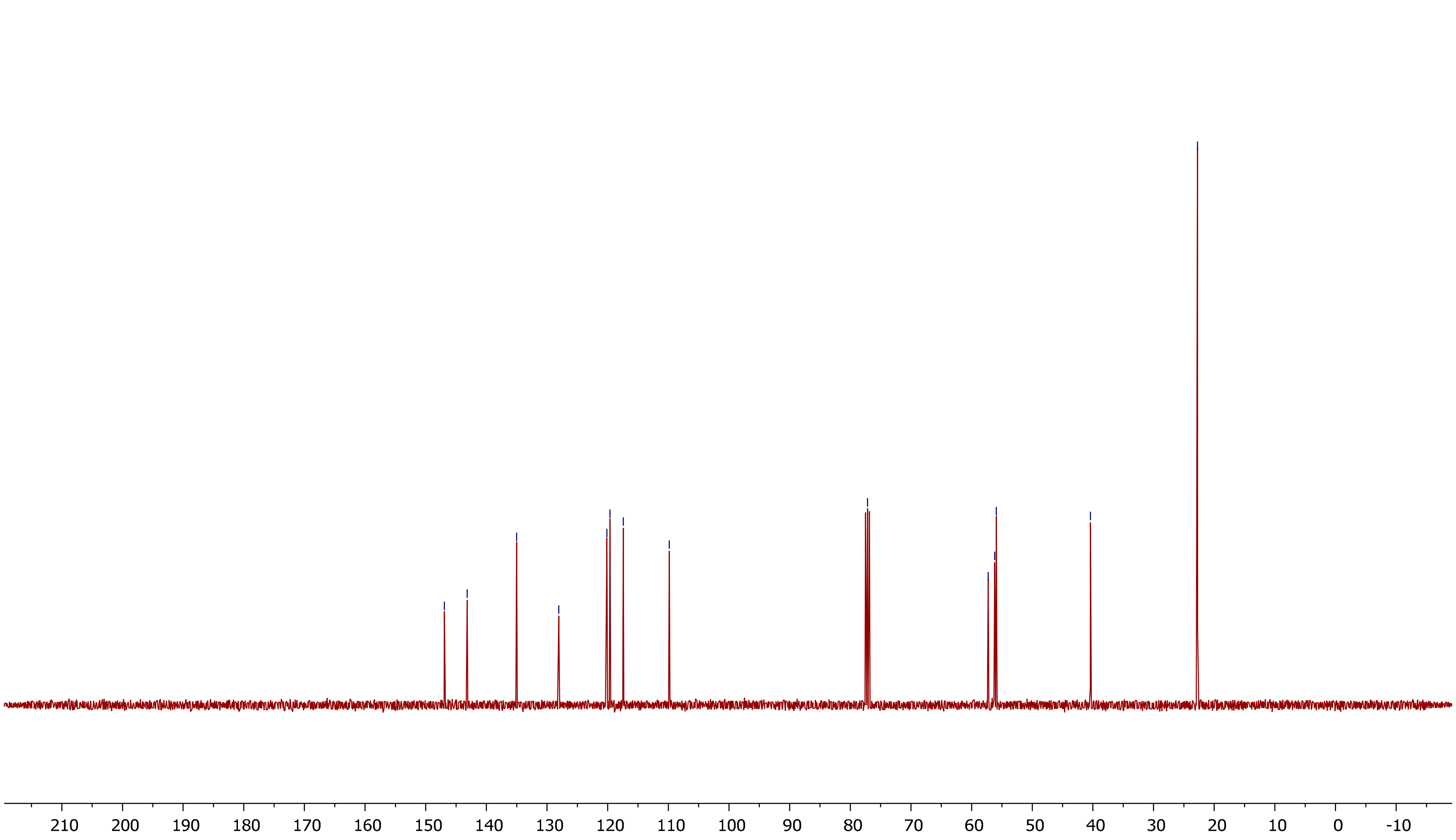

^{1}H NMR (DMSO-d6, 400 MHz) **3r**

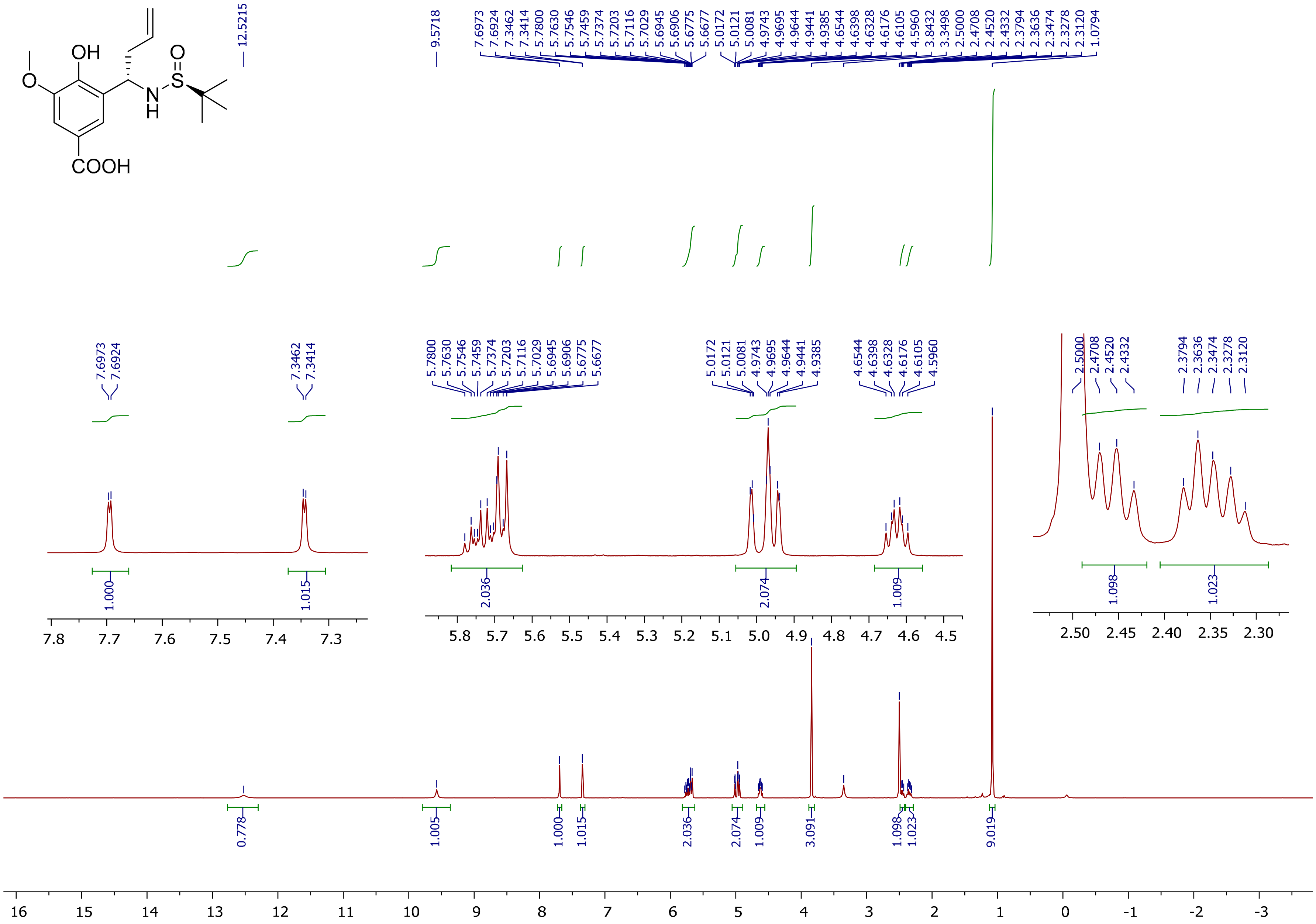

^{13}C NMR (DMSO-d$_6$, 101 MHz) **3r**

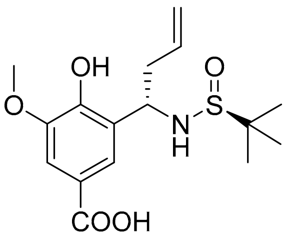

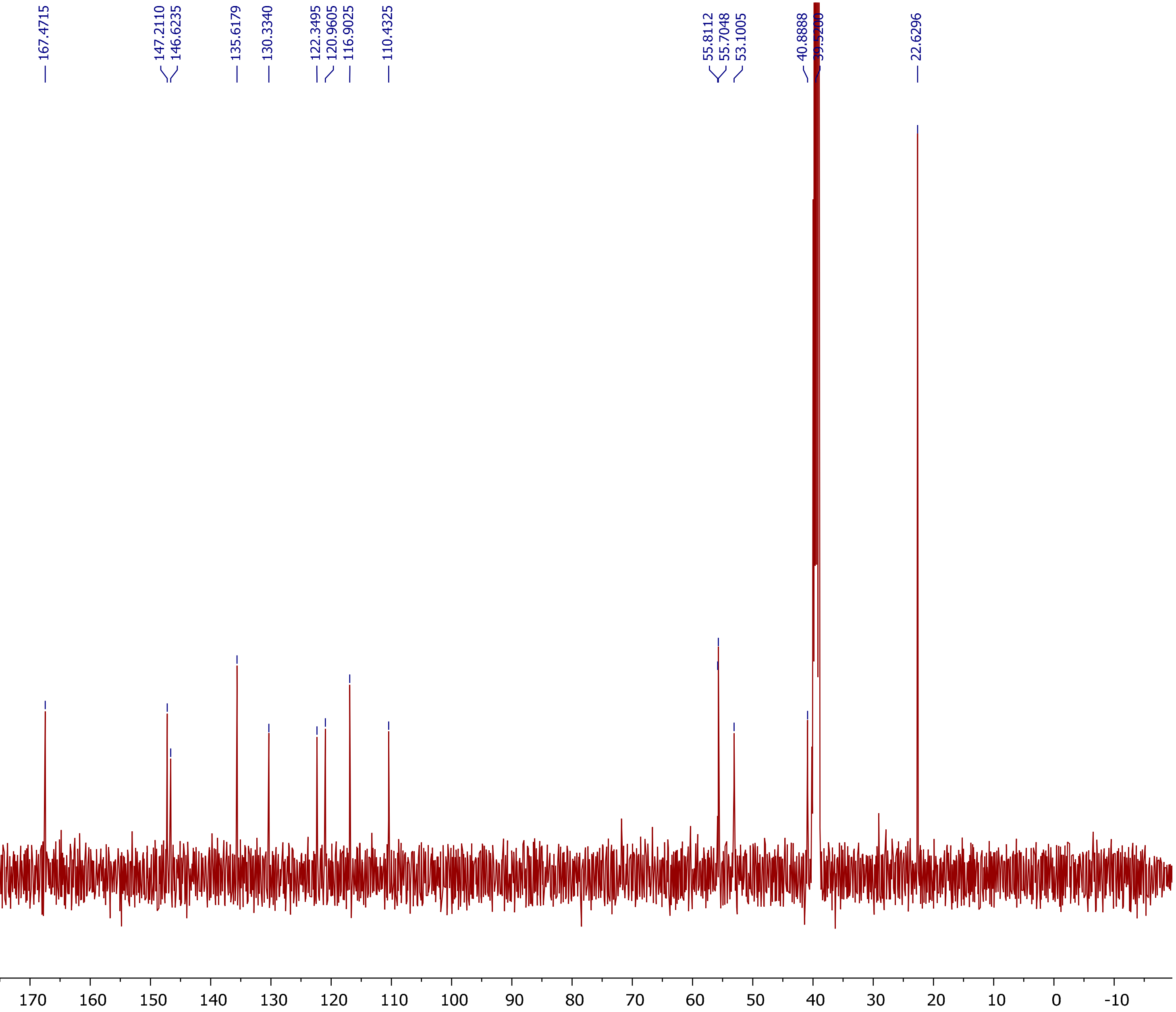

DOSY NMR (DMSO-d_6, 400 MHz) C(PhONa*CO_2) = 0.036M.

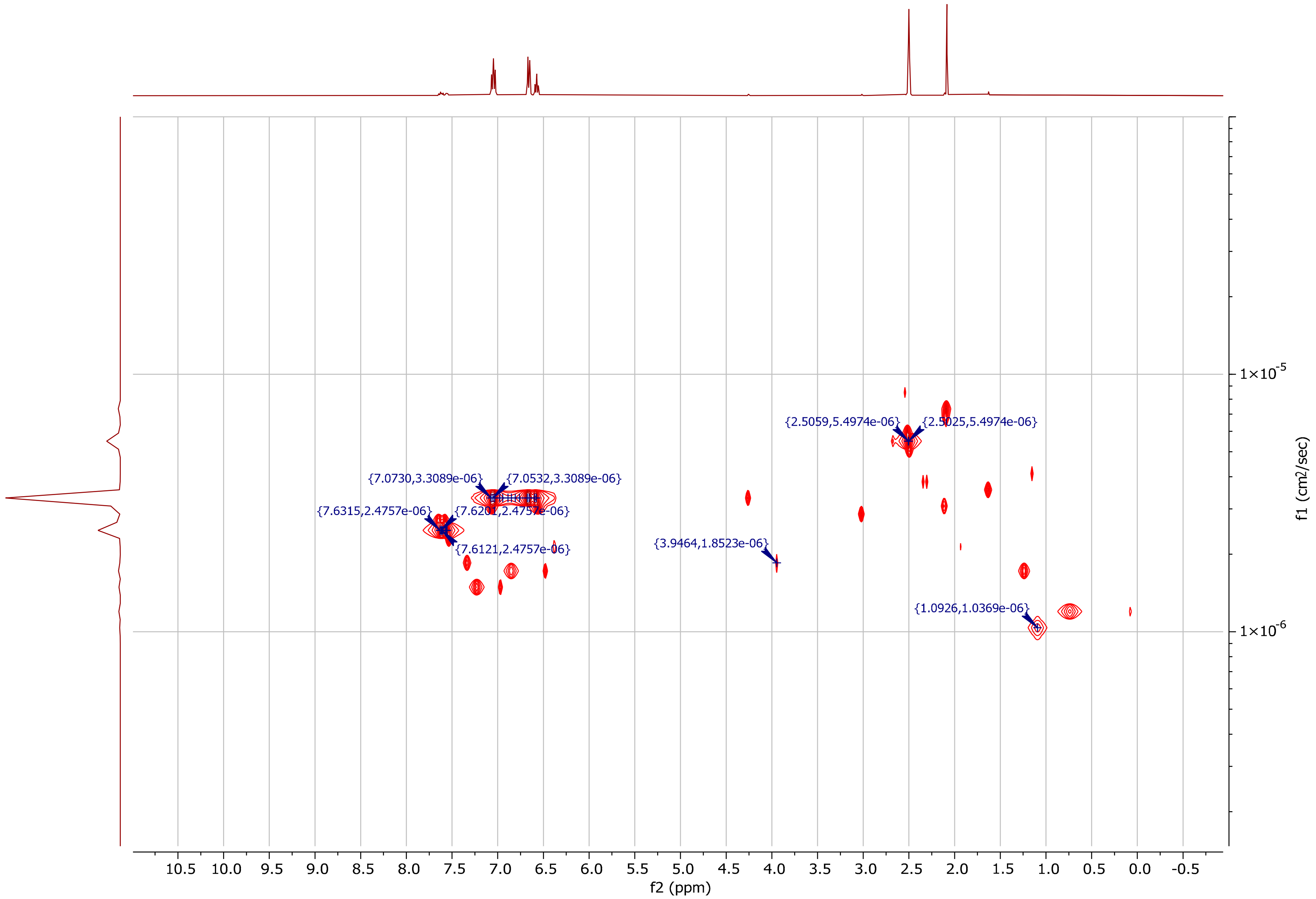

DOSY NMR (DMSO-d_6, 400 MHz) C(PhONa*CO_2) = 0.143M.

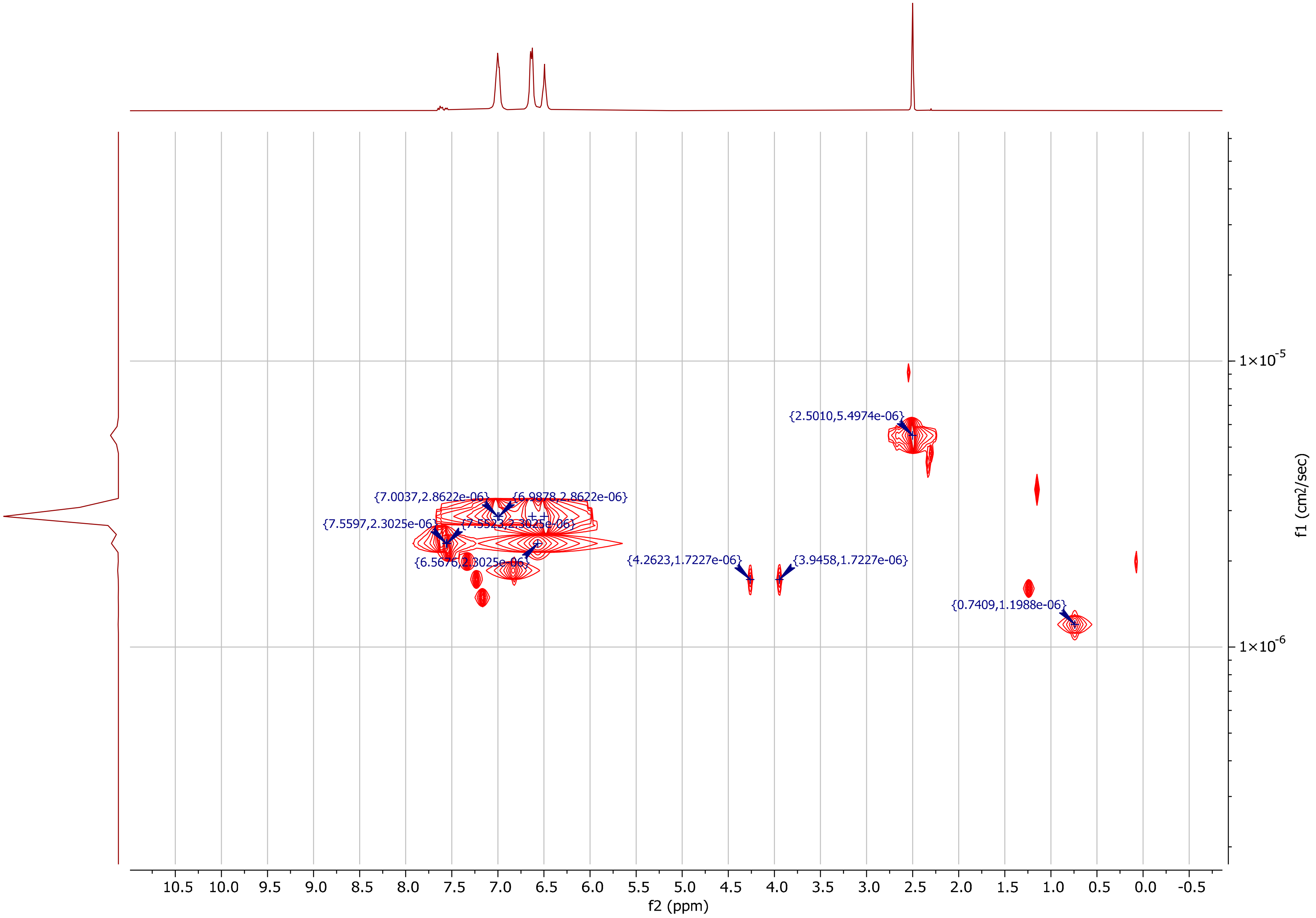

DOSY NMR (DMSO-d6, 400 MHz) C(PhONa*CO_2) = 0.250M.

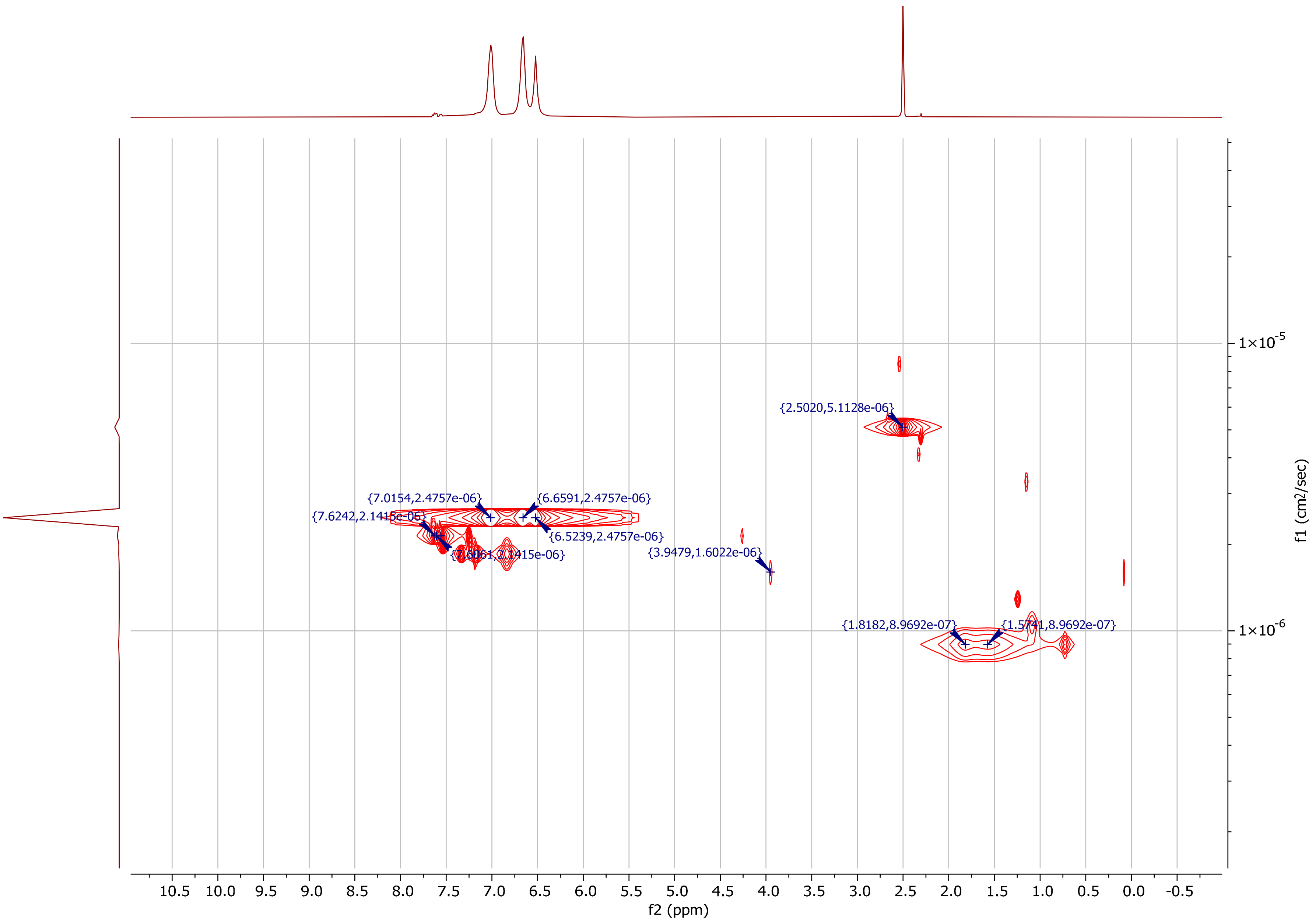

DOSY NMR (DMSO-d6, 400 MHz) C(PhONa*CO2) = 0.357M.

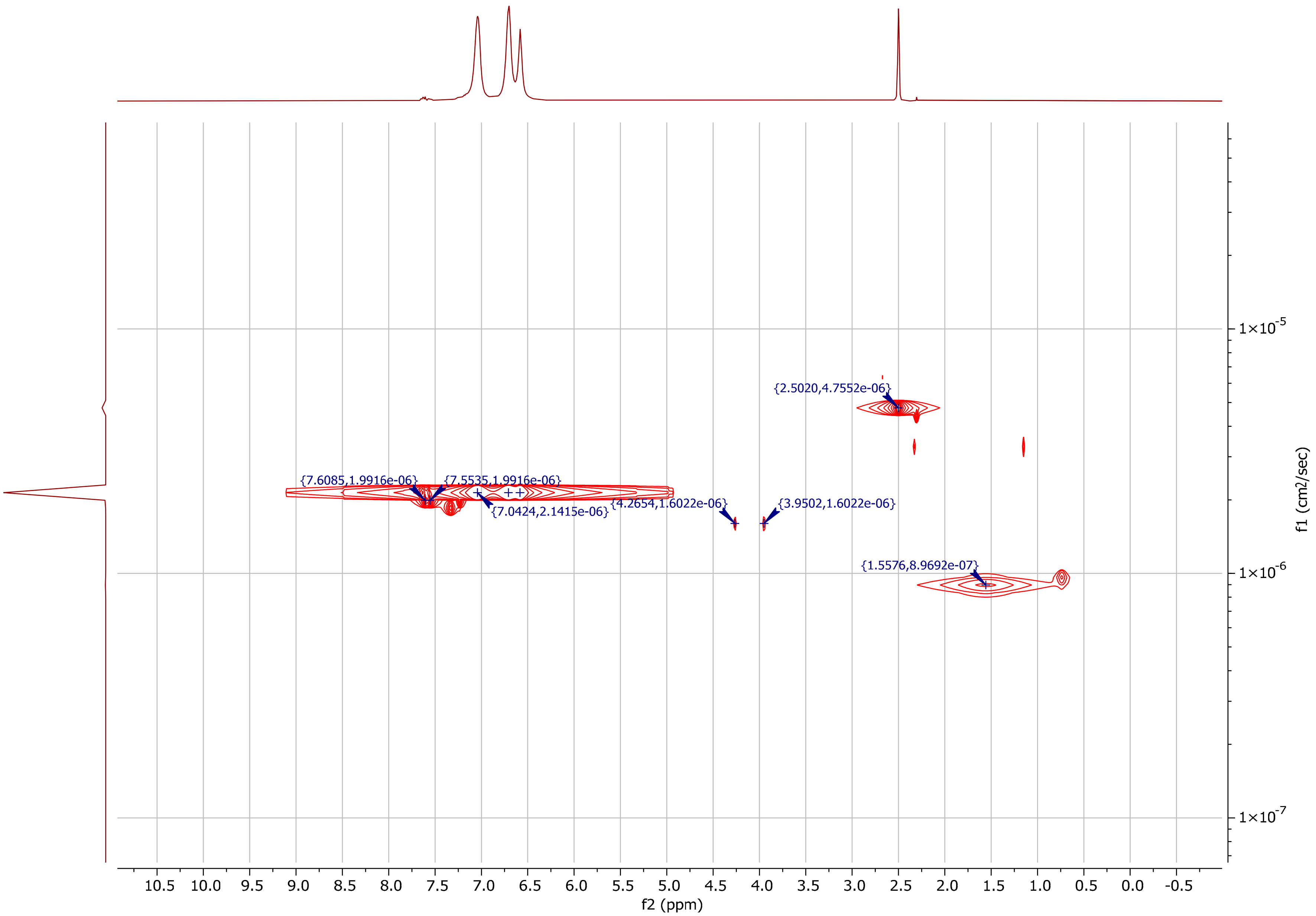

Supplement: Supplementary file 1 [file molecules-31-00239-s001.zip › molecules-4068279-supplementary.pdf]
